# Supplementary material for: A library of base editors for the precise ablation of all protein-coding genes in the mouse mitochondrial genome
Source: Nat Biomed Eng. 2022 Dec 5;7(5):692–703. doi: 10.1038/s41551-022-00968-1 (PMC10195678; doi:10.1038/s41551-022-00968-1)
Supplement: Supplementary file 1 — Supplementary tables and lists of amino acid sequences. [file 41551_2022_968_MOESM1_ESM.pdf]

nature biomedical engineering

Article

https://doi.org/10.1038/s41551-022-00968-1

A library of base editors for the precise ablation of all protein-coding genes in the mouse mitochondrial genome

In the format provided by the authors and unedited

Supplementary Table 1 | Primers for amplification and sequencing of mouse mtDNA genes

Target	Forward primer	Reverse primer
Nd1	GTTGGTCCATACGGCATTTT *	TGAAATTGTTTGGGCTACGG
Nd2	TCTCCGTGCTACCTAAACACC *	GTACGATGGCCAGGAGGATA
Nd3	CGTCTCCATTTATTGATGAGGAT *	TGAATTGCTCATGGTAGTGGA
Nd4	TACCAATCCCCATCACCATC *	GGATAGGGGGTCTGAGGAGA
Nd4l	TACCAATCCCCATCACCATC *	GGGGTCTGAGGAGAATATATTTGA
Nd5	CTTCCCACTGTACACCACCA *	GGCTGAGGTGAGGATAAGCA
Nd6	GCTACCCCAATCCCTCCTT *	TGGTTTTAGGGTTTGGTGGA
Cytb	ATTGACCTACCTGCCCCATC *	TCCTCATGGAAGGACGTAGC
CoI	TGGCGGTAGAAGTCTTAGTAGAG *	AAAGCATGGGCAGTTACGAT
CoII	TGCACAAGAAGTTGAAACCA	AAGTCCTAGGGAGGGGACTG *
CoIII	AGCCTCGTACCAACACATGA *	ATGGAATCCAGTAGCCATGAA
Atp6	GGCACCTTCACCAAAATCAC *	TACGGCTCCAGCTCATAGTG
Atp8	AGCTTTATGCCCATTGTCCT *	GGGGTAATGAATGAGGCAAA

* primer used for Sanger sequencing.

Supplementary Sequences 1 | Amino acid sequences of MitoKO DdCBE library.

The halves used to target the L-strand - DdCBE (L) – have the general architecture of (from N- to C-terminus): *Cox8* MTS – 3xFLAG – TALE – 2aa linker – DddA$_{tox}$ split – 4aa linker – UGI*

- **DdCBE (L) – Nd1-L1 - G1333-N:**

MASVLTPLLLRGLTGSARRLPVPRAKIHSLDYKDHDGDYKDHDIDYKDDDDKMDIADLRTLG
YSQQQQEKIKPKVRSTVAQHHEALVGHGFTHAHIVALSQHPAALGTVAVKYQDMIAALPEA
THEAIVGVGKQWSGARALEALLTVAGELRGPPLQLDTGQLLKIAKRGGVTAVEAVHAWRN
ALTGAPLNLTPQQVVAIASHDGGKQALETVQRLLPVLCQAHGLTPAQVVAIASNIGGKQALE
TVQRLLPVLCQAHGLTPDQVVAIASHDGGKQALETVQRLLPVLCQAHGLTPAQVVAIASNG
GGKQALETVQRLLPVLCQAHGLTPEQVVAIASHDGGKQALETVQRLLPVLCQAHGLTPAQV
VAIASNIGGKQALETVQRLLPVLCQAHGLTPDQVVAIASHDGGKQALETVQRLLPVLCQAH
GLTPAQVVAIASNIGGKQALETVQRLLPVLCQAHGLTPDQVVAIASHDGGKQALETVQRLLP
VLCQAHGLTPAQVVAIASNGGGKQALETVQRLLPVLCQAHGLTPEQVVAIASNIGGKQALE
TVQRLLPVLCQAHGLTPDQVVAIASNNGGKQALETVQRLLPVLCQAHGLTPEQVVAIASHD
GGKQALETVQRLLPVLCQAHGLTPAQVVAIASNIGGKQALETVQRLLPVLCQAHGLTPDQV
VAIASNGGGKQALETVQRLLPVLCQAHGLTPEQVVAIASNGGGRPALESIVAQLSRPDPALA
ALTNDHLVALACLGGRPALDAVKKGLGGSGSYALGPYQISAPQLPAYNGQTVGTFYYVND
AGGLESKVFSSGGSGGSTNLSDIIEKETGKQLVIQESILMLPEEVEEVIGNKPESDILVHTAY
DESTDENVMLLTSDAPEYKPWALVIQDSNGENKIKML*

- **DdCBE (L) – Nd1-L2 - G1333-N:**

MASVLTPLLLRGLTGSARRLPVPRAKIHSLDYKDHDGDYKDHDIDYKDDDDKMDIADLRTLG
YSQQQQEKIKPKVRSTVAQHHEALVGHGFTHAHIVALSQHPAALGTVAVKYQDMIAALPEA
THEAIVGVGKQWSGARALEALLTVAGELRGPPLQLDTGQLLKIAKRGGVTAVEAVHAWRN
ALTGAPLNLTPQQVVAIASHDGGKQALETVQRLLPVLCQAHGLTPAQVVAIASNIGGKQALE
TVQRLLPVLCQAHGLTPDQVVAIASHDGGKQALETVQRLLPVLCQAHGLTPAQVVAIASNIG
GKQALETVQRLLPVLCQAHGLTPDQVVAIASHDGGKQALETVQRLLPVLCQAHGLTPAQVV
AIASNGGGKQALETVQRLLPVLCQAHGLTPEQVVAIASNIGGKQALETVQRLLPVLCQAHGL
TPDQVVAIASNNGGKQALETVQRLLPVLCQAHGLTPEQVVAIASHDGGKQALETVQRLLPV
LCQAHGLTPAQVVAIASNIGGKQALETVQRLLPVLCQAHGLTPDQVVAIASNGGGKQALET
VQRLLPVLCQAHGLTPEQVVAIASNGGGKQALETVQRLLPVLCQAHGLTPEQVVAIASNIG
GRPALESIVAQLSRPDPALAALTNDHLVALACLGGRPALDAVKKGLGGSGSYALGPYQISA
PQLPAYNGQTVGTFYYVNDAGGLESKVFSSGGSGGSTNLSDIIEKETGKQLVIQESILMLPE
EVEEVIGNKPESDILVHTAYDESTDENVMLLTSDAPEYKPWALVIQDSNGENKIKML*

- **DdCBE (L) – Nd2-L1 - G1333-N:**

MASVLTPLLLRGLTGSARRLPVPRAKIHSLDYKDHDGDYKDHDIDYKDDDDKMDIADLRTLG
YSQQQQEKIKPKVRSTVAQHHEALVGHGFTHAHIVALSQHPAALGTVAVKYQDMIAALPEA
THEAIVGVGKQWSGARALEALLTVAGELRGPPLQLDTGQLLKIAKRGGVTAVEAVHAWRN
ALTGAPLNLTPQQVVAIASHDGGKQALETVQRLLPVLCQAHGLTPAQVVAIASHDGGKQAL
ETVQRLLPVLCQAHGLTPAQVVAIASNIGGKQALETVQRLLPVLCQAHGLTPDQVVAIASNN
GGKQALETVQRLLPVLCQAHGLTPEQVVAIASHDGGKQALETVQRLLPVLCQAHGLTPAQV
VAIASNIGGKQALETVQRLLPVLCQAHGLTPDQVVAIASHDGGKQALETVQRLLPVLCQAH
GLTPAQVVAIASHDGGKQALETVQRLLPVLCQAHGLTPAQVVAIASNIGGKQALETVQRLLP
VLCQAHGLTPDQVVAIASNIGGKQALETVQRLLPVLCQAHGLTPDQVVAIASHDGGKQALE
TVQRLLPVLCQAHGLTPAQVVAIASHDGGKQALETVQRLLPVLCQAHGLTPAQVVAIASNG
GGKQALETVQRLLPVLCQAHGLTPEQVVAIASNIGGKQALETVQRLLPVLCQAHGLTPDQV
VAIASNIGGKQALETVQRLLPVLCQAHGLTPDQVVAIASNGGGRPALESIVAQLSRPDPALA
ALTNDHLVALACLGGRPALDAVKKGLGGSGSYALGPYQISAPQLPAYNGQTVGTFYYVND
AGGLESKVFSSGGSGGSTNLSDIIEKETGKQLVIQESILMLPEEVEEVIGNKPESDILVHTAY
DESTDENVMLLTSDAPEYKPWALVIQDSNGENKIKML*

- **DdCBE (L) – Nd2-L2 - G1333-N:**

MASVLTPLLLRGLTGSARRLPVPRAKIHSLDYKDHDGDYKDHDIDYKDDDDKMDIADLRTLG
YSQQQQEKIKPKVRSTVAQHHEALVGHGFTHAHIVALSQHPAALGTVAVKYQDMIAALPEA
THEAIVGVGKRGAGARALEALLTVAGELRGPPLQLDTGQLLKIAKRGGVTAVEAVHAWRNA
LTGAPLNLTPQQVVAIASNNGGKQALETVQRLLPVLCQAHGLTPEQVVAIASHDGGKQALE
TVQRLLPVLCQAHGLTPAQVVAIASNIGGKQALETVQRLLPVLCQAHGLTPDQVVAIASHDG
GKQALETVQRLLPVLCQAHGLTPAQVVAIASHDGGKQALETVQRLLPVLCQAHGLTPAQVV
AIASNIGGKQALETVQRLLPVLCQAHGLTPDQVVAIASNIGGKQALETVQRLLPVLCQAHGL
TPDQVVAIASHDGGKQALETVQRLLPVLCQAHGLTPAQVVAIASHDGGKQALETVQRLLPV
LCQAHGLTPAQVVAIASNGGGKQALETVQRLLPVLCQAHGLTPEQVVAIASNIGGKQALET
VQRLLPVLCQAHGLTPDQVVAIASNIGGKQALETVQRLLPVLCQAHGLTPDQVVAIASNGG
GKQALETVQRLLPVLCQAHGLTPEQVVAIASNIGGRPALESIVAQLSRPDPALAALTNDHLV
ALACLGGRPALDAVKKGLGGSGSYALGPYQISAPQLPAYNGQTVGTFYYVNDAGGLESKV
FSSGGSGGSTNLSDIIEKETGKQLVIQESILMLPEEVEEVIGNKPESDILVHTAYDESTDENV
MLLTSDAPEYKPWALVIQDSNGENKIKML*

- **DdCBE (L) – Nd3-L1 - G1333-N:**

MASVLTPLLLRGLTGSARRLPVPRAKIHSLDYKDHDGDYKDHDIDYKDDDDKMDIADLRTLG
YSQQQQEKIKPKVRSTVAQHHEALVGHGFTHAHIVALSQHPAALGTVAVKYQDMIAALPEA
THEAIVGVGKQWSGARALEALLTVAGELRGPPLQLDTGQLLKIAKRGGVTAVEAVHAWRN
ALTGAPLNLTPQQVVAIASHDGGKQALETVQRLLPVLCQAHGLTPAQVVAIASHDGGKQAL
ETVQRLLPVLCQAHGLTPAQVVAIASHDGGKQALETVQRLLPVLCQAHGLTPAQVVAIASN
GGGKQALETVQRLLPVLCQAHGLTPEQVVAIASNIGGKQALETVQRLLPVLCQAHGLTPDQ
VVAIASNIGGKQALETVQRLLPVLCQAHGLTPDQVVAIASHDGGKQALETVQRLLPVLCQAH
GLTPAQVVAIASNNGGKQALETVQRLLPVLCQAHGLTPEQVVAIASHDGGKQALETVQRLL
PVLCQAHGLTPAQVVAIASNGGGKQALETVQRLLPVLCQAHGLTPEQVVAIASNIGGKQAL
ETVQRLLPVLCQAHGLTPDQVVAIASNIGGKQALETVQRLLPVLCQAHGLTPDQVVAIASNG
GGKQALETVQRLLPVLCQAHGLTPEQVVAIASNGGGKQALETVQRLLPVLCQAHGLTPEQ
VVAIASHDGGKQALETVQRLLPVLCQAHGLTPAQVVAIASNGGGKQALETVQRLLPVLCQA
HGLTPEQVVAIASNIGGRPALESIVAQLSRPDPALAALTNDHLVALACLGGRPALDAVKKGL
GGSGSYALGPYQISAPQLPAYNGQTVGTFYYVNDAGGLESKVFSSGGSGGSTNLSDIIEKE
TGKQLVIQESILMLPEEVEEVIGNKPESDILVHTAYDESTDENVMLLTSDAPEYKPWALVIQD
SNGENKIKML*

- **DdCBE (L) – Nd3-L2 - G1333-N:**

MASVLTPLLLRGLTGSARRLPVPRAKIHSLDYKDHDGDYKDHDIDYKDDDDKMDIADLRTLG
YSQQQQEKIKPKVRSTVAQHHEALVGHGFTHAHIVALSQHPAALGTVAVKYQDMIAALPEA
THEAIVGVGKQWSGARALEALLTVAGELRGPPLQLDTGQLLKIAKRGGVTAVEAVHAWRN
ALTGAPLNLTPQQVVAIASNIGGKQALETVQRLLPVLCQAHGLTPDQVVAIASNIGGKQALE
TVQRLLPVLCQAHGLTPDQVVAIASHDGGKQALETVQRLLPVLCQAHGLTPAQVVAIASNN
GGKQALETVQRLLPVLCQAHGLTPEQVVAIASHDGGKQALETVQRLLPVLCQAHGLTPAQV
VAIASNGGGKQALETVQRLLPVLCQAHGLTPEQVVAIASNIGGKQALETVQRLLPVLCQAH
GLTPDQVVAIASNIGGKQALETVQRLLPVLCQAHGLTPDQVVAIASNGGGKQALETVQRLLP
VLCQAHGLTPEQVVAIASNGGGKQALETVQRLLPVLCQAHGLTPEQVVAIASHDGGKQALE
TVQRLLPVLCQAHGLTPAQVVAIASNGGGKQALETVQRLLPVLCQAHGLTPEQVVAIASNIG
GKQALETVQRLLPVLCQAHGLTPDQVVAIASNNGGRPALESIVAQLSRPDPALAALTNDHLV
ALACLGGRPALDAVKKGLGGSGSYALGPYQISAPQLPAYNGQTVGTFYYVNDAGGLESKV
FSSGGSGGSTNLSDIIEKETGKQLVIQESILMLPEEVEEVIGNKPESDILVHTAYDESTDENV
MLLTSDAPEYKPWALVIQDSNGENKIKML*

- **DdCBE (L) – Nd4-L1 - G1333-N:**

MASVLTPLLLRGLTGSARRLPVPRAKIHSLDYKDHDGDYKDHDIDYKDDDDKMDIADLRTLG
YSQQQQEKIKPKVRSTVAQHHEALVGHGFTHAHIVALSQHPAALGTVAVKYQDMIAALPEA
THEAIVGVGKQWSGARALEALLTVAGELRGPPLQLDTGQLLKIAKRGGVTAVEAVHAWRN
ALTGAPLNLTPQQVVAIASHDGGKQALETVQRLLPVLCQAHGLTPAQVVAIASNIGGKQALE
TVQRLLPVLCQAHGLTPDQVVAIASHDGGKQALETVQRLLPVLCQAHGLTPAQVVAIASNG
GGKQALETVQRLLPVLCQAHGLTPEQVVAIASNIGGKQALETVQRLLPVLCQAHGLTPDQV
VAIASNIGGKQALETVQRLLPVLCQAHGLTPDQVVAIASNGGGKQALETVQRLLPVLCQAH
GLTPEQVVAIASNNGGKQALETVQRLLPVLCQAHGLTPEQVVAIASHDGGKQALETVQRLL
PVLCQAHGLTPAQVVAIASNGGGKQALETVQRLLPVLCQAHGLTPEQVVAIASNIGGKQAL
ETVQRLLPVLCQAHGLTPDQVVAIASHDGGKQALETVQRLLPVLCQAHGLTPAQVVAIASN
GGGKQALETVQRLLPVLCQAHGLTPEQVVAIASNIGGKQALETVQRLLPVLCQAHGLTPDQ
VVAIASHDGGKQALETVQRLLPVLCQAHGLTPAQVVAIASHDGGRPALESIVAQLSRPDPAL
AALTNDHLVALACLGGRPALDAVKKGLGGSGSYALGPYQISAPQLPAYNGQTVGTFYYVN
DAGGLESKVFSSGGSGGSTNLSDIIEKETGKQLVIQESILMLPEEVEEVIGNKPESDILVHTA
YDESTDENVMLLTSDAPEYKPWALVIQDSNGENKIKML*

- **DdCBE (L) – Nd4-L2 - G1333-N:**

MASVLTPLLLRGLTGSARRLPVPRAKIHSLDYKDHDGDYKDHDIDYKDDDDKMDIADLRTLG
YSQQQQEKIKPKVRSTVAQHHEALVGHGFTHAHIVALSQHPAALGTVAVKYQDMIAALPEA
THEAIVGVGKQWSGARALEALLTVAGELRGPPLQLDTGQLLKIAKRGGVTAVEAVHAWRN
ALTGAPLNLTPQQVVAIASNIGGKQALETVQRLLPVLCQAHGLTPDQVVAIASNIGGKQALE
TVQRLLPVLCQAHGLTPDQVVAIASNGGGKQALETVQRLLPVLCQAHGLTPEQVVAIASNN
GGKQALETVQRLLPVLCQAHGLTPEQVVAIASHDGGKQALETVQRLLPVLCQAHGLTPAQV
VAIASNGGGKQALETVQRLLPVLCQAHGLTPEQVVAIASNIGGKQALETVQRLLPVLCQAH
GLTPDQVVAIASHDGGKQALETVQRLLPVLCQAHGLTPAQVVAIASNGGGKQALETVQRLL
PVLCQAHGLTPEQVVAIASNIGGKQALETVQRLLPVLCQAHGLTPDQVVAIASHDGGKQAL
ETVQRLLPVLCQAHGLTPAQVVAIASHDGGKQALETVQRLLPVLCQAHGLTPAQVVAIASNI
GGRPALESIVAQLSRPDPALAALTNDHLVALACLGGRPALDAVKKGLGGSGSYALGPYQIS
APQLPAYNGQTVGTFYYVNDAGGLESKVFSSGGSGGSTNLSDIIEKETGKQLVIQESILMLP
EEVEEVIGNKPESDILVHTAYDESTDENVMLLTSDAPEYKPWALVIQDSNGENKIKML*

- **DdCBE (L) – Nd4l-L1 - G1333-N:**

MASVLTPLLLRGLTGSARRLPVPRAKIHSLDYKDHDGDYKDHDIDYKDDDDKMDIADLRTLG
YSQQQQEKIKPKVRSTVAQHHEALVGHGFTHAHIVALSQHPAALGTVAVKYQDMIAALPEA
THEAIVGVGKQWSGARALEALLTVAGELRGPPLQLDTGQLLKIAKRGGVTAVEAVHAWRN
ALTGAPLNLTPQQVVAIASHDGGKQALETVQRLLPVLCQAHGLTPAQVVAIASNIGGKQALE
TVQRLLPVLCQAHGLTPDQVVAIASNIGGKQALETVQRLLPVLCQAHGLTPDQVVAIASNIG
GKQALETVQRLLPVLCQAHGLTPDQVVAIASHDGGKQALETVQRLLPVLCQAHGLTPAQVV
AIASNIGGKQALETVQRLLPVLCQAHGLTPDQVVAIASHDGGKQALETVQRLLPVLCQAHGL
TPAQVVAIASNNGGKQALETVQRLLPVLCQAHGLTPEQVVAIASNGGGKQALETVQRLLPV
LCQAHGLTPEQVVAIASNIGGKQALETVQRLLPVLCQAHGLTPDQVVAIASHDGGKQALET
VQRLLPVLCQAHGLTPAQVVAIASNNGGKQALETVQRLLPVLCQAHGLTPEQVVAIASNNG
GKQALETVQRLLPVLCQAHGLTPEQVVAIASNIGGKQALETVQRLLPVLCQAHGLTPDQVV
AIASNIGGKQALETVQRLLPVLCQAHGLTPDQVVAIASHDGGRPALESIVAQLSRPDPALAA
LTNDHLVALACLGGRPALDAVKKGLGGSGSYALGPYQISAPQLPAYNGQTVGTFYYVNDA
GGLESKVFSSGGSGGSTNLSDIIEKETGKQLVIQESILMLPEEVEEVIGNKPESDILVHTAYD
ESTDENVMLLTSDAPEYKPWALVIQDSNGENKIKML*

- **DdCBE (L) – Nd4l-L2 - G1333-N:**

MASVLTPLLLRGLTGSARRLPVPRAKIHSLDYKDHDGDYKDHDIDYKDDDDKMDIADLRTLG
YSQQQQEKIKPKVRSTVAQHHEALVGHGFTHAHIVALSQHPAALGTVAVKYQDMIAALPEA
THEAIVGVGKRGAGARALEALLTVAGELRGPPLQLDTGQLLKIAKRGGVTAVEAVHAWRNA
LTGAPLNLTPQQVVAIASHDGGKQALETVQRLLPVLCQAHGLTPAQVVAIASNIGGKQALET
VQRLLPVLCQAHGLTPDQVVAIASHDGGKQALETVQRLLPVLCQAHGLTPAQVVAIASNNG
GKQALETVQRLLPVLCQAHGLTPEQVVAIASNGGGKQALETVQRLLPVLCQAHGLTPEQVV
AIASNIGGKQALETVQRLLPVLCQAHGLTPDQVVAIASHDGGKQALETVQRLLPVLCQAHGL
TPAQVVAIASNNGGKQALETVQRLLPVLCQAHGLTPEQVVAIASNNGGKQALETVQRLLPV
LCQAHGLTPEQVVAIASNIGGKQALETVQRLLPVLCQAHGLTPDQVVAIASNIGGKQALETV
QRLLPVLCQAHGLTPDQVVAIASHDGGKQALETVQRLLPVLCQAHGLTPAQVVAIASNIGG
KQALETVQRLLPVLCQAHGLTPDQVVAIASNNGGRPALESIVAQLSRPDPALAALTNDHLVA
LACLGGRPALDAVKKGLGGSGSYALGPYQISAPQLPAYNGQTVGTFYYVNDAGGLESKVF
SSGGSGGSTNLSDIIEKETGKQLVIQESILMLPEEVEEVIGNKPESDILVHTAYDESTDENVM
LLTSDAPEYKPWALVIQDSNGENKIKML*

- **DdCBE (L) – Nd5-L1 - G1333-N:**

MASVLTPLLLRGLTGSARRLPVPRAKIHSLDYKDHDGDYKDHDIDYKDDDDKMDIADLRTLG
YSQQQQEKIKPKVRSTVAQHHEALVGHGFTHAHIVALSQHPAALGTVAVKYQDMIAALPEA
THEAIVGVGKQWSGARALEALLTVAGELRGPPLQLDTGQLLKIAKRGGVTAVEAVHAWRN
ALTGAPLNLTPQQVVAIASNIGGKQALETVQRLLPVLCQAHGLTPDQVVAIASNNGGKQALE
TVQRLLPVLCQAHGLTPEQVVAIASNIGGKQALETVQRLLPVLCQAHGLTPDQVVAIASNIG
GKQALETVQRLLPVLCQAHGLTPDQVVAIASNGGGKQALETVQRLLPVLCQAHGLTPEQVV
AIASNIGGKQALETVQRLLPVLCQAHGLTPDQVVAIASNGGGKQALETVQRLLPVLCQAHGL
TPEQVVAIASNIGGKQALETVQRLLPVLCQAHGLTPDQVVAIASNGGGKQALETVQRLLPVL
CQAHGLTPEQVVAIASNIGGKQALETVQRLLPVLCQAHGLTPDQVVAIASNIGGKQALETVQ
RLLPVLCQAHGLTPDQVVAIASNGGGKQALETVQRLLPVLCQAHGLTPEQVVAIASNGGGK
QALETVQRLLPVLCQAHGLTPEQVVAIASNIGGKQALETVQRLLPVLCQAHGLTPDQVVAIA
SHDGGKQALETVQRLLPVLCQAHGLTPAQVVAIASNIGGRPALESIVAQLSRPDPALAALTN
DHLVALACLGGRPALDAVKKGLGGSGSYALGPYQISAPQLPAYNGQTVGTFYYVNDAGGL
ESKVFSSGGSGGSTNLSDIIEKETGKQLVIQESILMLPEEVEEVIGNKPESDILVHTAYDESTD
ENVMLLTSDAPEYKPWALVIQDSNGENKIKML*

- **DdCBE (L) – Nd5-L2 - G1333-N:**

MASVLTPLLLRGLTGSARRLPVPRAKIHSLDYKDHDGDYKDHDIDYKDDDDKMDIADLRTLG
YSQQQQEKIKPKVRSTVAQHHEALVGHGFTHAHIVALSQHPAALGTVAVKYQDMIAALPEA
THEAIVGVGKQWSGARALEALLTVAGELRGPPLQLDTGQLLKIAKRGGVTAVEAVHAWRN
ALTGAPLNLTPQQVVAIASNGGGKQALETVQRLLPVLCQAHGLTPEQVVAIASNIGGKQALE
TVQRLLPVLCQAHGLTPDQVVAIASNGGGKQALETVQRLLPVLCQAHGLTPEQVVAIASNIG
GKQALETVQRLLPVLCQAHGLTPDQVVAIASNGGGKQALETVQRLLPVLCQAHGLTPEQVV
AIASNIGGKQALETVQRLLPVLCQAHGLTPDQVVAIASNIGGKQALETVQRLLPVLCQAHGL
TPDQVVAIASNGGGKQALETVQRLLPVLCQAHGLTPEQVVAIASNGGGKQALETVQRLLPV
LCQAHGLTPEQVVAIASNIGGKQALETVQRLLPVLCQAHGLTPDQVVAIASHDGGKQALET
VQRLLPVLCQAHGLTPAQVVAIASNIGGKQALETVQRLLPVLCQAHGLTPDQVVAIASNIGG
KQALETVQRLLPVLCQAHGLTPDQVVAIASHDGGKQALETVQRLLPVLCQAHGLTPAQVVA
IASHDGGRPALESIVAQLSRPDPALAALTNDHLVALACLGGRPALDAVKKGLGGSGSYALG
PYQISAPQLPAYNGQTVGTFYYVNDAGGLESKVFSSGGSGGSTNLSDIIEKETGKQLVIQES
ILMLPEEVEEVIGNKPESDILVHTAYDESTDENVMLLTSDAPEYKPWALVIQDSNGENKIKML
*

- **DdCBE (L) – Nd6-L1 - G1333-N:**

MASVLTPLLLRGLTGSARRLPVPRAKIHSLDYKDHDGDYKDHDIDYKDDDDKMDIADLRTLG
YSQQQQEKIKPKVRSTVAQHHEALVGHGFTHAHIVALSQHPAALGTVAVKYQDMIAALPEA
THEAIVGVGKQWSGARALEALLTVAGELRGPPLQLDTGQLLKIAKRGGVTAVEAVHAWRN
ALTGAPLNLTPQQVVAIASNIGGKQALETVQRLLPVLCQAHGLTPDQVVAIASHDGGKQALE
TVQRLLPVLCQAHGLTPAQVVAIASNGGGKQALETVQRLLPVLCQAHGLTPEQVVAIASNIG
GKQALETVQRLLPVLCQAHGLTPDQVVAIASNIGGKQALETVQRLLPVLCQAHGLTPDQVV
AIASNIGGKQALETVQRLLPVLCQAHGLTPDQVVAIASNIGGKQALETVQRLLPVLCQAHGL
TPDQVVAIASNIGGKQALETVQRLLPVLCQAHGLTPDQVVAIASNIGGKQALETVQRLLPVL
CQAHGLTPDQVVAIASHDGGKQALETVQRLLPVLCQAHGLTPAQVVAIASHDGGKQALETV
QRLLPVLCQAHGLTPAQVVAIASHDGGKQALETVQRLLPVLCQAHGLTPAQVVAIASNIGG
RPALESIVAQLSRPDPALAALTNDHLVALACLGGRPALDAVKKGLGGSGSYALGPYQISAP
QLPAYNGQTVGTFYYVNDAGGLESKVFSSGGSGGSTNLSDIIEKETGKQLVIQESILMLPEE
VEEVIGNKPESDILVHTAYDESTDENVMLLTSDAPEYKPWALVIQDSNGENKIKML*

- **DdCBE (L) – Nd6-L2 - G1333-N:**

MASVLTPLLLRGLTGSARRLPVPRAKIHSLDYKDHDGDYKDHDIDYKDDDDKMDIADLRTLG
YSQQQQEKIKPKVRSTVAQHHEALVGHGFTHAHIVALSQHPAALGTVAVKYQDMIAALPEA
THEAIVGVGKRGAGARALEALLTVAGELRGPPLQLDTGQLLKIAKRGGVTAVEAVHAWRNA
LTGAPLNLTPQQVVAIASHDGGKQALETVQRLLPVLCQAHGLTPAQVVAIASNIGGKQALET
VQRLLPVLCQAHGLTPDQVVAIASNIGGKQALETVQRLLPVLCQAHGLTPDQVVAIASNIGG
KQALETVQRLLPVLCQAHGLTPDQVVAIASHDGGKQALETVQRLLPVLCQAHGLTPAQVVA
IASNGGGKQALETVQRLLPVLCQAHGLTPEQVVAIASNIGGKQALETVQRLLPVLCQAHGLT
PDQVVAIASNIGGKQALETVQRLLPVLCQAHGLTPDQVVAIASNIGGKQALETVQRLLPVLC
QAHGLTPDQVVAIASNIGGKQALETVQRLLPVLCQAHGLTPDQVVAIASNIGGKQALETVQR
LLPVLCQAHGLTPDQVVAIASNIGGKQALETVQRLLPVLCQAHGLTPDQVVAIASHDGGKQ
ALETVQRLLPVLCQAHGLTPAQVVAIASHDGGKQALETVQRLLPVLCQAHGLTPAQVVAIAS
HDGGRPALESIVAQLSRPDPALAALTNDHLVALACLGGRPALDAVKKGLGGSGSYALGPYQ
ISAPQLPAYNGQTVGTFYYVNDAGGLESKVFSSGGSGGSTNLSDIIEKETGKQLVIQESILML
PEEVEEVIGNKPESDILVHTAYDESTDENVMLLTSDAPEYKPWALVIQDSNGENKIKML*

- **DdCBE (L) – Cytb-L1 - G1333-N:**

MASVLTPLLLRGLTGSARRLPVPRAKIHSLDYKDHDGDYKDHDIDYKDDDDKMDIADLRTLG
YSQQQQEKIKPKVRSTVAQHHEALVGHGFTHAHIVALSQHPAALGTVAVKYQDMIAALPEA
THEAIVGVGKQWSGARALEALLTVAGELRGPPLQLDTGQLLKIAKRGGVTAVEAVHAWRN
ALTGAPLNLTPQQVVAIASNGGGKQALETVQRLLPVLCQAHGLTPEQVVAIASNGGGKQAL
ETVQRLLPVLCQAHGLTPEQVVAIASNNGGKQALETVQRLLPVLCQAHGLTPEQVVAIASN
GGGKQALETVQRLLPVLCQAHGLTPEQVVAIASHDGGKQALETVQRLLPVLCQAHGLTPA
QVVAIASNNGGKQALETVQRLLPVLCQAHGLTPEQVVAIASNIGGKQALETVQRLLPVLCQA
HGLTPDQVVAIASNNGGKQALETVQRLLPVLCQAHGLTPEQVVAIASNIGGKQALETVQRLL
PVLCQAHGLTPDQVVAIASHDGGKQALETVQRLLPVLCQAHGLTPAQVVAIASNNGGKQAL
ETVQRLLPVLCQAHGLTPEQVVAIASNGGGKQALETVQRLLPVLCQAHGLTPEQVVAIASNI
GGKQALETVQRLLPVLCQAHGLTPDQVVAIASNIGGKQALETVQRLLPVLCQAHGLTPDQV
VAIASNIGGRPALESIVAQLSRPDPALAALTNDHLVALACLGGRPALDAVKKGLGGSGSYAL
GPYQISAPQLPAYNGQTVGTFYYVNDAGGLESKVFSSGGSGGSTNLSDIIEKETGKQLVIQE
SILMLPEEVEEVIGNKPESDILVHTAYDESTDENVMLLTSDAPEYKPWALVIQDSNGENKIKM
L*

- **DdCBE (L) – Cytb-L2 - G1333-N:**

MASVLTPLLLRGLTGSARRLPVPRAKIHSLDYKDHDGDYKDHDIDYKDDDDKMDIADLRTLG
YSQQQQEKIKPKVRSTVAQHHEALVGHGFTHAHIVALSQHPAALGTVAVKYQDMIAALPEA
THEAIVGVGKQWSGARALEALLTVAGELRGPPLQLDTGQLLKIAKRGGVTAVEAVHAWRN
ALTGAPLNLTPQQVVAIASHDGGKQALETVQRLLPVLCQAHGLTPAQVVAIASNNGGKQAL
ETVQRLLPVLCQAHGLTPEQVVAIASNIGGKQALETVQRLLPVLCQAHGLTPDQVVAIASNN
GGKQALETVQRLLPVLCQAHGLTPEQVVAIASNIGGKQALETVQRLLPVLCQAHGLTPDQV
VAIASHDGGKQALETVQRLLPVLCQAHGLTPAQVVAIASNNGGKQALETVQRLLPVLCQAH
GLTPEQVVAIASNGGGKQALETVQRLLPVLCQAHGLTPEQVVAIASNIGGKQALETVQRLLP
VLCQAHGLTPDQVVAIASNIGGKQALETVQRLLPVLCQAHGLTPDQVVAIASNIGGKQALET
VQRLLPVLCQAHGLTPDQVVAIASNGGGKQALETVQRLLPVLCQAHGLTPEQVVAIASNGG
GRPALESIVAQLSRPDPALAALTNDHLVALACLGGRPALDAVKKGLGGSGSYALGPYQISA
PQLPAYNGQTVGTFYYVNDAGGLESKVFSSGGSGGSTNLSDIIEKETGKQLVIQESILMLPE
EVEEVIGNKPESDILVHTAYDESTDENVMLLTSDAPEYKPWALVIQDSNGENKIKML*

- **DdCBE (L) – Col-L1 - G1333-N:**

MASVLTPLLLRGLTGSARRLPVPRAKIHSLDYKDHDGDYKDHDIDYKDDDDKMDIADLRTLG
YSQQQQEKIKPKVRSTVAQHHEALVGHGFTHAHIVALSQHPAALGTVAVKYQDMIAALPEA
THEAIVGVGKQWSGARALEALLTVAGELRGPPLQLDTGQLLKIAKRGGVTAVEAVHAWRN
ALTGAPLNLTPQQVVAIASNGGGKQALETVQRLLPVLCQAHGLTPEQVVAIASNGGGKQAL
ETVQRLLPVLCQAHGLTPEQVVAIASNGGGKQALETVQRLLPVLCQAHGLTPEQVVAIASNI
GGKQALETVQRLLPVLCQAHGLTPDQVVAIASHDGGKQALETVQRLLPVLCQAHGLTPAQ
VVAIASHDGGKQALETVQRLLPVLCQAHGLTPAQVVAIASNGGGKQALETVQRLLPVLCQA
HGLTPEQVVAIASNIGGKQALETVQRLLPVLCQAHGLTPDQVVAIASNGGGKQALETVQRLL
PVLCQAHGLTPEQVVAIASNNGGKQALETVQRLLPVLCQAHGLTPEQVVAIASNGGGKQAL
ETVQRLLPVLCQAHGLTPEQVVAIASNGGGKQALETVQRLLPVLCQAHGLTPEQVVAIASH
DGGKQALETVQRLLPVLCQAHGLTPAQVVAIASNIGGKQALETVQRLLPVLCQAHGLTPDQ
VVAIASNGGGRPALESIVAQLSRPDPALAALTNDHLVALACLGGRPALDAVKKGLGGSGSY
ALGPYQISAPQLPAYNGQTVGTFYYVNDAGGLESKVFSSGGSGGSTNLSDIIEKETGKQLVI
QESILMLPEEVEEVIGNKPESDILVHTAYDESTDENVMLLTSDAPEYKPWALVIQDSNGENKI
KML*

- **DdCBE (L) – Col-L2 - G1333-N:**

MASVLTPLLLRGLTGSARRLPVPRAKIHSLDYKDHDGDYKDHDIDYKDDDDKMDIADLRTLG
YSQQQQEKIKPKVRSTVAQHHEALVGHGFTHAHIVALSQHPAALGTVAVKYQDMIAALPEA
THEAIVGVGKQWSGARALEALLTVAGELRGPPLQLDTGQLLKIAKRGGVTAVEAVHAWRN
ALTGAPLNLTPQQVVAIASNIGGKQALETVQRLLPVLCQAHGLTPDQVVAIASHDGGKQALE
TVQRLLPVLCQAHGLTPAQVVAIASHDGGKQALETVQRLLPVLCQAHGLTPAQVVAIASNG
GGKQALETVQRLLPVLCQAHGLTPEQVVAIASNIGGKQALETVQRLLPVLCQAHGLTPDQV
VAIASNGGGKQALETVQRLLPVLCQAHGLTPEQVVAIASNNGGKQALETVQRLLPVLCQAH
GLTPEQVVAIASNGGGKQALETVQRLLPVLCQAHGLTPEQVVAIASNGGGKQALETVQRLL
PVLCQAHGLTPEQVVAIASHDGGKQALETVQRLLPVLCQAHGLTPAQVVAIASNIGGKQAL
ETVQRLLPVLCQAHGLTPDQVVAIASNGGGKQALETVQRLLPVLCQAHGLTPEQVVAIASN
GGGRPALESIVAQLSRPDPALAALTNDHLVALACLGGRPALDAVKKGLGGSGSYALGPYQI
SAPQLPAYNGQTVGTFYYVNDAGGLESKVFSSGGSGGSTNLSDIIEKETGKQLVIQESILML
PEEVEEVIGNKPESDILVHTAYDESTDENVMLLTSDAPEYKPWALVIQDSNGENKIKML*

- **DdCBE (L) – ColI-L1 - G1333-N:**

MASVLTPLLLRGLTGSARRLPVPRAKIHSLDYKDHDGDYKDHDIDYKDDDDKMDIADLRTLG
YSQQQQEKIKPKVRSTVAQHHEALVGHGFTHAHIVALSQHPAALGTVAVKYQDMIAALPEA
THEAIVGVGKQWSGARALEALLTVAGELRGPPLQLDTGQLLKIAKRGGVTAVEAVHAWRN
ALTGAPLNLTPQQVVAIASNIGGKQALETVQRLLPVLCQAHGLTPDQVVAIASNIGGKQALE
TVQRLLPVLCQAHGLTPDQVVAIASHDGGKQALETVQRLLPVLCQAHGLTPAQVVAIASHD
GGKQALETVQRLLPVLCQAHGLTPAQVVAIASNNGGKQALETVQRLLPVLCQAHGLTPEQV
VAIASNGGGKQALETVQRLLPVLCQAHGLTPEQVVAIASNGGGKQALETVQRLLPVLCQAH
GLTPEQVVAIASNIGGKQALETVQRLLPVLCQAHGLTPDQVVAIASNIGGKQALETVQRLLP
VLCQAHGLTPDQVVAIASNIGGKQALETVQRLLPVLCQAHGLTPDQVVAIASNIGGKQALET
VQRLLPVLCQAHGLTPDQVVAIASHDGGKQALETVQRLLPVLCQAHGLTPAQVVAIASHDG
GKQALETVQRLLPVLCQAHGLTPAQVVAIASNIGGKQALETVQRLLPVLCQAHGLTPDQVV
AIASNGGGKQALETVQRLLPVLCQAHGLTPEQVVAIASNIGGRPALESIVAQLSRPDPALAA
LTNDHLVALACLGGRPALDAVKKGLGGSGSYALGPYQISAPQLPAYNGQTVGTFYYVNDA
GGLESKVFSSGGSGGSTNLSDIIEKETGKQLVIQESILMLPEEVEEVIGNKPESDILVHTAYD
ESTDENVMLLTSDAPEYKPWALVIQDSNGENKIKML*

- **DdCBE (L) – CoII-L2 - G1333-N:**

MASVLTPLLLRGLTGSARRLPVPRAKIHSLDYKDHDGDYKDHDIDYKDDDDKMDIADLRTLG
YSQQQQEKIKPKVRSTVAQHHEALVGHGFTHAHIVALSQHPAALGTVAVKYQDMIAALPEA
THEAIVGVGKQWSGARALEALLTVAGELRGPPLQLDTGQLLKIAKRGGVTAVEAVHAWRN
ALTGAPLNLTPQQVVAIASNGGGKQALETVQRLLPVLCQAHGLTPEQVVAIASNIGGKQALE
TVQRLLPVLCQAHGLTPDQVVAIASNIGGKQALETVQRLLPVLCQAHGLTPDQVVAIASNIG
GKQALETVQRLLPVLCQAHGLTPDQVVAIASNIGGKQALETVQRLLPVLCQAHGLTPDQVV
AIASHDGGKQALETVQRLLPVLCQAHGLTPAQVVAIASHDGGKQALETVQRLLPVLCQAHG
LTPAQVVAIASNIGGKQALETVQRLLPVLCQAHGLTPDQVVAIASNGGGKQALETVQRLLPV
LCQAHGLTPEQVVAIASNIGGKQALETVQRLLPVLCQAHGLTPDQVVAIASNNGGKQALET
VQRLLPVLCQAHGLTPEQVVAIASNNGGKQALETVQRLLPVLCQAHGLTPEQVVAIASNNG
GRPALESIVAQLSRPDPALAALTNDHLVALACLGGRPALDAVKKGLGGSGSYALGPYQISA
PQLPAYNGQTVGTFYYVNDAGGLESKVFSSGGSGGSTNLSDIIEKETGKQLVIQESILMLPE
EVEEVIGNKPESDILVHTAYDESTDENVMLLTSDAPEYKPWALVIQDSNGENKIKML*

- **DdCBE (L) – CoIII-L1 - G1333-N:**

MASVLTPLLLRGLTGSARRLPVPRAKIHSLDYKDHDGDYKDHDIDYKDDDDKMDIADLRTLG
YSQQQQEKIKPKVRSTVAQHHEALVGHGFTHAHIVALSQHPAALGTVAVKYQDMIAALPEA
THEAIVGVGKQWSGARALEALLTVAGELRGPPLQLDTGQLLKIAKRGGVTAVEAVHAWRN
ALTGAPLNLTPQQVVAIASHDGGKQALETVQRLLPVLCQAHGLTPAQVVAIASNGGGKQAL
ETVQRLLPVLCQAHGLTPEQVVAIASNIGGKQALETVQRLLPVLCQAHGLTPDQVVAIASNN
GGKQALETVQRLLPVLCQAHGLTPEQVVAIASHDGGKQALETVQRLLPVLCQAHGLTPAQV
VAIASNIGGKQALETVQRLLPVLCQAHGLTPDQVVAIASNGGGKQALETVQRLLPVLCQAH
GLTPEQVVAIASHDGGKQALETVQRLLPVLCQAHGLTPAQVVAIASNIGGKQALETVQRLLP
VLCQAHGLTPDQVVAIASNNGGKQALETVQRLLPVLCQAHGLTPEQVVAIASNNGGKQALE
TVQRLLPVLCQAHGLTPEQVVAIASNGGGKQALETVQRLLPVLCQAHGLTPEQVVAIASNN
GGKQALETVQRLLPVLCQAHGLTPEQVVAIASNGGGKQALETVQRLLPVLCQAHGLTPEQ
VVAIASNGGGKQALETVQRLLPVLCQAHGLTPEQVVAIASNGGGRPALESIVAQLSRPDPAL
AALTNDHLVALACLGGRPALDAVKKGLGGSGSYALGPYQISAPQLPAYNGQTVGTFYYVN
DAGGLESKVFSSGGSGGSTNLSDIIEKETGKQLVIQESILMLPEEVEEVIGNKPESDILVHTA
YDESTDENVMLLTSDAPEYKPWALVIQDSNGENKIKML*

- **DdCBE (L) – CoIII-L2 - G1333-N:**

MASVLTPLLLRGLTGSARRLPVPRAKIHSLDYKDHDGDYKDHDIDYKDDDDKMDIADLRTLG
YSQQQQEKIKPKVRSTVAQHHEALVGHGFTHAHIVALSQHPAALGTVAVKYQDMIAALPEA
THEAIVGVGKQWSGARALEALLTVAGELRGPPLQLDTGQLLKIAKRGGVTAVEAVHAWRN
ALTGAPLNLTPQQVVAIASNIGGKQALETVQRLLPVLCQAHGLTPDQVVAIASNNGGKQALE
TVQRLLPVLCQAHGLTPEQVVAIASHDGGKQALETVQRLLPVLCQAHGLTPAQVVAIASNIG
GKQALETVQRLLPVLCQAHGLTPDQVVAIASNGGGKQALETVQRLLPVLCQAHGLTPEQVV
AIASHDGGKQALETVQRLLPVLCQAHGLTPAQVVAIASNIGGKQALETVQRLLPVLCQAHGL
TPDQVVAIASNNGGKQALETVQRLLPVLCQAHGLTPEQVVAIASNNGGKQALETVQRLLPV
LCQAHGLTPEQVVAIASNGGGKQALETVQRLLPVLCQAHGLTPEQVVAIASNNGGKQALET
VQRLLPVLCQAHGLTPEQVVAIASNGGGKQALETVQRLLPVLCQAHGLTPEQVVAIASNGG
GRPALESIVAQLSRPDPALAALTNDHLVALACLGGRPALDAVKKGLGGSGSYALGPYQISA
PQLPAYNGQTVGTFYYVNDAGGLESKVFSSGGSGGSTNLSDIIEKETGKQLVIQESILMLPE
EVEEVIGNKPESDILVHTAYDESTDENVMLLTSDAPEYKPWALVIQDSNGENKIKML*

- **DdCBE (L) – Atp6-L1 - G1333-N:**

MASVLTPLLLRGLTGSARRLPVPRAKIHSLDYKDHDGDYKDHDIDYKDDDDKMDIADLRTLG
YSQQQQEKIKPKVRSTVAQHHEALVGHGFTHAHIVALSQHPAALGTVAVKYQDMIAALPEA

THEAIVGVGKRGAGARALEALLTVAGELRGPPLQLDTGQLLKIAKRGGVTAVEAVHAWRNA
LTGAPLNLTPQQVVAIASNIGGKQALETVQRLLPVLCQAHGLTPDQVVAIASHDGGKQALET
VQRLLPVLCQAHGLTPAQVVAIASHDGGKQALETVQRLLPVLCQAHGLTPAQVVAIASNNG
GKQALETVQRLLPVLCQAHGLTPEQVVAIASNGGGKQALETVQRLLPVLCQAHGLTPEQVV
AIASHDGGKQALETVQRLLPVLCQAHGLTPAQVVAIASNGGGKQALETVQRLLPVLCQAHG
LTPEQVVAIASHDGGKQALETVQRLLPVLCQAHGLTPAQVVAIASHDGGKQALETVQRLLP
VLCQAHGLTPAQVVAIASNIGGKQALETVQRLLPVLCQAHGLTPDQVVAIASNGGGKQALE
TVQRLLPVLCQAHGLTPEQVVAIASNGGGKQALETVQRLLPVLCQAHGLTPEQVVAIASHD
GGKQALETVQRLLPVLCQAHGLTPAQVVAIASNGGGKQALETVQRLLPVLCQAHGLTPEQ
VVAIASNGGGKQALETVQRLLPVLCQAHGLTPEQVVAIASNGGGRPALESIVAQLSRPDPAL
AALTNDHLVALACLGGRPALDAVKKGLGGSGSYALGPYQISAPQLPAYNGQTVGTFYYVN
DAGGLESKVFSSGGSGGSTNLSDIIEKETGKQLVIQESILMLPEEVEEVIGNKPESDILVHTA
YDESTDENVMLLTSDAPEYKPWALVIQDSNGENKIKML*

- **DdCBE (L) – Atp6-L2 - G1333-N:**

MASVLTPLLLRGLTGSARRLPVPRAKIHSLDYKDHDGDYKDHDIDYKDDDDKMDIADLRTLG
YSQQQQEKIKPKVRSTVAQHHEALVGHGFTHAHIVALSQHPAALGTVAVKYQDMIAALPEA
THEAIVGVGKQWSGARALEALLTVAGELRGPPLQLDTGQLLKIAKRGGVTAVEAVHAWRN
ALTGAPLNLTPQQVVAIASHDGGKQALETVQRLLPVLCQAHGLTPAQVVAIASNGGGKQAL
ETVQRLLPVLCQAHGLTPEQVVAIASHDGGKQALETVQRLLPVLCQAHGLTPAQVVAIASH
DGGKQALETVQRLLPVLCQAHGLTPAQVVAIASNIGGKQALETVQRLLPVLCQAHGLTPDQ
VVAIASNGGGKQALETVQRLLPVLCQAHGLTPEQVVAIASNGGGKQALETVQRLLPVLCQA
HGLTPEQVVAIASHDGGKQALETVQRLLPVLCQAHGLTPAQVVAIASNGGGKQALETVQRL
LPVLCQAHGLTPEQVVAIASNGGGKQALETVQRLLPVLCQAHGLTPEQVVAIASNGGGKQA
LETVQRLLPVLCQAHGLTPEQVVAIASHDGGKQALETVQRLLPVLCQAHGLTPAQVVAIASH
DGGRPALESIVAQLSRPDPALAALTNDHLVALACLGGRPALDAVKKGLGGSGSYALGPYQI
SAPQLPAYNGQTVGTFYYVNDAGGLESKVFSSGGSGGSTNLSDIIEKETGKQLVIQESILML
PEEVEEVIGNKPESDILVHTAYDESTDENVMLLTSDAPEYKPWALVIQDSNGENKIKML*

- **DdCBE (L) – Atp8-L1 - G1333-N:**

MASVLTPLLLRGLTGSARRLPVPRAKIHSLDYKDHDGDYKDHDIDYKDDDDKMDIADLRTLG
YSQQQQEKIKPKVRSTVAQHHEALVGHGFTHAHIVALSQHPAALGTVAVKYQDMIAALPEA
THEAIVGVGKQWSGARALEALLTVAGELRGPPLQLDTGQLLKIAKRGGVTAVEAVHAWRN
ALTGAPLNLTPQQVVAIASNNGGKQALETVQRLLPVLCQAHGLTPEQVVAIASHDGGKQAL
ETVQRLLPVLCQAHGLTPAQVVAIASHDGGKQALETVQRLLPVLCQAHGLTPAQVVAIASNI
GGKQALETVQRLLPVLCQAHGLTPDQVVAIASHDGGKQALETVQRLLPVLCQAHGLTPAQ
VVAIASNIGGKQALETVQRLLPVLCQAHGLTPDQVVAIASNIGGKQALETVQRLLPVLCQAH
GLTPDQVVAIASHDGGKQALETVQRLLPVLCQAHGLTPAQVVAIASNGGGKQALETVQRLL
PVLCQAHGLTPEQVVAIASNIGGKQALETVQRLLPVLCQAHGLTPDQVVAIASNNGGKQAL
ETVQRLLPVLCQAHGLTPEQVVAIASNIGGKQALETVQRLLPVLCQAHGLTPDQVVAIASNG
GGKQALETVQRLLPVLCQAHGLTPEQVVAIASNIGGKQALETVQRLLPVLCQAHGLTPDQV
VAIASHDGGRPALESIVAQLSRPDPALAALTNDHLVALACLGGRPALDAVKKGLGGSGSYA
LGPYQISAPQLPAYNGQTVGTFYYVNDAGGLESKVFSSGGSGGSTNLSDIIEKETGKQLVIQ
ESILMLPEEVEEVIGNKPESDILVHTAYDESTDENVMLLTSDAPEYKPWALVIQDSNGENKIK
ML*

- **DdCBE (L) – Atp8-L2 - G1333-N:**

MASVLTPLLLRGLTGSARRLPVPRAKIHSLDYKDHDGDYKDHDIDYKDDDDKMDIADLRTLG
YSQQQQEKIKPKVRSTVAQHHEALVGHGFTHAHIVALSQHPAALGTVAVKYQDMIAALPEA
THEAIVGVGKRGAGARALEALLTVAGELRGPPLQLDTGQLLKIAKRGGVTAVEAVHAWRNA

LTGAPLNLTPQQVVAIASHDGGKQALETVQRLLPVLCQAHGLTPAQVVAIASHDGGKQALE
TVQRLLPVLCQAHGLTPAQVVAIASNIGGKQALETVQRLLPVLCQAHGLTPDQVVAIASHDG
GKQALETVQRLLPVLCQAHGLTPAQVVAIASNIGGKQALETVQRLLPVLCQAHGLTPDQVV
AIASNIGGKQALETVQRLLPVLCQAHGLTPDQVVAIASHDGGKQALETVQRLLPVLCQAHGL
TPAQVVAIASNGGGKQALETVQRLLPVLCQAHGLTPEQVVAIASNIGGKQALETVQRLLPVL
CQAHGLTPDQVVAIASNNGGKQALETVQRLLPVLCQAHGLTPEQVVAIASNIGGKQALETV
QRLLPVLCQAHGLTPDQVVAIASNGGGKQALETVQRLLPVLCQAHGLTPEQVVAIASNIGG
KQALETVQRLLPVLCQAHGLTPDQVVAIASHDGGKQALETVQRLLPVLCQAHGLTPAQVVA
IASNIGGRPALESIVAQLSRPDPALAALTNDHLVALACLGGRPALDAVKKGLGGSGSYALGP
YQISAPQLPAYNGQTVGTFYYVNDAGGLESKVFSSGGSGGSTNLSDIIEKETGKQLVIQESI
LMLPEEVEEVIGNKPESDILVHTAYDESTDENVMLLTSDAPEYKPWALVIQDSNGENKIKML*

- **DdCBE (L) – Nd1-L1 - G1333-C:**

MASVLTPLLLRGLTGSARRLPVPRAKIHSLDYKDHDGDYKDHDIDYKDDDDKMDIADLRTLG
YSQQQQEKIKPKVRSTVAQHHEALVGHGFTHAHIVALSQHPAALGTVAVKYQDMIAALPEA
THEAIVGVGKQWSGARALEALLTVAGELRGPPLQLDTGQLLKIAKRGGVTAVEAVHAWRN
ALTGAPLNLTPQQVVAIASHDGGKQALETVQRLLPVLCQAHGLTPAQVVAIASNIGGKQALE
TVQRLLPVLCQAHGLTPDQVVAIASHDGGKQALETVQRLLPVLCQAHGLTPAQVVAIASNG
GGKQALETVQRLLPVLCQAHGLTPEQVVAIASHDGGKQALETVQRLLPVLCQAHGLTPAQV
VAIASNIGGKQALETVQRLLPVLCQAHGLTPDQVVAIASHDGGKQALETVQRLLPVLCQAH
GLTPAQVVAIASNIGGKQALETVQRLLPVLCQAHGLTPDQVVAIASHDGGKQALETVQRLLP
VLCQAHGLTPAQVVAIASNGGGKQALETVQRLLPVLCQAHGLTPEQVVAIASNIGGKQALE
TVQRLLPVLCQAHGLTPDQVVAIASNNGGKQALETVQRLLPVLCQAHGLTPEQVVAIASHD
GGKQALETVQRLLPVLCQAHGLTPAQVVAIASNIGGKQALETVQRLLPVLCQAHGLTPDQV
VAIASNGGGKQALETVQRLLPVLCQAHGLTPEQVVAIASNGGGRPALESIVAQLSRPDPALA
ALTNDHLVALACLGGRPALDAVKKGLGGSPTPYPNYANAGHVEGQSALFMRDNGISEGLV
FHNNPEGTCGFCVNMTETLLPENAKMTVVPPEGAIPVKRGATGETKVFTGNSNSPKSPTK
GGCSGGSTNLSDIIEKETGKQLVIQESILMLPEEVEEVIGNKPESDILVHTAYDESTDENVML
LTSDAPEYKPWALVIQDSNGENKIKML*

- **DdCBE (L) – Nd1-L2 - G1333-C:**

MASVLTPLLLRGLTGSARRLPVPRAKIHSLDYKDHDGDYKDHDIDYKDDDDKMDIADLRTLG
YSQQQQEKIKPKVRSTVAQHHEALVGHGFTHAHIVALSQHPAALGTVAVKYQDMIAALPEA
THEAIVGVGKQWSGARALEALLTVAGELRGPPLQLDTGQLLKIAKRGGVTAVEAVHAWRN
ALTGAPLNLTPQQVVAIASHDGGKQALETVQRLLPVLCQAHGLTPAQVVAIASNIGGKQALE
TVQRLLPVLCQAHGLTPDQVVAIASHDGGKQALETVQRLLPVLCQAHGLTPAQVVAIASNIG
GKQALETVQRLLPVLCQAHGLTPDQVVAIASHDGGKQALETVQRLLPVLCQAHGLTPAQVV
AIASNGGGKQALETVQRLLPVLCQAHGLTPEQVVAIASNIGGKQALETVQRLLPVLCQAHGL
TPDQVVAIASNNGGKQALETVQRLLPVLCQAHGLTPEQVVAIASHDGGKQALETVQRLLPV
LCQAHGLTPAQVVAIASNIGGKQALETVQRLLPVLCQAHGLTPDQVVAIASNGGGKQALET
VQRLLPVLCQAHGLTPEQVVAIASNGGGKQALETVQRLLPVLCQAHGLTPEQVVAIASNIG
GRPALESIVAQLSRPDPALAALTNDHLVALACLGGRPALDAVKKGLGGSPTPYPNYANAGH
VEGQSALFMRDNGISEGLVFHNNPEGTCGFCVNMTETLLPENAKMTVVPPEGAIPVKRGA
TGETKVFTGNSNSPKSPTKGGCSGGSTNLSDIIEKETGKQLVIQESILMLPEEVEEVIGNKPE
SDILVHTAYDESTDENVMLLTSDAPEYKPWALVIQDSNGENKIKML*

- **DdCBE (L) – Nd2-L1 - G1333-C:**

MASVLTPLLLRGLTGSARRLPVPRAKIHSLDYKDHDGDYKDHDIDYKDDDDKMDIADLRTLG
YSQQQQEKIKPKVRSTVAQHHEALVGHGFTHAHIVALSQHPAALGTVAVKYQDMIAALPEA

THEAIVGVGKQWSGARALEALLTVAGELRGPPLQLDTGQLLKIAKRGGVTAVEAVHAWRN
ALTGAPLNLTPQQVVAIASHDGGKQALETVQRLLPVLCQAHGLTPAQVVAIASHDGGKQAL
ETVQRLLPVLCQAHGLTPAQVVAIASNIGGKQALETVQRLLPVLCQAHGLTPDQVVAIASNN
GGKQALETVQRLLPVLCQAHGLTPEQVVAIASHDGGKQALETVQRLLPVLCQAHGLTPAQV
VAIASNIGGKQALETVQRLLPVLCQAHGLTPDQVVAIASHDGGKQALETVQRLLPVLCQAH
GLTPAQVVAIASHDGGKQALETVQRLLPVLCQAHGLTPAQVVAIASNIGGKQALETVQRLLP
VLCQAHGLTPDQVVAIASNIGGKQALETVQRLLPVLCQAHGLTPDQVVAIASHDGGKQALE
TVQRLLPVLCQAHGLTPAQVVAIASHDGGKQALETVQRLLPVLCQAHGLTPAQVVAIASNG
GGKQALETVQRLLPVLCQAHGLTPEQVVAIASNIGGKQALETVQRLLPVLCQAHGLTPDQV
VAIASNIGGKQALETVQRLLPVLCQAHGLTPDQVVAIASNGGGRPALESIVAQLSRPDPALA
ALTNDHLVALACLGGRPALDAVKKGLGGSPTPYPNYANAGHVEGQSALFMRDNGISEGLV
FHNNPEGTCGFCVNMTETLLPENAKMTVVPPEGAIPVKRGATGETKVFTGNSNSPKSPTK
GGCSGGSTNLSDIIEKETGKQLVIQESILMLPEEVEEVIGNKPESDILVHTAYDESTDENVML
LTSDAPEYKPWALVIQDSNGENKIKML*

- **DdCBE (L) – Nd2-L2 - G1333-C:**

MASVLTPLLLRGLTGSARRLPVPRAKIHSLDYKDHDGDYKDHDIDYKDDDDKMDIADLRTLG
YSQQQQEKIKPKVRSTVAQHHEALVGHGFTHAHIVALSQHPAALGTVAVKYQDMIAALPEA
THEAIVGVGKRGAGARALEALLTVAGELRGPPLQLDTGQLLKIAKRGGVTAVEAVHAWRNA
LTGAPLNLTPQQVVAIASNNGGKQALETVQRLLPVLCQAHGLTPEQVVAIASHDGGKQALE
TVQRLLPVLCQAHGLTPAQVVAIASNIGGKQALETVQRLLPVLCQAHGLTPDQVVAIASHDG
GKQALETVQRLLPVLCQAHGLTPAQVVAIASHDGGKQALETVQRLLPVLCQAHGLTPAQVV
AIASNIGGKQALETVQRLLPVLCQAHGLTPDQVVAIASNIGGKQALETVQRLLPVLCQAHGL
TPDQVVAIASHDGGKQALETVQRLLPVLCQAHGLTPAQVVAIASHDGGKQALETVQRLLPV
LCQAHGLTPAQVVAIASNGGGKQALETVQRLLPVLCQAHGLTPEQVVAIASNIGGKQALET
VQRLLPVLCQAHGLTPDQVVAIASNIGGKQALETVQRLLPVLCQAHGLTPDQVVAIASNGG
GKQALETVQRLLPVLCQAHGLTPEQVVAIASNIGGRPALESIVAQLSRPDPALAALTNDHLV
ALACLGGRPALDAVKKGLGGSPTPYPNYANAGHVEGQSALFMRDNGISEGLVFHNNPEGT
CGFCVNMTETLLPENAKMTVVPPEGAIPVKRGATGETKVFTGNSNSPKSPTKGGCSGGST
NLSDIIEKETGKQLVIQESILMLPEEVEEVIGNKPESDILVHTAYDESTDENVMLLTSDAPEYK
PWALVIQDSNGENKIKML*

- **DdCBE (L) – Nd3-L1 - G1333-C:**

MASVLTPLLLRGLTGSARRLPVPRAKIHSLDYKDHDGDYKDHDIDYKDDDDKMDIADLRTLG
YSQQQQEKIKPKVRSTVAQHHEALVGHGFTHAHIVALSQHPAALGTVAVKYQDMIAALPEA
THEAIVGVGKQWSGARALEALLTVAGELRGPPLQLDTGQLLKIAKRGGVTAVEAVHAWRN
ALTGAPLNLTPQQVVAIASHDGGKQALETVQRLLPVLCQAHGLTPAQVVAIASHDGGKQAL
ETVQRLLPVLCQAHGLTPAQVVAIASHDGGKQALETVQRLLPVLCQAHGLTPAQVVAIASN
GGGKQALETVQRLLPVLCQAHGLTPEQVVAIASNIGGKQALETVQRLLPVLCQAHGLTPDQ
VVAIASNIGGKQALETVQRLLPVLCQAHGLTPDQVVAIASHDGGKQALETVQRLLPVLCQAH
GLTPAQVVAIASNNGGKQALETVQRLLPVLCQAHGLTPEQVVAIASHDGGKQALETVQRLL
PVLCQAHGLTPAQVVAIASNGGGKQALETVQRLLPVLCQAHGLTPEQVVAIASNIGGKQAL
ETVQRLLPVLCQAHGLTPDQVVAIASNIGGKQALETVQRLLPVLCQAHGLTPDQVVAIASNG
GGKQALETVQRLLPVLCQAHGLTPEQVVAIASNGGGKQALETVQRLLPVLCQAHGLTPEQ
VVAIASHDGGKQALETVQRLLPVLCQAHGLTPAQVVAIASNGGGKQALETVQRLLPVLCQA
HGLTPEQVVAIASNIGGRPALESIVAQLSRPDPALAALTNDHLVALACLGGRPALDAVKKGL
GGSPTPYPNYANAGHVEGQSALFMRDNGISEGLVFHNNPEGTCGFCVNMTETLLPENAK
MTVVPPEGAIPVKRGATGETKVFTGNSNSPKSPTKGGCSGGSTNLSDIIEKETGKQLVIQES
ILMLPEEVEEVIGNKPESDILVHTAYDESTDENVMLLTSDAPEYKPWALVIQDSNGENKIKML
*

- **DdCBE (L) – Nd3-L2 - G1333-C:**

MASVLTPLLLRGLTGSARRLPVPRAKIHSLDYKDHDGDYKDHDIDYKDDDDKMDIADLRTLG
YSQQQQEKIKPKVRSTVAQHHEALVGHGFTHAHIVALSQHPAALGTVAVKYQDMIAALPEA
THEAIVGVGKQWSGARALEALLTVAGELRGPPLQLDTGQLLKIAKRGGVTAVEAVHAWRN
ALTGAPLNLTPQQVVAIASNIGGKQALETVQRLLPVLCQAHGLTPDQVVAIASNIGGKQALE
TVQRLLPVLCQAHGLTPDQVVAIASHDGGKQALETVQRLLPVLCQAHGLTPAQVVAIASNN
GGKQALETVQRLLPVLCQAHGLTPEQVVAIASHDGGKQALETVQRLLPVLCQAHGLTPAQV
VAIASNGGGKQALETVQRLLPVLCQAHGLTPEQVVAIASNIGGKQALETVQRLLPVLCQAH
GLTPDQVVAIASNIGGKQALETVQRLLPVLCQAHGLTPDQVVAIASNGGGKQALETVQRLLP
VLCQAHGLTPEQVVAIASNGGGKQALETVQRLLPVLCQAHGLTPEQVVAIASHDGGKQALE
TVQRLLPVLCQAHGLTPAQVVAIASNGGGKQALETVQRLLPVLCQAHGLTPEQVVAIASNIG
GKQALETVQRLLPVLCQAHGLTPDQVVAIASNNGGRPALESIVAQLSRPDPALAALTNDHLV
ALACLGGRPALDAVKKGLGGSPTPYPNYANAGHVEGQSALFMRDNGISEGLVFHNNPEGT
CGFCVNMTETLLPENAKMTVVPPEGAIPVKRGATGETKVFTGNSNSPKSPTKGGCSGGST
NLSDIIEKETGKQLVIQESILMLPEEVEEVIGNKPESDILVHTAYDESTDENVMLLTSDAPEYK
PWALVIQDSNGENKIKML*

- **DdCBE (L) – Nd4-L1 - G1333-C:**

MASVLTPLLLRGLTGSARRLPVPRAKIHSLDYKDHDGDYKDHDIDYKDDDDKMDIADLRTLG
YSQQQQEKIKPKVRSTVAQHHEALVGHGFTHAHIVALSQHPAALGTVAVKYQDMIAALPEA
THEAIVGVGKQWSGARALEALLTVAGELRGPPLQLDTGQLLKIAKRGGVTAVEAVHAWRN
ALTGAPLNLTPQQVVAIASHDGGKQALETVQRLLPVLCQAHGLTPAQVVAIASNIGGKQALE
TVQRLLPVLCQAHGLTPDQVVAIASHDGGKQALETVQRLLPVLCQAHGLTPAQVVAIASNG
GGKQALETVQRLLPVLCQAHGLTPEQVVAIASNIGGKQALETVQRLLPVLCQAHGLTPDQV
VAIASNIGGKQALETVQRLLPVLCQAHGLTPDQVVAIASNGGGKQALETVQRLLPVLCQAH
GLTPEQVVAIASNNGGKQALETVQRLLPVLCQAHGLTPEQVVAIASHDGGKQALETVQRLL
PVLCQAHGLTPAQVVAIASNGGGKQALETVQRLLPVLCQAHGLTPEQVVAIASNIGGKQAL
ETVQRLLPVLCQAHGLTPDQVVAIASHDGGKQALETVQRLLPVLCQAHGLTPAQVVAIASN
GGGKQALETVQRLLPVLCQAHGLTPEQVVAIASNIGGKQALETVQRLLPVLCQAHGLTPDQ
VVAIASHDGGKQALETVQRLLPVLCQAHGLTPAQVVAIASHDGGRPALESIVAQLSRPDPAL
AALTNDHLVALACLGGRPALDAVKKGLGGSPTPYPNYANAGHVEGQSALFMRDNGISEGL
VFHNNPEGTCGFCVNMTETLLPENAKMTVVPPEGAIPVKRGATGETKVFTGNSNSPKSPT
KGGCSGGSTNLSDIIEKETGKQLVIQESILMLPEEVEEVIGNKPESDILVHTAYDESTDENVM
LLTSDAPEYKPWALVIQDSNGENKIKML*

- **DdCBE (L) – Nd4-L2 - G1333-C:**

MASVLTPLLLRGLTGSARRLPVPRAKIHSLDYKDHDGDYKDHDIDYKDDDDKMDIADLRTLG
YSQQQQEKIKPKVRSTVAQHHEALVGHGFTHAHIVALSQHPAALGTVAVKYQDMIAALPEA
THEAIVGVGKQWSGARALEALLTVAGELRGPPLQLDTGQLLKIAKRGGVTAVEAVHAWRN
ALTGAPLNLTPQQVVAIASNIGGKQALETVQRLLPVLCQAHGLTPDQVVAIASNIGGKQALE
TVQRLLPVLCQAHGLTPDQVVAIASNGGGKQALETVQRLLPVLCQAHGLTPEQVVAIASNN
GGKQALETVQRLLPVLCQAHGLTPEQVVAIASHDGGKQALETVQRLLPVLCQAHGLTPAQV
VAIASNGGGKQALETVQRLLPVLCQAHGLTPEQVVAIASNIGGKQALETVQRLLPVLCQAH
GLTPDQVVAIASHDGGKQALETVQRLLPVLCQAHGLTPAQVVAIASNGGGKQALETVQRLL
PVLCQAHGLTPEQVVAIASNIGGKQALETVQRLLPVLCQAHGLTPDQVVAIASHDGGKQAL
ETVQRLLPVLCQAHGLTPAQVVAIASHDGGKQALETVQRLLPVLCQAHGLTPAQVVAIASNI
GGRPALESIVAQLSRPDPALAALTNDHLVALACLGGRPALDAVKKGLGGSPTPYPNYANAG
HVEGQSALFMRDNGISEGLVFHNNPEGTCGFCVNMTETLLPENAKMTVVPPEGAIPVKRG
ATGETKVFTGNSNSPKSPTKGGCSGGSTNLSDIIEKETGKQLVIQESILMLPEEVEEVIGNKP
ESDILVHTAYDESTDENVMLLTSDAPEYKPWALVIQDSNGENKIKML*

- **DdCBE (L) – Nd4l-L1 - G1333-C:**

MASVLTPLLLRGLTGSARRLPVPRAKIHSLDYKDHDGDYKDHDIDYKDDDDKMDIADLRTLG
YSQQQQEKIKPKVRSTVAQHHEALVGHGFTHAHIVALSQHPAALGTVAVKYQDMIAALPEA
THEAIVGVGKQWSGARALEALLTVAGELRGPPLQLDTGQLLKIAKRGGVTAVEAVHAWRN
ALTGAPLNLTPQQVVAIASHDGGKQALETVQRLLPVLCQAHGLTPAQVVAIASNIGGKQALE
TVQRLLPVLCQAHGLTPDQVVAIASNIGGKQALETVQRLLPVLCQAHGLTPDQVVAIASNIG
GKQALETVQRLLPVLCQAHGLTPDQVVAIASHDGGKQALETVQRLLPVLCQAHGLTPAQVV
AIASNIGGKQALETVQRLLPVLCQAHGLTPDQVVAIASHDGGKQALETVQRLLPVLCQAHGL
TPAQVVAIASNNGGKQALETVQRLLPVLCQAHGLTPEQVVAIASNGGGKQALETVQRLLPV
LCQAHGLTPEQVVAIASNIGGKQALETVQRLLPVLCQAHGLTPDQVVAIASHDGGKQALET
VQRLLPVLCQAHGLTPAQVVAIASNNGGKQALETVQRLLPVLCQAHGLTPEQVVAIASNNG
GKQALETVQRLLPVLCQAHGLTPEQVVAIASNIGGKQALETVQRLLPVLCQAHGLTPDQVV
AIASNIGGKQALETVQRLLPVLCQAHGLTPDQVVAIASHDGGRPALESIVAQLSRPDPALAA
LTNDHLVALACLGGRPALDAVKKGLGGSPTPYPNYANAGHVEGQSALFMRDNGISEGLVF
HNNPEGTCGFCVNMTETLLPENAKMTVVPPEGAIPVKRGATGETKVFTGNSNSPKSPTKG
GCSGGSTNLSDIIEKETGKQLVIQESILMLPEEVEEVIGNKPESDILVHTAYDESTDENVMLLT
SDAPEYKPWALVIQDSNGENKIKML*

- **DdCBE (L) – Nd4l-L2 - G1333-C:**

MASVLTPLLLRGLTGSARRLPVPRAKIHSLDYKDHDGDYKDHDIDYKDDDDKMDIADLRTLG
YSQQQQEKIKPKVRSTVAQHHEALVGHGFTHAHIVALSQHPAALGTVAVKYQDMIAALPEA
THEAIVGVGKRGAGARALEALLTVAGELRGPPLQLDTGQLLKIAKRGGVTAVEAVHAWRNA
LTGAPLNLTPQQVVAIASHDGGKQALETVQRLLPVLCQAHGLTPAQVVAIASNIGGKQALET
VQRLLPVLCQAHGLTPDQVVAIASHDGGKQALETVQRLLPVLCQAHGLTPAQVVAIASNNG
GKQALETVQRLLPVLCQAHGLTPEQVVAIASNGGGKQALETVQRLLPVLCQAHGLTPEQVV
AIASNIGGKQALETVQRLLPVLCQAHGLTPDQVVAIASHDGGKQALETVQRLLPVLCQAHGL
TPAQVVAIASNNGGKQALETVQRLLPVLCQAHGLTPEQVVAIASNNGGKQALETVQRLLPV
LCQAHGLTPEQVVAIASNIGGKQALETVQRLLPVLCQAHGLTPDQVVAIASNIGGKQALETV
QRLLPVLCQAHGLTPDQVVAIASHDGGKQALETVQRLLPVLCQAHGLTPAQVVAIASNIGG
KQALETVQRLLPVLCQAHGLTPDQVVAIASNNGGRPALESIVAQLSRPDPALAALTNDHLVA
LACLGGRPALDAVKKGLGGSPTPYPNYANAGHVEGQSALFMRDNGISEGLVFHNNPEGTC
GFCVNMTETLLPENAKMTVVPPEGAIPVKRGATGETKVFTGNSNSPKSPTKGGCSGGSTN
LSDIIEKETGKQLVIQESILMLPEEVEEVIGNKPESDILVHTAYDESTDENVMLLTSDAPEYKP
WALVIQDSNGENKIKML*

- **DdCBE (L) – Nd5-L1 - G1333-C:**

MASVLTPLLLRGLTGSARRLPVPRAKIHSLDYKDHDGDYKDHDIDYKDDDDKMDIADLRTLG
YSQQQQEKIKPKVRSTVAQHHEALVGHGFTHAHIVALSQHPAALGTVAVKYQDMIAALPEA
THEAIVGVGKQWSGARALEALLTVAGELRGPPLQLDTGQLLKIAKRGGVTAVEAVHAWRN
ALTGAPLNLTPQQVVAIASNIGGKQALETVQRLLPVLCQAHGLTPDQVVAIASNNGGKQALE
TVQRLLPVLCQAHGLTPEQVVAIASNIGGKQALETVQRLLPVLCQAHGLTPDQVVAIASNIG
GKQALETVQRLLPVLCQAHGLTPDQVVAIASNGGGKQALETVQRLLPVLCQAHGLTPEQVV
AIASNIGGKQALETVQRLLPVLCQAHGLTPDQVVAIASNGGGKQALETVQRLLPVLCQAHGL
TPEQVVAIASNIGGKQALETVQRLLPVLCQAHGLTPDQVVAIASNGGGKQALETVQRLLPVL
CQAHGLTPEQVVAIASNIGGKQALETVQRLLPVLCQAHGLTPDQVVAIASNIGGKQALETVQ
RLLPVLCQAHGLTPDQVVAIASNGGGKQALETVQRLLPVLCQAHGLTPEQVVAIASNGGGK
QALETVQRLLPVLCQAHGLTPEQVVAIASNIGGKQALETVQRLLPVLCQAHGLTPDQVVAIA
SHDGGKQALETVQRLLPVLCQAHGLTPAQVVAIASNIGGRPALESIVAQLSRPDPALAALTN
DHLVALACLGGRPALDAVKKGLGGSPTPYPNYANAGHVEGQSALFMRDNGISEGLVFHNN
PEGTCGFCVNMTETLLPENAKMTVVPPEGAIPVKRGATGETKVFTGNSNSPKSPTKGGCS

GGSTNLSDIIEKETGKQLVIQESILMLPEEVEEVIGNKPESDILVHTAYDESTDENVMLLTSDA
PEYKPWALVIQDSNGENKIKML*

- **DdCBE (L) – Nd5-L2 - G1333-C:**

MASVLTPLLLRGLTGSARRLPVPRAKIHSLDYKDHDGDYKDHDIDYKDDDDKMDIADLRTLG
YSQQQQEKIKPKVRSTVAQHHEALVGHGFTHAHIVALSQHPAALGTVAVKYQDMIAALPEA
THEAIVGVGKQWSGARALEALLTVAGELRGPPLQLDTGQLLKIAKRGGVTAVEAVHAWRN
ALTGAPLNLTPQQVVAIASNGGGKQALETVQRLLPVLCQAHGLTPEQVVAIASNIGGKQALE
TVQRLLPVLCQAHGLTPDQVVAIASNGGGKQALETVQRLLPVLCQAHGLTPEQVVAIASNIG
GKQALETVQRLLPVLCQAHGLTPDQVVAIASNGGGKQALETVQRLLPVLCQAHGLTPEQVV
AIASNIGGKQALETVQRLLPVLCQAHGLTPDQVVAIASNIGGKQALETVQRLLPVLCQAHGL
TPDQVVAIASNGGGKQALETVQRLLPVLCQAHGLTPEQVVAIASNGGGKQALETVQRLLPV
LCQAHGLTPEQVVAIASNIGGKQALETVQRLLPVLCQAHGLTPDQVVAIASHDGGKQALET
VQRLLPVLCQAHGLTPAQVVAIASNIGGKQALETVQRLLPVLCQAHGLTPDQVVAIASNIGG
KQALETVQRLLPVLCQAHGLTPDQVVAIASHDGGKQALETVQRLLPVLCQAHGLTPAQVVA
IASHDGGRPALESIVAQLSRPDPALAALTNDHLVALACLGGRPALDAVKKGLGGSPTPYPN
YANAGHVEGQSALFMRDNGISEGLVFHNNPEGTCGFCVNMTETLLPENAKMTVVPPEGAI
PVKRGATGETKVFTGNSNSPKSPTKGGCSGGSTNLSDIIEKETGKQLVIQESILMLPEEVEE
VIGNKPESDILVHTAYDESTDENVMLLTSDAPEYKPWALVIQDSNGENKIKML*

- **DdCBE (L) – Nd6-L1 - G1333-C:**

MASVLTPLLLRGLTGSARRLPVPRAKIHSLDYKDHDGDYKDHDIDYKDDDDKMDIADLRTLG
YSQQQQEKIKPKVRSTVAQHHEALVGHGFTHAHIVALSQHPAALGTVAVKYQDMIAALPEA
THEAIVGVGKQWSGARALEALLTVAGELRGPPLQLDTGQLLKIAKRGGVTAVEAVHAWRN
ALTGAPLNLTPQQVVAIASNIGGKQALETVQRLLPVLCQAHGLTPDQVVAIASHDGGKQALE
TVQRLLPVLCQAHGLTPAQVVAIASNGGGKQALETVQRLLPVLCQAHGLTPEQVVAIASNIG
GKQALETVQRLLPVLCQAHGLTPDQVVAIASNIGGKQALETVQRLLPVLCQAHGLTPDQVV
AIASNIGGKQALETVQRLLPVLCQAHGLTPDQVVAIASNIGGKQALETVQRLLPVLCQAHGL
TPDQVVAIASNIGGKQALETVQRLLPVLCQAHGLTPDQVVAIASNIGGKQALETVQRLLPVL
CQAHGLTPDQVVAIASHDGGKQALETVQRLLPVLCQAHGLTPAQVVAIASHDGGKQALETV
QRLLPVLCQAHGLTPAQVVAIASHDGGKQALETVQRLLPVLCQAHGLTPAQVVAIASNIGG
RPALESIVAQLSRPDPALAALTNDHLVALACLGGRPALDAVKKGLGGSPTPYPNYANAGHV
EGQSALFMRDNGISEGLVFHNNPEGTCGFCVNMTETLLPENAKMTVVPPEGAIPVKRGAT
GETKVFTGNSNSPKSPTKGGCSGGSTNLSDIIEKETGKQLVIQESILMLPEEVEEVIGNKPES
DILVHTAYDESTDENVMLLTSDAPEYKPWALVIQDSNGENKIKML*

- **DdCBE (L) – Nd6-L2 - G1333-C:**

MASVLTPLLLRGLTGSARRLPVPRAKIHSLDYKDHDGDYKDHDIDYKDDDDKMDIADLRTLG
YSQQQQEKIKPKVRSTVAQHHEALVGHGFTHAHIVALSQHPAALGTVAVKYQDMIAALPEA
THEAIVGVGKRGAGARALEALLTVAGELRGPPLQLDTGQLLKIAKRGGVTAVEAVHAWRNA
LTGAPLNLTPQQVVAIASHDGGKQALETVQRLLPVLCQAHGLTPAQVVAIASNIGGKQALET
VQRLLPVLCQAHGLTPDQVVAIASNIGGKQALETVQRLLPVLCQAHGLTPDQVVAIASNIGG
KQALETVQRLLPVLCQAHGLTPDQVVAIASHDGGKQALETVQRLLPVLCQAHGLTPAQVVA
IASNGGGKQALETVQRLLPVLCQAHGLTPEQVVAIASNIGGKQALETVQRLLPVLCQAHGLT
PDQVVAIASNIGGKQALETVQRLLPVLCQAHGLTPDQVVAIASNIGGKQALETVQRLLPVLC
QAHGLTPDQVVAIASNIGGKQALETVQRLLPVLCQAHGLTPDQVVAIASNIGGKQALETVQR
LLPVLCQAHGLTPDQVVAIASNIGGKQALETVQRLLPVLCQAHGLTPDQVVAIASHDGGKQ
ALETVQRLLPVLCQAHGLTPAQVVAIASHDGGKQALETVQRLLPVLCQAHGLTPAQVVAIAS
HDGGRPALESIVAQLSRPDPALAALTNDHLVALACLGGRPALDAVKKGLGGSPTPYPNYAN

AGHVEGQSALFMRDNGISEGLVFHNNPEGTCGFCVNMTETLLPENAKMTVVPPEGAIPVK
RGATGETKVFTGNSNSPKSPTKGGCSGGSTNLSDIIEKETGKQLVIQESILMLPEEVEEVIG
NKPESDILVHTAYDESTDENVMLLTSDAPEYKPWALVIQDSNGENKIKML*

- **DdCBE (L) – Cytb-L1 - G1333-C:**

MASVLTPLLLRGLTGSARRLPVPRAKIHSLDYKDHDGDYKDHDIDYKDDDDKMDIADLRTLG
YSQQQQEKIKPKVRSTVAQHHEALVGHGFTHAHIVALSQHPAALGTVAVKYQDMIAALPEA
THEAIVGVGKQWSGARALEALLTVAGELRGPPLQLDTGQLLKIAKRGGVTAVEAVHAWRN
ALTGAPLNLTPQQVVAIASNGGGKQALETVQRLLPVLCQAHGLTPEQVVAIASNGGGKQAL
ETVQRLLPVLCQAHGLTPEQVVAIASNNGGKQALETVQRLLPVLCQAHGLTPEQVVAIASN
GGGKQALETVQRLLPVLCQAHGLTPEQVVAIASHDGGKQALETVQRLLPVLCQAHGLTPA
QVVAIASNNGGKQALETVQRLLPVLCQAHGLTPEQVVAIASNIGGKQALETVQRLLPVLCQA
HGLTPDQVVAIASNNGGKQALETVQRLLPVLCQAHGLTPEQVVAIASNIGGKQALETVQRLL
PVLCQAHGLTPDQVVAIASHDGGKQALETVQRLLPVLCQAHGLTPAQVVAIASNNGGKQAL
ETVQRLLPVLCQAHGLTPEQVVAIASNGGGKQALETVQRLLPVLCQAHGLTPEQVVAIASNI
GGKQALETVQRLLPVLCQAHGLTPDQVVAIASNIGGKQALETVQRLLPVLCQAHGLTPDQV
VAIASNIGGRPALESIVAQLSRPDPALAALTNDHLVALACLGGRPALDAVKKGLGGSPTPYP
NYANAGHVEGQSALFMRDNGISEGLVFHNNPEGTCGFCVNMTETLLPENAKMTVVPPEGA
IPVKRGATGETKVFTGNSNSPKSPTKGGCSGGSTNLSDIIEKETGKQLVIQESILMLPEEVEE
VIGNKPESDILVHTAYDESTDENVMLLTSDAPEYKPWALVIQDSNGENKIKML*

- **DdCBE (L) – Cytb-L2 - G1333-C:**

MASVLTPLLLRGLTGSARRLPVPRAKIHSLDYKDHDGDYKDHDIDYKDDDDKMDIADLRTLG
YSQQQQEKIKPKVRSTVAQHHEALVGHGFTHAHIVALSQHPAALGTVAVKYQDMIAALPEA
THEAIVGVGKQWSGARALEALLTVAGELRGPPLQLDTGQLLKIAKRGGVTAVEAVHAWRN
ALTGAPLNLTPQQVVAIASHDGGKQALETVQRLLPVLCQAHGLTPAQVVAIASNNGGKQAL
ETVQRLLPVLCQAHGLTPEQVVAIASNIGGKQALETVQRLLPVLCQAHGLTPDQVVAIASNN
GGKQALETVQRLLPVLCQAHGLTPEQVVAIASNIGGKQALETVQRLLPVLCQAHGLTPDQV
VAIASHDGGKQALETVQRLLPVLCQAHGLTPAQVVAIASNNGGKQALETVQRLLPVLCQAH
GLTPEQVVAIASNGGGKQALETVQRLLPVLCQAHGLTPEQVVAIASNIGGKQALETVQRLLP
VLCQAHGLTPDQVVAIASNIGGKQALETVQRLLPVLCQAHGLTPDQVVAIASNIGGKQALET
VQRLLPVLCQAHGLTPDQVVAIASNGGGKQALETVQRLLPVLCQAHGLTPEQVVAIASNGG
GRPALESIVAQLSRPDPALAALTNDHLVALACLGGRPALDAVKKGLGGSPTPYPNYANAGH
VEGQSALFMRDNGISEGLVFHNNPEGTCGFCVNMTETLLPENAKMTVVPPEGAIPVKRGA
TGETKVFTGNSNSPKSPTKGGCSGGSTNLSDIIEKETGKQLVIQESILMLPEEVEEVIGNKPE
SDILVHTAYDESTDENVMLLTSDAPEYKPWALVIQDSNGENKIKML*

- **DdCBE (L) – Col-L1 - G1333-C:**

MASVLTPLLLRGLTGSARRLPVPRAKIHSLDYKDHDGDYKDHDIDYKDDDDKMDIADLRTLG
YSQQQQEKIKPKVRSTVAQHHEALVGHGFTHAHIVALSQHPAALGTVAVKYQDMIAALPEA
THEAIVGVGKQWSGARALEALLTVAGELRGPPLQLDTGQLLKIAKRGGVTAVEAVHAWRN
ALTGAPLNLTPQQVVAIASNGGGKQALETVQRLLPVLCQAHGLTPEQVVAIASNGGGKQAL
ETVQRLLPVLCQAHGLTPEQVVAIASNGGGKQALETVQRLLPVLCQAHGLTPEQVVAIASNI
GGKQALETVQRLLPVLCQAHGLTPDQVVAIASHDGGKQALETVQRLLPVLCQAHGLTPAQ
VVAIASHDGGKQALETVQRLLPVLCQAHGLTPAQVVAIASNGGGKQALETVQRLLPVLCQA
HGLTPEQVVAIASNIGGKQALETVQRLLPVLCQAHGLTPDQVVAIASNGGGKQALETVQRLL
PVLCQAHGLTPEQVVAIASNNGGKQALETVQRLLPVLCQAHGLTPEQVVAIASNGGGKQAL
ETVQRLLPVLCQAHGLTPEQVVAIASNGGGKQALETVQRLLPVLCQAHGLTPEQVVAIASH
DGGKQALETVQRLLPVLCQAHGLTPAQVVAIASNIGGKQALETVQRLLPVLCQAHGLTPDQ
VVAIASNGGGRPALESIVAQLSRPDPALAALTNDHLVALACLGGRPALDAVKKGLGGSPTP

YPNYANAGHVEGQSALFMRDNGISEGLVFHNNPEGTCGFCVNMTETLLPENAKMTVVPPE
GAIPVKRGATGETKVFTGNSNSPKSPTKGGCSGGSTNLSDIIEKETGKQLVIQESILMLPEEV
EEVIGNKPESDILVHTAYDESTDENVMLLTSDAPEYKPWALVIQDSNGENKIKML*

- **DdCBE (L) – CoI-L2 - G1333-C:**

MASVLTPLLLRGLTGSARRLPVPRAKIHSLDYKDHDGDYKDHDIDYKDDDDKMDIADLRTLG
YSQQQQEKIKPKVRSTVAQHHEALVGHGFTHAHIVALSQHPAALGTVAVKYQDMIAALPEA
THEAIVGVGKQWSGARALEALLTVAGELRGPPLQLDTGQLLKIAKRGGVTAVEAVHAWRN
ALTGAPLNLTPQQVVAIASNIGGKQALETVQRLLPVLCQAHGLTPDQVVAIASHDGGKQALE
TVQRLLPVLCQAHGLTPAQVVAIASHDGGKQALETVQRLLPVLCQAHGLTPAQVVAIASNG
GGKQALETVQRLLPVLCQAHGLTPEQVVAIASNIGGKQALETVQRLLPVLCQAHGLTPDQV
VAIASNGGGKQALETVQRLLPVLCQAHGLTPEQVVAIASNNGGKQALETVQRLLPVLCQAH
GLTPEQVVAIASNGGGKQALETVQRLLPVLCQAHGLTPEQVVAIASNGGGKQALETVQRLL
PVLCQAHGLTPEQVVAIASHDGGKQALETVQRLLPVLCQAHGLTPAQVVAIASNIGGKQAL
ETVQRLLPVLCQAHGLTPDQVVAIASNGGGKQALETVQRLLPVLCQAHGLTPEQVVAIASN
GGGRPALESIVAQLSRPDPALAALTNDHLVALACLGGRPALDAVKKGLGGSPTPYPNYANA
GHVEGQSALFMRDNGISEGLVFHNNPEGTCGFCVNMTETLLPENAKMTVVPPEGAIPVKR
GATGETKVFTGNSNSPKSPTKGGCSGGSTNLSDIIEKETGKQLVIQESILMLPEEVEEVIGNK
PESDILVHTAYDESTDENVMLLTSDAPEYKPWALVIQDSNGENKIKML*

- **DdCBE (L) – CoII-L1 - G1333-C:**

MASVLTPLLLRGLTGSARRLPVPRAKIHSLDYKDHDGDYKDHDIDYKDDDDKMDIADLRTLG
YSQQQQEKIKPKVRSTVAQHHEALVGHGFTHAHIVALSQHPAALGTVAVKYQDMIAALPEA
THEAIVGVGKQWSGARALEALLTVAGELRGPPLQLDTGQLLKIAKRGGVTAVEAVHAWRN
ALTGAPLNLTPQQVVAIASNIGGKQALETVQRLLPVLCQAHGLTPDQVVAIASNIGGKQALE
TVQRLLPVLCQAHGLTPDQVVAIASHDGGKQALETVQRLLPVLCQAHGLTPAQVVAIASHD
GGKQALETVQRLLPVLCQAHGLTPAQVVAIASNNGGKQALETVQRLLPVLCQAHGLTPEQV
VAIASNGGGKQALETVQRLLPVLCQAHGLTPEQVVAIASNGGGKQALETVQRLLPVLCQAH
GLTPEQVVAIASNIGGKQALETVQRLLPVLCQAHGLTPDQVVAIASNIGGKQALETVQRLLP
VLCQAHGLTPDQVVAIASNIGGKQALETVQRLLPVLCQAHGLTPDQVVAIASNIGGKQALET
VQRLLPVLCQAHGLTPDQVVAIASHDGGKQALETVQRLLPVLCQAHGLTPAQVVAIASHDG
GKQALETVQRLLPVLCQAHGLTPAQVVAIASNIGGKQALETVQRLLPVLCQAHGLTPDQVV
AIASNGGGKQALETVQRLLPVLCQAHGLTPEQVVAIASNIGGRPALESIVAQLSRPDPALAA
LTNDHLVALACLGGRPALDAVKKGLGGSPTPYPNYANAGHVEGQSALFMRDNGISEGLVF
HNNPEGTCGFCVNMTETLLPENAKMTVVPPEGAIPVKRGATGETKVFTGNSNSPKSPTKG
GCSGGSTNLSDIIEKETGKQLVIQESILMLPEEVEEVIGNKPESDILVHTAYDESTDENVMLLT
SDAPEYKPWALVIQDSNGENKIKML*

- **DdCBE (L) – CoII-L2 - G1333-C:**

MASVLTPLLLRGLTGSARRLPVPRAKIHSLDYKDHDGDYKDHDIDYKDDDDKMDIADLRTLG
YSQQQQEKIKPKVRSTVAQHHEALVGHGFTHAHIVALSQHPAALGTVAVKYQDMIAALPEA
THEAIVGVGKQWSGARALEALLTVAGELRGPPLQLDTGQLLKIAKRGGVTAVEAVHAWRN
ALTGAPLNLTPQQVVAIASNGGGKQALETVQRLLPVLCQAHGLTPEQVVAIASNIGGKQALE
TVQRLLPVLCQAHGLTPDQVVAIASNIGGKQALETVQRLLPVLCQAHGLTPDQVVAIASNIG
GKQALETVQRLLPVLCQAHGLTPDQVVAIASNIGGKQALETVQRLLPVLCQAHGLTPDQVV
AIASHDGGKQALETVQRLLPVLCQAHGLTPAQVVAIASHDGGKQALETVQRLLPVLCQAHG
LTPAQVVAIASNIGGKQALETVQRLLPVLCQAHGLTPDQVVAIASNGGGKQALETVQRLLPV
LCQAHGLTPEQVVAIASNIGGKQALETVQRLLPVLCQAHGLTPDQVVAIASNNGGKQALET
VQRLLPVLCQAHGLTPEQVVAIASNNGGKQALETVQRLLPVLCQAHGLTPEQVVAIASNNG

GRPALESIVAQLSRPDPALAALTNDHLVALACLGGRPALDAVKKGLGGSPTPYPNYANAGH
VEGQSALFMRDNGISEGLVFHNNPEGTCGFCVNMTETLLPENAKMTVVPPEGAIPVKRGA
TGETKVFTGNSNSPKSPTKGGCSGGSTNLSDIIEKETGKQLVIQESILMLPEEVEEVIGNKPE
SDILVHTAYDESTDENVMLLTSDAPEYKPWALVIQDSNGENKIKML*

- **DdCBE (L) – CoIII-L1 - G1333-C:**

MASVLTPLLLRGLTGSARRLPVPRAKIHSLDYKDHDGDYKDHDIDYKDDDDKMDIADLRTLG
YSQQQQEKIKPKVRSTVAQHHEALVGHGFTHAHIVALSQHPAALGTVAVKYQDMIAALPEA
THEAIVGVGKQWSGARALEALLTVAGELRGPPLQLDTGQLLKIAKRGGVTAVEAVHAWRN
ALTGAPLNLTPQQVVAIASHDGGKQALETVQRLLPVLCQAHGLTPAQVVAIASNGGGKQAL
ETVQRLLPVLCQAHGLTPEQVVAIASNIGGKQALETVQRLLPVLCQAHGLTPDQVVAIASNN
GGKQALETVQRLLPVLCQAHGLTPEQVVAIASHDGGKQALETVQRLLPVLCQAHGLTPAQV
VAIASNIGGKQALETVQRLLPVLCQAHGLTPDQVVAIASNGGGKQALETVQRLLPVLCQAH
GLTPEQVVAIASHDGGKQALETVQRLLPVLCQAHGLTPAQVVAIASNIGGKQALETVQRLLP
VLCQAHGLTPDQVVAIASNNGGKQALETVQRLLPVLCQAHGLTPEQVVAIASNNGGKQALE
TVQRLLPVLCQAHGLTPEQVVAIASNGGGKQALETVQRLLPVLCQAHGLTPEQVVAIASNN
GGKQALETVQRLLPVLCQAHGLTPEQVVAIASNGGGKQALETVQRLLPVLCQAHGLTPEQ
VVAIASNGGGKQALETVQRLLPVLCQAHGLTPEQVVAIASNGGGRPALESIVAQLSRPDPAL
AALTNDHLVALACLGGRPALDAVKKGLGGSPTPYPNYANAGHVEGQSALFMRDNGISEGL
VFHNNPEGTCGFCVNMTETLLPENAKMTVVPPEGAIPVKRGATGETKVFTGNSNSPKSPT
KGGCSGGSTNLSDIIEKETGKQLVIQESILMLPEEVEEVIGNKPESDILVHTAYDESTDENVM
LLTSDAPEYKPWALVIQDSNGENKIKML*

- **DdCBE (L) – CoIII-L2 - G1333-C:**

MASVLTPLLLRGLTGSARRLPVPRAKIHSLDYKDHDGDYKDHDIDYKDDDDKMDIADLRTLG
YSQQQQEKIKPKVRSTVAQHHEALVGHGFTHAHIVALSQHPAALGTVAVKYQDMIAALPEA
THEAIVGVGKQWSGARALEALLTVAGELRGPPLQLDTGQLLKIAKRGGVTAVEAVHAWRN
ALTGAPLNLTPQQVVAIASNIGGKQALETVQRLLPVLCQAHGLTPDQVVAIASNNGGKQALE
TVQRLLPVLCQAHGLTPEQVVAIASHDGGKQALETVQRLLPVLCQAHGLTPAQVVAIASNIG
GKQALETVQRLLPVLCQAHGLTPDQVVAIASNGGGKQALETVQRLLPVLCQAHGLTPEQVV
AIASHDGGKQALETVQRLLPVLCQAHGLTPAQVVAIASNIGGKQALETVQRLLPVLCQAHGL
TPDQVVAIASNNGGKQALETVQRLLPVLCQAHGLTPEQVVAIASNNGGKQALETVQRLLPV
LCQAHGLTPEQVVAIASNGGGKQALETVQRLLPVLCQAHGLTPEQVVAIASNNGGKQALET
VQRLLPVLCQAHGLTPEQVVAIASNGGGKQALETVQRLLPVLCQAHGLTPEQVVAIASNGG
GRPALESIVAQLSRPDPALAALTNDHLVALACLGGRPALDAVKKGLGGSPTPYPNYANAGH
VEGQSALFMRDNGISEGLVFHNNPEGTCGFCVNMTETLLPENAKMTVVPPEGAIPVKRGA
TGETKVFTGNSNSPKSPTKGGCSGGSTNLSDIIEKETGKQLVIQESILMLPEEVEEVIGNKPE
SDILVHTAYDESTDENVMLLTSDAPEYKPWALVIQDSNGENKIKML*

- **DdCBE (L) – Atp6-L1 - G1333-C:**

MASVLTPLLLRGLTGSARRLPVPRAKIHSLDYKDHDGDYKDHDIDYKDDDDKMDIADLRTLG
YSQQQQEKIKPKVRSTVAQHHEALVGHGFTHAHIVALSQHPAALGTVAVKYQDMIAALPEA
THEAIVGVGKRGAGARALEALLTVAGELRGPPLQLDTGQLLKIAKRGGVTAVEAVHAWRNA
LTGAPLNLTPQQVVAIASNIGGKQALETVQRLLPVLCQAHGLTPDQVVAIASHDGGKQALET
VQRLLPVLCQAHGLTPAQVVAIASHDGGKQALETVQRLLPVLCQAHGLTPAQVVAIASNNG
GKQALETVQRLLPVLCQAHGLTPEQVVAIASNGGGKQALETVQRLLPVLCQAHGLTPEQVV
AIASHDGGKQALETVQRLLPVLCQAHGLTPAQVVAIASNGGGKQALETVQRLLPVLCQAHG
LTPEQVVAIASHDGGKQALETVQRLLPVLCQAHGLTPAQVVAIASHDGGKQALETVQRLLP
VLCQAHGLTPAQVVAIASNIGGKQALETVQRLLPVLCQAHGLTPDQVVAIASNGGGKQALE
TVQRLLPVLCQAHGLTPEQVVAIASNGGGKQALETVQRLLPVLCQAHGLTPEQVVAIASHD
GGKQALETVQRLLPVLCQAHGLTPAQVVAIASNGGGKQALETVQRLLPVLCQAHGLTPEQ

VVAIASNGGGKQALETVQRLLPVLCQAHGLTPEQVVAIASNGGGRPALESIVAQLSRPDPAL
AALTNDHLVALACLGGRPALDAVKKGLGGSPTPYPNYANAGHVEGQSALFMRDNGISEGL
VFHNNPEGTCGFCVNMTETLLPENAKMTVVPPEGAIPVKRGATGETKVFTGNSNSPKSPT
KGGCSGGSTNLSDIIEKETGKQLVIQESILMLPEEVEEVIGNKPESDILVHTAYDESTDENVM
LLTSDAPEYKPWALVIQDSNGENKIKML*

- **DdCBE (L) – Atp6-L2 - G1333-C:**

MASVLTPLLLRGLTGSARRLPVPRAKIHSLDYKDHDGDYKDHDIDYKDDDDKMDIADLRTLG
YSQQQQEKIKPKVRSTVAQHHEALVGHGFTHAHIVALSQHPAALGTVAVKYQDMIAALPEA
THEAIVGVGKQWSGARALEALLTVAGELRGPPLQLDTGQLLKIAKRGGVTAVEAVHAWRN
ALTGAPLNLTPQQVVAIASHDGGKQALETVQRLLPVLCQAHGLTPAQVVAIASNGGGKQAL
ETVQRLLPVLCQAHGLTPEQVVAIASHDGGKQALETVQRLLPVLCQAHGLTPAQVVAIASH
DGGKQALETVQRLLPVLCQAHGLTPAQVVAIASNIGGKQALETVQRLLPVLCQAHGLTPDQ
VVAIASNGGGKQALETVQRLLPVLCQAHGLTPEQVVAIASNGGGKQALETVQRLLPVLCQA
HGLTPEQVVAIASHDGGKQALETVQRLLPVLCQAHGLTPAQVVAIASNGGGKQALETVQRL
LPVLCQAHGLTPEQVVAIASNGGGKQALETVQRLLPVLCQAHGLTPEQVVAIASNGGGKQA
LETVQRLLPVLCQAHGLTPEQVVAIASHDGGKQALETVQRLLPVLCQAHGLTPAQVVAIASH
DGGRPALESIVAQLSRPDPALAALTNDHLVALACLGGRPALDAVKKGLGGSPTPYPNYANA
GHVEGQSALFMRDNGISEGLVFHNNPEGTCGFCVNMTETLLPENAKMTVVPPEGAIPVKR
GATGETKVFTGNSNSPKSPTKGGCSGGSTNLSDIIEKETGKQLVIQESILMLPEEVEEVIGNK
PESDILVHTAYDESTDENVMLLTSDAPEYKPWALVIQDSNGENKIKML*

- **DdCBE (L) – Atp8-L1 - G1333-C:**

MASVLTPLLLRGLTGSARRLPVPRAKIHSLDYKDHDGDYKDHDIDYKDDDDKMDIADLRTLG
YSQQQQEKIKPKVRSTVAQHHEALVGHGFTHAHIVALSQHPAALGTVAVKYQDMIAALPEA
THEAIVGVGKQWSGARALEALLTVAGELRGPPLQLDTGQLLKIAKRGGVTAVEAVHAWRN
ALTGAPLNLTPQQVVAIASNNGGKQALETVQRLLPVLCQAHGLTPEQVVAIASHDGGKQAL
ETVQRLLPVLCQAHGLTPAQVVAIASHDGGKQALETVQRLLPVLCQAHGLTPAQVVAIASNI
GGKQALETVQRLLPVLCQAHGLTPDQVVAIASHDGGKQALETVQRLLPVLCQAHGLTPAQ
VVAIASNIGGKQALETVQRLLPVLCQAHGLTPDQVVAIASNIGGKQALETVQRLLPVLCQAH
GLTPDQVVAIASHDGGKQALETVQRLLPVLCQAHGLTPAQVVAIASNGGGKQALETVQRLL
PVLCQAHGLTPEQVVAIASNIGGKQALETVQRLLPVLCQAHGLTPDQVVAIASNNGGKQAL
ETVQRLLPVLCQAHGLTPEQVVAIASNIGGKQALETVQRLLPVLCQAHGLTPDQVVAIASNG
GGKQALETVQRLLPVLCQAHGLTPEQVVAIASNIGGKQALETVQRLLPVLCQAHGLTPDQV
VAIASHDGGRPALESIVAQLSRPDPALAALTNDHLVALACLGGRPALDAVKKGLGGSPTPYP
NYANAGHVEGQSALFMRDNGISEGLVFHNNPEGTCGFCVNMTETLLPENAKMTVVPPEGA
IPVKRGATGETKVFTGNSNSPKSPTKGGCSGGSTNLSDIIEKETGKQLVIQESILMLPEEVEE
VIGNKPESDILVHTAYDESTDENVMLLTSDAPEYKPWALVIQDSNGENKIKML*

- **DdCBE (L) – Atp8-L2 - G1333-C:**

MASVLTPLLLRGLTGSARRLPVPRAKIHSLDYKDHDGDYKDHDIDYKDDDDKMDIADLRTLG
YSQQQQEKIKPKVRSTVAQHHEALVGHGFTHAHIVALSQHPAALGTVAVKYQDMIAALPEA
THEAIVGVGKRGAGARALEALLTVAGELRGPPLQLDTGQLLKIAKRGGVTAVEAVHAWRNA
LTGAPLNLTPQQVVAIASHDGGKQALETVQRLLPVLCQAHGLTPAQVVAIASHDGGKQALE
TVQRLLPVLCQAHGLTPAQVVAIASNIGGKQALETVQRLLPVLCQAHGLTPDQVVAIASHDG
GKQALETVQRLLPVLCQAHGLTPAQVVAIASNIGGKQALETVQRLLPVLCQAHGLTPDQVV
AIASNIGGKQALETVQRLLPVLCQAHGLTPDQVVAIASHDGGKQALETVQRLLPVLCQAHGL
TPAQVVAIASNGGGKQALETVQRLLPVLCQAHGLTPEQVVAIASNIGGKQALETVQRLLPVL
CQAHGLTPDQVVAIASNNGGKQALETVQRLLPVLCQAHGLTPEQVVAIASNIGGKQALETV
QRLLPVLCQAHGLTPDQVVAIASNGGGKQALETVQRLLPVLCQAHGLTPEQVVAIASNIGG

KQALETVQRLLPVLCQAHGLTPDQVVAIASHDGGKQALETVQRLLPVLCQAHGLTPAQVVA
IASNIGGRPALESIVAQLSRPDPALAALTNDHLVALACLGGRPALDAVKKGLGGSPTPYPNY
ANAGHVEGQSALFMRDNGISEGLVFHNNPEGTCGFCVNMTETLLPENAKMTVVPPEGAIP
VKRGATGETKVFTGNSNSPKSPTKGGCSGGSTNLSDIIEKETGKQLVIQESILMLPEEVEEVI
GNKPESDILVHTAYDESTDENVMLLTSDAPEYKPWALVIQDSNGENKIKML*

The halves used to target the H-strand - DdCBE (H) – have the general architecture of (from N- to C-terminus):

SOD2 MTS – 3xHA – TALE – 2aa linker – DddAtox split – 4aa linker – UGI*

- **DdCBE (H) – Nd1-H1 - G1333-N:**

MALSRAVCGTSRQLAPVLGYLGSRQKHSLPDYPYDVPDYAGYPYDVPDYAGYPYDVPDY
AMDIADLRTLGYSQQQQEKIKPKVRSTVAQHHEALVGHGFTHAHIVALSQHPAALGTVAVK
YQDMIAALPEATHEAIVGVGKQWSGARALEALLTVAGELRGPPLQLDTGQLLKIAKRGGVT
AVEAVHAWRNALTGAPLNLTPQQVVAIASNNGGKQALETVQRLLPVLCQAHGLTPEQVVAI
ASNGGGKQALETVQRLLPVLCQAHGLTPEQVVAIASNNGGKQALETVQRLLPVLCQAHGL
TPEQVVAIASNNGGKQALETVQRLLPVLCQAHGLTPEQVVAIASNGGGKQALETVQRLLPV
LCQAHGLTPEQVVAIASNIGGKQALETVQRLLPVLCQAHGLTPDQVVAIASNGGGKQALET
VQRLLPVLCQAHGLTPEQVVAIASNGGGKQALETVQRLLPVLCQAHGLTPEQVVAIASNNG
GKQALETVQRLLPVLCQAHGLTPEQVVAIASNNGGKQALETVQRLLPVLCQAHGLTPEQVV
AIASNGGGKQALETVQRLLPVLCQAHGLTPEQVVAIASNIGGKQALETVQRLLPVLCQAHGL
TPDQVVAIASNNGGRPALESIVAQLSRPDPALAALTNDHLVALACLGGRPALDAVKKGLGG
SGSYALGPYQISAPQLPAYNGQTVGTFYYVNDAGGLESKVFSSGGSGGSTNLSDIIEKETG
KQLVIQESILMLPEEVEEVIGNKPESDILVHTAYDESTDENVMLLTSDAPEYKPWALVIQDSN
GENKIKML*

- **DdCBE (H) – Nd1-H2 - G1333-N:**

MALSRAVCGTSRQLAPVLGYLGSRQKHSLPDYPYDVPDYAGYPYDVPDYAGYPYDVPDY
AMDIADLRTLGYSQQQQEKIKPKVRSTVAQHHEALVGHGFTHAHIVALSQHPAALGTVAVK
YQDMIAALPEATHEAIVGVGKQWSGARALEALLTVAGELRGPPLQLDTGQLLKIAKRGGVT
AVEAVHAWRNALTGAPLNLTPQQVVAIASNNGGKQALETVQRLLPVLCQAHGLTPEQVVAI
ASNNGGKQALETVQRLLPVLCQAHGLTPEQVVAIASNNGGKQALETVQRLLPVLCQAHGLT
PEQVVAIASNGGGKQALETVQRLLPVLCQAHGLTPEQVVAIASNNGGKQALETVQRLLPVL
CQAHGLTPEQVVAIASNGGGKQALETVQRLLPVLCQAHGLTPEQVVAIASNNGGKQALETV
QRLLPVLCQAHGLTPEQVVAIASNNGGKQALETVQRLLPVLCQAHGLTPEQVVAIASNGGG
KQALETVQRLLPVLCQAHGLTPEQVVAIASNIGGKQALETVQRLLPVLCQAHGLTPDQVVAI
ASNGGGKQALETVQRLLPVLCQAHGLTPEQVVAIASNGGGKQALETVQRLLPVLCQAHGL
TPEQVVAIASNNGGKQALETVQRLLPVLCQAHGLTPEQVVAIASNNGGKQALETVQRLLPV
LCQAHGLTPEQVVAIASNGGGRPALESIVAQLSRPDPALAALTNDHLVALACLGGRPALDA
VKKGLGGSGSYALGPYQISAPQLPAYNGQTVGTFYYVNDAGGLESKVFSSGGSGGSTNLS
DIIEKETGKQLVIQESILMLPEEVEEVIGNKPESDILVHTAYDESTDENVMLLTSDAPEYKPWA
LVIQDSNGENKIKML*

- **DdCBE (H) – Nd2-H1 - G1333-N:**

MALSRAVCGTSRQLAPVLGYLGSRQKHSLPDYPYDVPDYAGYPYDVPDYAGYPYDVPDY
AMDIADLRTLGYSQQQQEKIKPKVRSTVAQHHEALVGHGFTHAHIVALSQHPAALGTVAVK

YQDMIAALPEATHEAIVGVGKQWSGARALEALLTVAGELRGPPLQLDTGQLLKIAKRGGVT
AVEAVHAWRNALTGAPLNLTPQQVVAIASNIGGKQALETVQRLLPVLCQAHGLTPDQVVAIA
SNNGGKQALETVQRLLPVLCQAHGLTPEQVVAIASNNGGKQALETVQRLLPVLCQAHGLTP
EQVVAIASHDGGKQALETVQRLLPVLCQAHGLTPAQVVAIASNGGGKQALETVQRLLPVLC
QAHGLTPEQVVAIASNNGGKQALETVQRLLPVLCQAHGLTPEQVVAIASNIGGKQALETVQ
RLLPVLCQAHGLTPDQVVAIASNIGGKQALETVQRLLPVLCQAHGLTPDQVVAIASNGGGK
QALETVQRLLPVLCQAHGLTPEQVVAIASNGGGKQALETVQRLLPVLCQAHGLTPEQVVAI
ASHDGGKQALETVQRLLPVLCQAHGLTPAQVVAIASHDGGKQALETVQRLLPVLCQAHGLT
PAQVVAIASNIGGKQALETVQRLLPVLCQAHGLTPDQVVAIASNNGGRPALESIVAQLSRPD
PALAALTNDHLVALACLGGRPALDAVKKGLGGSGSYALGPYQISAPQLPAYNGQTVGTFYY
VNDAGGLESKVFSSGGSGGSTNLSDIIEKETGKQLVIQESILMLPEEVEEVIGNKPESDILVH
TAYDESTDENVMLLTSDAPEYKPWALVIQDSNGENKIKML*

- **DdCBE (H) – Nd2-H2 - G1333-N:**

MALSRAVCGTSRQLAPVLGYLGSRQKHSLPDYPYDVPDYAGYPYDVPDYAGYPYDVPDY
AMDIADLRTLGYSQQQQEKIKPKVRSTVAQHHEALVGHGFTHAHIVALSQHPAALGTVAVK
YQDMIAALPEATHEAIVGVGKQWSGARALEALLTVAGELRGPPLQLDTGQLLKIAKRGGVT
AVEAVHAWRNALTGAPLNLTPQQVVAIASNIGGKQALETVQRLLPVLCQAHGLTPDQVVAIA
SNNGGKQALETVQRLLPVLCQAHGLTPEQVVAIASNGGGKQALETVQRLLPVLCQAHGLT
PEQVVAIASNIGGKQALETVQRLLPVLCQAHGLTPDQVVAIASNNGGKQALETVQRLLPVLC
QAHGLTPEQVVAIASNNGGKQALETVQRLLPVLCQAHGLTPEQVVAIASHDGGKQALETVQ
RLLPVLCQAHGLTPAQVVAIASNGGGKQALETVQRLLPVLCQAHGLTPEQVVAIASNNGGK
QALETVQRLLPVLCQAHGLTPEQVVAIASNIGGKQALETVQRLLPVLCQAHGLTPDQVVAIA
SNIGGKQALETVQRLLPVLCQAHGLTPDQVVAIASNGGGKQALETVQRLLPVLCQAHGLTP
EQVVAIASNGGGKQALETVQRLLPVLCQAHGLTPEQVVAIASHDGGKQALETVQRLLPVLC
QAHGLTPAQVVAIASHDGGRPALESIVAQLSRPDPALAALTNDHLVALACLGGRPALDAVK
KGLGGSGSYALGPYQISAPQLPAYNGQTVGTFYYVNDAGGLESKVFSSGGSGGSTNLSDII
EKETGKQLVIQESILMLPEEVEEVIGNKPESDILVHTAYDESTDENVMLLTSDAPEYKPWALV
IQDSNGENKIKML*

- **DdCBE (H) – Nd3-H1 - G1333-N:**

MALSRAVCGTSRQLAPVLGYLGSRQKHSLPDYPYDVPDYAGYPYDVPDYAGYPYDVPDY
AMDIADLRTLGYSQQQQEKIKPKVRSTVAQHHEALVGHGFTHAHIVALSQHPAALGTVAVK
YQDMIAALPEATHEAIVGVGKQWSGARALEALLTVAGELRGPPLQLDTGQLLKIAKRGGVT
AVEAVHAWRNALTGAPLNLTPQQVVAIASNIGGKQALETVQRLLPVLCQAHGLTPDQVVAIA
SHDGGKQALETVQRLLPVLCQAHGLTPAQVVAIASNIGGKQALETVQRLLPVLCQAHGLTP
DQVVAIASNNGGKQALETVQRLLPVLCQAHGLTPEQVVAIASNIGGKQALETVQRLLPVLCQ
AHGLTPDQVVAIASNGGGKQALETVQRLLPVLCQAHGLTPEQVVAIASNGGGKQALETVQ
RLLPVLCQAHGLTPEQVVAIASNGGGKQALETVQRLLPVLCQAHGLTPEQVVAIASNIGGK
QALETVQRLLPVLCQAHGLTPDQVVAIASNGGGKQALETVQRLLPVLCQAHGLTPEQVVAI
ASNGGGKQALETVQRLLPVLCQAHGLTPEQVVAIASNGGGKQALETVQRLLPVLCQAHGL
TPEQVVAIASNNGGRPALESIVAQLSRPDPALAALTNDHLVALACLGGRPALDAVKKGLGG
SGSYALGPYQISAPQLPAYNGQTVGTFYYVNDAGGLESKVFSSGGSGGSTNLSDIIEKETG
KQLVIQESILMLPEEVEEVIGNKPESDILVHTAYDESTDENVMLLTSDAPEYKPWALVIQDSN
GENKIKML*

- **DdCBE (H) – Nd3-H2 - G1333-N:**

MALSRAVCGTSRQLAPVLGYLGSRQKHSLPDYPYDVPDYAGYPYDVPDYAGYPYDVPDY
AMDIADLRTLGYSQQQQEKIKPKVRSTVAQHHEALVGHGFTHAHIVALSQHPAALGTVAVK
YQDMIAALPEATHEAIVGVGKQWSGARALEALLTVAGELRGPPLQLDTGQLLKIAKRGGVT
AVEAVHAWRNALTGAPLNLTPQQVVAIASNNGGKQALETVQRLLPVLCQAHGLTPEQVVAI

ASNIGGKQALETVQRLLPVLCQAHGLTPDQVVAIASNNGGKQALETVQRLLPVLCQAHGLT
PEQVVAIASNGGGKQALETVQRLLPVLCQAHGLTPEQVVAIASNIGGKQALETVQRLLPVLC
QAHGLTPDQVVAIASHDGGKQALETVQRLLPVLCQAHGLTPAQVVAIASNIGGKQALETVQ
RLLPVLCQAHGLTPDQVVAIASNNGGKQALETVQRLLPVLCQAHGLTPEQVVAIASNIGGK
QALETVQRLLPVLCQAHGLTPDQVVAIASNGGGKQALETVQRLLPVLCQAHGLTPEQVVAI
ASNGGGKQALETVQRLLPVLCQAHGLTPEQVVAIASNGGGKQALETVQRLLPVLCQAHGL
TPEQVVAIASNIGGKQALETVQRLLPVLCQAHGLTPDQVVAIASNGGGKQALETVQRLLPVL
CQAHGLTPEQVVAIASNGGGKQALETVQRLLPVLCQAHGLTPEQVVAIASNGGGRPALESI
VAQLSRPDPALAALTNDHLVALACLGGRPALDAVKKGLGGSGSYALGPYQISAPQLPAYNG
QTVGTFYYVNDAGGLESKVFSSGGSGGSTNLSDIIEKETGKQLVIQESILMLPEEVEEVIGNK
PESDILVHTAYDESTDENVMLLTSDAPEYKPWALVIQDSNGENKIKML*

- **DdCBE (H) – Nd4-H1 - G1333-N:**

MALSRAVCGTSRQLAPVLGYLGSRQKHSLPDYPYDVPDYAGYPYDVPDYAGYPYDVPDY
AMDIADLRTLGYSQQQQEKIKPKVRSTVAQHHEALVGHGFTHAHIVALSQHPAALGTVAVK
YQDMIAALPEATHEAIVGVGKQWSGARALEALLTVAGELRGPPLQLDTGQLLKIAKRGGVT
AVEAVHAWRNALTGAPLNLTPQQVVAIASNGGGKQALETVQRLLPVLCQAHGLTPEQVVAI
ASNGGGKQALETVQRLLPVLCQAHGLTPEQVVAIASNGGGKQALETVQRLLPVLCQAHGL
TPEQVVAIASNGGGKQALETVQRLLPVLCQAHGLTPEQVVAIASNGGGKQALETVQRLLPV
LCQAHGLTPEQVVAIASNGGGKQALETVQRLLPVLCQAHGLTPEQVVAIASNIGGKQALET
VQRLLPVLCQAHGLTPDQVVAIASNNGGKQALETVQRLLPVLCQAHGLTPEQVVAIASNNG
GKQALETVQRLLPVLCQAHGLTPEQVVAIASNNGGKQALETVQRLLPVLCQAHGLTPEQVV
AIASHDGGKQALETVQRLLPVLCQAHGLTPAQVVAIASNGGGKQALETVQRLLPVLCQAHG
LTPEQVVAIASNGGGRPALESIVAQLSRPDPALAALTNDHLVALACLGGRPALDAVKKGLG
GSGSYALGPYQISAPQLPAYNGQTVGTFYYVNDAGGLESKVFSSGGSGGSTNLSDIIEKET
GKQLVIQESILMLPEEVEEVIGNKPESDILVHTAYDESTDENVMLLTSDAPEYKPWALVIQDS
NGENKIKML*

- **DdCBE (H) – Nd4-H2 - G1333-N:**

MALSRAVCGTSRQLAPVLGYLGSRQKHSLPDYPYDVPDYAGYPYDVPDYAGYPYDVPDY
AMDIADLRTLGYSQQQQEKIKPKVRSTVAQHHEALVGHGFTHAHIVALSQHPAALGTVAVK
YQDMIAALPEATHEAIVGVGKQWSGARALEALLTVAGELRGPPLQLDTGQLLKIAKRGGVT
AVEAVHAWRNALTGAPLNLTPQQVVAIASHDGGKQALETVQRLLPVLCQAHGLTPAQVVAI
ASNIGGKQALETVQRLLPVLCQAHGLTPDQVVAIASNNGGKQALETVQRLLPVLCQAHGLT
PEQVVAIASNNGGKQALETVQRLLPVLCQAHGLTPEQVVAIASNGGGKQALETVQRLLPVL
CQAHGLTPEQVVAIASNGGGKQALETVQRLLPVLCQAHGLTPEQVVAIASNGGGKQALETV
QRLLPVLCQAHGLTPEQVVAIASNGGGKQALETVQRLLPVLCQAHGLTPEQVVAIASNGGG
KQALETVQRLLPVLCQAHGLTPEQVVAIASNGGGKQALETVQRLLPVLCQAHGLTPEQVVA
IASNGGGKQALETVQRLLPVLCQAHGLTPEQVVAIASNIGGKQALETVQRLLPVLCQAHGLT
PDQVVAIASNNGGKQALETVQRLLPVLCQAHGLTPEQVVAIASNNGGKQALETVQRLLPVL
CQAHGLTPEQVVAIASNNGGKQALETVQRLLPVLCQAHGLTPEQVVAIASHDGGRPALESI
VAQLSRPDPALAALTNDHLVALACLGGRPALDAVKKGLGGSGSYALGPYQISAPQLPAYNG
QTVGTFYYVNDAGGLESKVFSSGGSGGSTNLSDIIEKETGKQLVIQESILMLPEEVEEVIGNK
PESDILVHTAYDESTDENVMLLTSDAPEYKPWALVIQDSNGENKIKML*

- **DdCBE (H) – Nd4l-H1 - G1333-N:**

MALSRAVCGTSRQLAPVLGYLGSRQKHSLPDYPYDVPDYAGYPYDVPDYAGYPYDVPDY
AMDIADLRTLGYSQQQQEKIKPKVRSTVAQHHEALVGHGFTHAHIVALSQHPAALGTVAVK
YQDMIAALPEATHEAIVGVGKQWSGARALEALLTVAGELRGPPLQLDTGQLLKIAKRGGVT
AVEAVHAWRNALTGAPLNLTPQQVVAIASNGGGKQALETVQRLLPVLCQAHGLTPEQVVAI
ASNNGGKQALETVQRLLPVLCQAHGLTPEQVVAIASNGGGKQALETVQRLLPVLCQAHGL

TPEQVVAIASNIGGKQALETVQRLLPVLCQAHGLTPDQVVAIASNNGGKQALETVQRLLPVL
CQAHGLTPEQVVAIASNGGGKQALETVQRLLPVLCQAHGLTPEQVVAIASNIGGKQALETV
QRLLPVLCQAHGLTPDQVVAIASNNGGKQALETVQRLLPVLCQAHGLTPEQVVAIASNNGG
KQALETVQRLLPVLCQAHGLTPEQVVAIASNGGGKQALETVQRLLPVLCQAHGLTPEQVVA
IASNGGGKQALETVQRLLPVLCQAHGLTPEQVVAIASNNGGKQALETVQRLLPVLCQAHGL
TPEQVVAIASNIGGRPALESIVAQLSRPDPALAALTNDHLVALACLGGRPALDAVKKGLGGS
GSYALGPYQISAPQLPAYNGQTVGTFYYVNDAGGLESKVFSSGGSGGSTNLSDIIEKETGK
QLVIQESILMLPEEVEEVIGNKPESDILVHTAYDESTDENVMLLTSDAPEYKPWALVIQDSNG
ENKIKML*

- **DdCBE (H) – Nd4I-H2 - G1333-N:**

MALSRAVCGTSRQLAPVLGYLGSRQKHSLPDYPYDVPDYAGYPYDVPDYAGYPYDVPDY
AMDIADLRTLGYSQQQQEKIKPKVRSTVAQHHEALVGHGFTHAHIVALSQHPAALGTVAVK
YQDMIAALPEATHEAIVGVGKQWSGARALEALLTVAGELRGPPLQLDTGQLLKIAKRGGVT
AVEAVHAWRNALTGAPLNLTPQQVVAIASNIGGKQALETVQRLLPVLCQAHGLTPDQVVAIA
SNNGGKQALETVQRLLPVLCQAHGLTPEQVVAIASHDGGKQALETVQRLLPVLCQAHGLTP
AQVVAIASNIGGKQALETVQRLLPVLCQAHGLTPDQVVAIASNGGGKQALETVQRLLPVLC
QAHGLTPEQVVAIASNGGGKQALETVQRLLPVLCQAHGLTPEQVVAIASNNGGKQALETVQ
RLLPVLCQAHGLTPEQVVAIASNGGGKQALETVQRLLPVLCQAHGLTPEQVVAIASNIGGK
QALETVQRLLPVLCQAHGLTPDQVVAIASNNGGKQALETVQRLLPVLCQAHGLTPEQVVAI
ASNGGGKQALETVQRLLPVLCQAHGLTPEQVVAIASNIGGKQALETVQRLLPVLCQAHGLT
PDQVVAIASNNGGKQALETVQRLLPVLCQAHGLTPEQVVAIASNNGGKQALETVQRLLPVL
CQAHGLTPEQVVAIASNGGGKQALETVQRLLPVLCQAHGLTPEQVVAIASNGGGRPALESI
VAQLSRPDPALAALTNDHLVALACLGGRPALDAVKKGLGGSGSYALGPYQISAPQLPAYNG
QTVGTFYYVNDAGGLESKVFSSGGSGGSTNLSDIIEKETGKQLVIQESILMLPEEVEEVIGNK
PESDILVHTAYDESTDENVMLLTSDAPEYKPWALVIQDSNGENKIKML*

- **DdCBE (H) – Nd5-H1 - G1333-N:**

MALSRAVCGTSRQLAPVLGYLGSRQKHSLPDYPYDVPDYAGYPYDVPDYAGYPYDVPDY
AMDIADLRTLGYSQQQQEKIKPKVRSTVAQHHEALVGHGFTHAHIVALSQHPAALGTVAVK
YQDMIAALPEATHEAIVGVGKQWSGARALEALLTVAGELRGPPLQLDTGQLLKIAKRGGVT
AVEAVHAWRNALTGAPLNLTPQQVVAIASHDGGKQALETVQRLLPVLCQAHGLTPAQVVAI
ASNGGGKQALETVQRLLPVLCQAHGLTPEQVVAIASNIGGKQALETVQRLLPVLCQAHGLT
PDQVVAIASNGGGKQALETVQRLLPVLCQAHGLTPEQVVAIASNGGGKQALETVQRLLPVL
CQAHGLTPEQVVAIASNNGGKQALETVQRLLPVLCQAHGLTPEQVVAIASNIGGKQALETV
QRLLPVLCQAHGLTPDQVVAIASNIGGKQALETVQRLLPVLCQAHGLTPDQVVAIASNGGG
KQALETVQRLLPVLCQAHGLTPEQVVAIASNGGGKQALETVQRLLPVLCQAHGLTPEQVVA
IASNGGGKQALETVQRLLPVLCQAHGLTPEQVVAIASNIGGKQALETVQRLLPVLCQAHGLT
PDQVVAIASNGGGRPALESIVAQLSRPDPALAALTNDHLVALACLGGRPALDAVKKGLGGS
GSYALGPYQISAPQLPAYNGQTVGTFYYVNDAGGLESKVFSSGGSGGSTNLSDIIEKETGK
QLVIQESILMLPEEVEEVIGNKPESDILVHTAYDESTDENVMLLTSDAPEYKPWALVIQDSNG
ENKIKML*

- **DdCBE (H) – Nd5-H2 - G1333-N:**

MALSRAVCGTSRQLAPVLGYLGSRQKHSLPDYPYDVPDYAGYPYDVPDYAGYPYDVPDY
AMDIADLRTLGYSQQQQEKIKPKVRSTVAQHHEALVGHGFTHAHIVALSQHPAALGTVAVK
YQDMIAALPEATHEAIVGVGKQWSGARALEALLTVAGELRGPPLQLDTGQLLKIAKRGGVT
AVEAVHAWRNALTGAPLNLTPQQVVAIASNIGGKQALETVQRLLPVLCQAHGLTPDQVVAIA
SNIGGKQALETVQRLLPVLCQAHGLTPDQVVAIASNNGGKQALETVQRLLPVLCQAHGLTP
EQVVAIASNGGGKQALETVQRLLPVLCQAHGLTPEQVVAIASNGGGKQALETVQRLLPVLC
QAHGLTPEQVVAIASHDGGKQALETVQRLLPVLCQAHGLTPAQVVAIASNGGGKQALETVQ

RLLPVLCQAHGLTPEQVVAIASNIGGKQALETVQRLLPVLCQAHGLTPDQVVAIASNGGGK
QALETVQRLLPVLCQAHGLTPEQVVAIASNGGGKQALETVQRLLPVLCQAHGLTPEQVVAI
ASNNGGKQALETVQRLLPVLCQAHGLTPEQVVAIASNIGGKQALETVQRLLPVLCQAHGLT
PDQVVAIASNIGGKQALETVQRLLPVLCQAHGLTPDQVVAIASNGGGKQALETVQRLLPVLC
QAHGLTPEQVVAIASNGGGKQALETVQRLLPVLCQAHGLTPEQVVAIASNGGGRPALESIV
AQLSRPDPALAALTNDHLVALACLGGRPALDAVKKGLGGSGSYALGPYQISAPQLPAYNGQ
TVGTFYYVNDAGGLESKVFSSGGSGGSTNLSDIIEKETGKQLVIQESILMLPEEVEEVIGNKP
ESDILVHTAYDESTDENVMLLTSDAPEYKPWALVIQDSNGENKIKML*

- **DdCBE (H) – Nd6-H1 - G1333-C:**

MALSRAVCGTSRQLAPVLGYLGSRQKHSLPDYPYDVPDYAGYPYDVPDYAGYPYDVPDY
AMDIADLRTLGYSQQQQEKIKPKVRSTVAQHHEALVGHGFTHAHIVALSQHPAALGTVAVK
YQDMIAALPEATHEAIVGVGKQWSGARALEALLTVAGELRGPPLQLDTGQLLKIAKRGGVT
AVEAVHAWRNALTGAPLNLTPQQVVAIASHDGGKQALETVQRLLPVLCQAHGLTPAQVVAI
ASHDGGKQALETVQRLLPVLCQAHGLTPAQVVAIASNIGGKQALETVQRLLPVLCQAHGLT
PDQVVAIASNNGGKQALETVQRLLPVLCQAHGLTPEQVVAIASNIGGKQALETVQRLLPVLC
QAHGLTPDQVVAIASNNGGKQALETVQRLLPVLCQAHGLTPEQVVAIASNIGGKQALETVQ
RLLPVLCQAHGLTPDQVVAIASHDGGKQALETVQRLLPVLCQAHGLTPAQVVAIASNGGGK
QALETVQRLLPVLCQAHGLTPEQVVAIASNGGGKQALETVQRLLPVLCQAHGLTPEQVVAI
ASNNGGKQALETVQRLLPVLCQAHGLTPEQVVAIASNNGGKQALETVQRLLPVLCQAHGLT
PEQVVAIASNNGGKQALETVQRLLPVLCQAHGLTPEQVVAIASNNGGRPALESIVAQLSRP
DPALAALTNDHLVALACLGGRPALDAVKKGLGGSPTPYPNYANAGHVEGQSALFMRDNGI
SEGLVFHNNPEGTCGFCVNMTETLLPENAKMTVVPPEGAIPVKRGATGETKVFTGNSNSP
KSPTKGGCSGGSTNLSDIIEKETGKQLVIQESILMLPEEVEEVIGNKPESDILVHTAYDESTD
ENVMLLTSDAPEYKPWALVIQDSNGENKIKML*

- **DdCBE (H) – Nd6-H2 - G1333-C:**

MALSRAVCGTSRQLAPVLGYLGSRQKHSLPDYPYDVPDYAGYPYDVPDYAGYPYDVPDY
AMDIADLRTLGYSQQQQEKIKPKVRSTVAQHHEALVGHGFTHAHIVALSQHPAALGTVAVK
YQDMIAALPEATHEAIVGVGKQWSGARALEALLTVAGELRGPPLQLDTGQLLKIAKRGGVT
AVEAVHAWRNALTGAPLNLTPQQVVAIASNIGGKQALETVQRLLPVLCQAHGLTPDQVVAIA
SNGGGKQALETVQRLLPVLCQAHGLTPEQVVAIASHDGGKQALETVQRLLPVLCQAHGLT
PAQVVAIASHDGGKQALETVQRLLPVLCQAHGLTPAQVVAIASNIGGKQALETVQRLLPVLC
QAHGLTPDQVVAIASNNGGKQALETVQRLLPVLCQAHGLTPEQVVAIASNIGGKQALETVQ
RLLPVLCQAHGLTPDQVVAIASNNGGKQALETVQRLLPVLCQAHGLTPEQVVAIASNIGGK
QALETVQRLLPVLCQAHGLTPDQVVAIASHDGGKQALETVQRLLPVLCQAHGLTPAQVVAI
ASNGGGKQALETVQRLLPVLCQAHGLTPEQVVAIASNGGGKQALETVQRLLPVLCQAHGL
TPEQVVAIASNNGGKQALETVQRLLPVLCQAHGLTPEQVVAIASNNGGKQALETVQRLLPV
LCQAHGLTPEQVVAIASNNGGKQALETVQRLLPVLCQAHGLTPEQVVAIASNNGGKQALET
VQRLLPVLCQAHGLTPEQVVAIASNIGGRPALESIVAQLSRPDPALAALTNDHLVALACLGG
RPALDAVKKGLGGSPTPYPNYANAGHVEGQSALFMRDNGISEGLVFHNNPEGTCGFCVN
MTETLLPENAKMTVVPPEGAIPVKRGATGETKVFTGNSNSPKSPTKGGCSGGSTNLSDIIEK
ETGKQLVIQESILMLPEEVEEVIGNKPESDILVHTAYDESTDENVMLLTSDAPEYKPWALVIQ
DSNGENKIKML*

- **DdCBE (H) – Cytb-H1 - G1333-N:**

MALSRAVCGTSRQLAPVLGYLGSRQKHSLPDYPYDVPDYAGYPYDVPDYAGYPYDVPDY
AMDIADLRTLGYSQQQQEKIKPKVRSTVAQHHEALVGHGFTHAHIVALSQHPAALGTVAVK
YQDMIAALPEATHEAIVGVGKQWSGARALEALLTVAGELRGPPLQLDTGQLLKIAKRGGVT
AVEAVHAWRNALTGAPLNLTPQQVVAIASNNGGKQALETVQRLLPVLCQAHGLTPEQVVAI
ASHDGGKQALETVQRLLPVLCQAHGLTPAQVVAIASNNGGKQALETVQRLLPVLCQAHGLT
PEQVVAIASNGGGKQALETVQRLLPVLCQAHGLTPEQVVAIASNNGGKQALETVQRLLPVL

CQAHGLTPEQVVAIASNGGGKQALETVQRLLPVLCQAHGLTPEQVVAIASNIGGKQALETV
QRLLPVLCQAHGLTPDQVVAIASNGGGKQALETVQRLLPVLCQAHGLTPEQVVAIASNIGG
KQALETVQRLLPVLCQAHGLTPDQVVAIASNGGGKQALETVQRLLPVLCQAHGLTPEQVVA
IASNIGGKQALETVQRLLPVLCQAHGLTPDQVVAIASNGGGKQALETVQRLLPVLCQAHGLT
PEQVVAIASHDGGKQALETVQRLLPVLCQAHGLTPAQVVAIASNNGGRPALESIVAQLSRP
DPALAALTNDHLVALACLGGRPALDAVKKGLGGSGSYALGPYQISAPQLPAYNGQTVGTFY
YVNDAGGLESKVFSSGGSGGSTNLSDIIEKETGKQLVIQESILMLPEEVEEVIGNKPESDILV
HTAYDESTDENVMLLTSDAPEYKPWALVIQDSNGENKIKML*

- **DdCBE (H) – Cytb-H2 - G1333-N:**

MALSRAVCGTSRQLAPVLGYLGSRQKHSLPDYPYDVPDYAGYPYDVPDYAGYPYDVPDY
AMDIADLRTLGYSQQQQEKIKPKVRSTVAQHHEALVGHGFTHAHIVALSQHPAALGTVAVK
YQDMIAALPEATHEAIVGVGKQWSGARALEALLTVAGELRGPPLQLDTGQLLKIAKRGGVT
AVEAVHAWRNALTGAPLNLTPQQVVAIASNGGGKQALETVQRLLPVLCQAHGLTPEQVVAI
ASNGGGKQALETVQRLLPVLCQAHGLTPEQVVAIASNNGGKQALETVQRLLPVLCQAHGL
TPEQVVAIASHDGGKQALETVQRLLPVLCQAHGLTPAQVVAIASNNGGKQALETVQRLLPV
LCQAHGLTPEQVVAIASNGGGKQALETVQRLLPVLCQAHGLTPEQVVAIASNNGGKQALET
VQRLLPVLCQAHGLTPEQVVAIASNGGGKQALETVQRLLPVLCQAHGLTPEQVVAIASNIG
GKQALETVQRLLPVLCQAHGLTPDQVVAIASNGGGKQALETVQRLLPVLCQAHGLTPEQVV
AIASNIGGKQALETVQRLLPVLCQAHGLTPDQVVAIASNGGGKQALETVQRLLPVLCQAHGL
TPEQVVAIASNIGGKQALETVQRLLPVLCQAHGLTPDQVVAIASNGGGRPALESIVAQLSRP
DPALAALTNDHLVALACLGGRPALDAVKKGLGGSGSYALGPYQISAPQLPAYNGQTVGTFY
YVNDAGGLESKVFSSGGSGGSTNLSDIIEKETGKQLVIQESILMLPEEVEEVIGNKPESDILV
HTAYDESTDENVMLLTSDAPEYKPWALVIQDSNGENKIKML*

- **DdCBE (H) – Col-H1 - G1333-N:**

MALSRAVCGTSRQLAPVLGYLGSRQKHSLPDYPYDVPDYAGYPYDVPDYAGYPYDVPDY
AMDIADLRTLGYSQQQQEKIKPKVRSTVAQHHEALVGHGFTHAHIVALSQHPAALGTVAVK
YQDMIAALPEATHEAIVGVGKQWSGARALEALLTVAGELRGPPLQLDTGQLLKIAKRGGVT
AVEAVHAWRNALTGAPLNLTPQQVVAIASNGGGKQALETVQRLLPVLCQAHGLTPEQVVAI
ASNNGGKQALETVQRLLPVLCQAHGLTPEQVVAIASNGGGKQALETVQRLLPVLCQAHGL
TPEQVVAIASNNGGKQALETVQRLLPVLCQAHGLTPEQVVAIASNIGGKQALETVQRLLPVL
CQAHGLTPDQVVAIASNGGGKQALETVQRLLPVLCQAHGLTPEQVVAIASNGGGKQALET
VQRLLPVLCQAHGLTPEQVVAIASNNGGKQALETVQRLLPVLCQAHGLTPEQVVAIASNNG
GKQALETVQRLLPVLCQAHGLTPEQVVAIASNGGGKQALETVQRLLPVLCQAHGLTPEQVV
AIASNGGGKQALETVQRLLPVLCQAHGLTPEQVVAIASNNGGKQALETVQRLLPVLCQAHG
LTPEQVVAIASNIGGRPALESIVAQLSRPDPALAALTNDHLVALACLGGRPALDAVKKGLGG
SGSYALGPYQISAPQLPAYNGQTVGTFYYVNDAGGLESKVFSSGGSGGSTNLSDIIEKETG
KQLVIQESILMLPEEVEEVIGNKPESDILVHTAYDESTDENVMLLTSDAPEYKPWALVIQDSN
GENKIKML*

- **DdCBE (H) – Col-H2 - G1333-N:**

MALSRAVCGTSRQLAPVLGYLGSRQKHSLPDYPYDVPDYAGYPYDVPDYAGYPYDVPDY
AMDIADLRTLGYSQQQQEKIKPKVRSTVAQHHEALVGHGFTHAHIVALSQHPAALGTVAVK
YQDMIAALPEATHEAIVGVGKQWSGARALEALLTVAGELRGPPLQLDTGQLLKIAKRGGVT
AVEAVHAWRNALTGAPLNLTPQQVVAIASNIGGKQALETVQRLLPVLCQAHGLTPDQVVAIA
SNGGGKQALETVQRLLPVLCQAHGLTPEQVVAIASHDGGKQALETVQRLLPVLCQAHGLT
PAQVVAIASNGGGKQALETVQRLLPVLCQAHGLTPEQVVAIASNGGGKQALETVQRLLPVL
CQAHGLTPEQVVAIASNGGGKQALETVQRLLPVLCQAHGLTPEQVVAIASNNGGKQALETV
QRLLPVLCQAHGLTPEQVVAIASNGGGKQALETVQRLLPVLCQAHGLTPEQVVAIASNNGG
KQALETVQRLLPVLCQAHGLTPEQVVAIASNIGGKQALETVQRLLPVLCQAHGLTPDQVVAI

ASNGGGKQALETVQRLLPVLCQAHGLTPEQVVAIASNGGGKQALETVQRLLPVLCQAHGL
TPEQVVAIASNNGGKQALETVQRLLPVLCQAHGLTPEQVVAIASNNGGKQALETVQRLLPV
LCQAHGLTPEQVVAIASNGGGKQALETVQRLLPVLCQAHGLTPEQVVAIASNGGGRPALES
IVAQLSRPDPALAALTNDHLVALACLGGRPALDAVKKGLGGSGSYALGPYQISAPQLPAYN
GQTVGTFYYVNDAGGLESKVFSSGGSGGSTNLSDIIEKETGKQLVIQESILMLPEEVEEVIG
NKPESDILVHTAYDESTDENVMLLTSDAPEYKPWALVIQDSNGENKIKML*

- **DdCBE (H) – CoII-H1 - G1333-N:**

MALSRAVCGTSRQLAPVLGYLGSRQKHSLPDYPYDVPDYAGYPYDVPDYAGYPYDVPDY
AMDIADLRTLGYSQQQQEKIKPKVRSTVAQHHEALVGHGFTHAHIVALSQHPAALGTVAVK
YQDMIAALPEATHEAIVGVGKQWSGARALEALLTVAGELRGPPLQLDTGQLLKIAKRGGVT
AVEAVHAWRNALTGAPLNLTPQQVVAIASNIGGKQALETVQRLLPVLCQAHGLTPDQVVAIA
SNGGGKQALETVQRLLPVLCQAHGLTPEQVVAIASNIGGKQALETVQRLLPVLCQAHGLTP
DQVVAIASNGGGKQALETVQRLLPVLCQAHGLTPEQVVAIASNGGGKQALETVQRLLPVLC
QAHGLTPEQVVAIASHDGGKQALETVQRLLPVLCQAHGLTPAQVVAIASNNGGKQALETVQ
RLLPVLCQAHGLTPEQVVAIASNGGGKQALETVQRLLPVLCQAHGLTPEQVVAIASNIGGK
QALETVQRLLPVLCQAHGLTPDQVVAIASNNGGKQALETVQRLLPVLCQAHGLTPEQVVAI
ASHDGGKQALETVQRLLPVLCQAHGLTPAQVVAIASNGGGKQALETVQRLLPVLCQAHGL
TPEQVVAIASNGGGRPALESIVAQLSRPDPALAALTNDHLVALACLGGRPALDAVKKGLGG
SGSYALGPYQISAPQLPAYNGQTVGTFYYVNDAGGLESKVFSSGGSGGSTNLSDIIEKETG
KQLVIQESILMLPEEVEEVIGNKPESDILVHTAYDESTDENVMLLTSDAPEYKPWALVIQDSN
GENKIKML*

- **DdCBE (H) – CoII-H2 - G1333-N:**

MALSRAVCGTSRQLAPVLGYLGSRQKHSLPDYPYDVPDYAGYPYDVPDYAGYPYDVPDY
AMDIADLRTLGYSQQQQEKIKPKVRSTVAQHHEALVGHGFTHAHIVALSQHPAALGTVAVK
YQDMIAALPEATHEAIVGVGKQWSGARALEALLTVAGELRGPPLQLDTGQLLKIAKRGGVT
AVEAVHAWRNALTGAPLNLTPQQVVAIASHDGGKQALETVQRLLPVLCQAHGLTPAQVVAI
ASNIGGKQALETVQRLLPVLCQAHGLTPDQVVAIASNNGGKQALETVQRLLPVLCQAHGLT
PEQVVAIASNGGGKQALETVQRLLPVLCQAHGLTPEQVVAIASNIGGKQALETVQRLLPVLC
QAHGLTPDQVVAIASNGGGKQALETVQRLLPVLCQAHGLTPEQVVAIASNIGGKQALETVQ
RLLPVLCQAHGLTPDQVVAIASNGGGKQALETVQRLLPVLCQAHGLTPEQVVAIASNGGGK
QALETVQRLLPVLCQAHGLTPEQVVAIASHDGGKQALETVQRLLPVLCQAHGLTPAQVVAI
ASNNGGKQALETVQRLLPVLCQAHGLTPEQVVAIASNGGGKQALETVQRLLPVLCQAHGL
TPEQVVAIASNIGGKQALETVQRLLPVLCQAHGLTPDQVVAIASNNGGKQALETVQRLLPVL
CQAHGLTPEQVVAIASHDGGRPALESIVAQLSRPDPALAALTNDHLVALACLGGRPALDAV
KKGLGGSGSYALGPYQISAPQLPAYNGQTVGTFYYVNDAGGLESKVFSSGGSGGSTNLSD
IIEKETGKQLVIQESILMLPEEVEEVIGNKPESDILVHTAYDESTDENVMLLTSDAPEYKPWAL
VIQDSNGENKIKML*

- **DdCBE (H) – CoIII-H1 - G1333-N:**

MALSRAVCGTSRQLAPVLGYLGSRQKHSLPDYPYDVPDYAGYPYDVPDYAGYPYDVPDY
AMDIADLRTLGYSQQQQEKIKPKVRSTVAQHHEALVGHGFTHAHIVALSQHPAALGTVAVK
YQDMIAALPEATHEAIVGVGKQWSGARALEALLTVAGELRGPPLQLDTGQLLKIAKRGGVT
AVEAVHAWRNALTGAPLNLTPQQVVAIASHDGGKQALETVQRLLPVLCQAHGLTPAQVVAI
ASNGGGKQALETVQRLLPVLCQAHGLTPEQVVAIASNIGGKQALETVQRLLPVLCQAHGLT
PDQVVAIASNGGGKQALETVQRLLPVLCQAHGLTPEQVVAIASNIGGKQALETVQRLLPVLC
QAHGLTPDQVVAIASNIGGKQALETVQRLLPVLCQAHGLTPDQVVAIASNNGGKQALETVQ
RLLPVLCQAHGLTPEQVVAIASNNGGKQALETVQRLLPVLCQAHGLTPEQVVAIASHDGGK

QALETVQRLLPVLCQAHGLTPAQVVAIASNGGGKQALETVQRLLPVLCQAHGLTPEQVVAI
ASNIGGKQALETVQRLLPVLCQAHGLTPDQVVAIASNGGGKQALETVQRLLPVLCQAHGLT
PEQVVAIASNNGGRPALESIVAQLSRPDPALAALTNDHLVALACLGGRPALDAVKKGLGGS
GSYALGPYQISAPQLPAYNGQTVGTFYYVNDAGGLESKVFSSGGSGGSTNLSDIIEKETGK
QLVIQESILMLPEEVEEVIGNKPESDILVHTAYDESTDENVMLLTSDAPEYKPWALVIQDSNG
ENKIKML*

- **DdCBE (H) – CoIII-H2 - G1333-N:**

MALSRAVCGTSRQLAPVLGYLGSRQKHSLPDYPYDVPDYAGYPYDVPDYAGYPYDVPDY
AMDIADLRTLGYSQQQQEKIKPKVRSTVAQHHEALVGHGFTHAHIVALSQHPAALGTVAVK
YQDMIAALPEATHEAIVGVGKQWSGARALEALLTVAGELRGPPLQLDTGQLLKIAKRGGVT
AVEAVHAWRNALTGAPLNLTPQQVVAIASNGGGKQALETVQRLLPVLCQAHGLTPEQVVAI
ASHDGGKQALETVQRLLPVLCQAHGLTPAQVVAIASNGGGKQALETVQRLLPVLCQAHGL
TPEQVVAIASNIGGKQALETVQRLLPVLCQAHGLTPDQVVAIASNGGGKQALETVQRLLPVL
CQAHGLTPEQVVAIASNIGGKQALETVQRLLPVLCQAHGLTPDQVVAIASNIGGKQALETVQ
RLLPVLCQAHGLTPDQVVAIASNNGGKQALETVQRLLPVLCQAHGLTPEQVVAIASNNGGK
QALETVQRLLPVLCQAHGLTPEQVVAIASHDGGKQALETVQRLLPVLCQAHGLTPAQVVAI
ASNGGGKQALETVQRLLPVLCQAHGLTPEQVVAIASNIGGKQALETVQRLLPVLCQAHGLT
PDQVVAIASNGGGKQALETVQRLLPVLCQAHGLTPEQVVAIASNNGGKQALETVQRLLPVL
CQAHGLTPEQVVAIASNIGGKQALETVQRLLPVLCQAHGLTPDQVVAIASNGGGRPALESIV
AQLSRPDPALAALTNDHLVALACLGGRPALDAVKKGLGGSGSYALGPYQISAPQLPAYNGQ
TVGTFYYVNDAGGLESKVFSSGGSGGSTNLSDIIEKETGKQLVIQESILMLPEEVEEVIGNKP
ESDILVHTAYDESTDENVMLLTSDAPEYKPWALVIQDSNGENKIKML*

- **DdCBE (H) – Atp6-H1 - G1333-N:**

MALSRAVCGTSRQLAPVLGYLGSRQKHSLPDYPYDVPDYAGYPYDVPDYAGYPYDVPDY
AMDIADLRTLGYSQQQQEKIKPKVRSTVAQHHEALVGHGFTHAHIVALSQHPAALGTVAVK
YQDMIAALPEATHEAIVGVGKQWSGARALEALLTVAGELRGPPLQLDTGQLLKIAKRGGVT
AVEAVHAWRNALTGAPLNLTPQQVVAIASNGGGKQALETVQRLLPVLCQAHGLTPEQVVAI
ASNNGGKQALETVQRLLPVLCQAHGLTPEQVVAIASNIGGKQALETVQRLLPVLCQAHGLT
PDQVVAIASNGGGKQALETVQRLLPVLCQAHGLTPEQVVAIASNIGGKQALETVQRLLPVLC
QAHGLTPDQVVAIASNIGGKQALETVQRLLPVLCQAHGLTPDQVVAIASNGGGKQALETVQ
RLLPVLCQAHGLTPEQVVAIASNIGGKQALETVQRLLPVLCQAHGLTPDQVVAIASNIGGKQ
ALETVQRLLPVLCQAHGLTPDQVVAIASNNGGKQALETVQRLLPVLCQAHGLTPEQVVAIAS
NGGGKQALETVQRLLPVLCQAHGLTPEQVVAIASNGGGKQALETVQRLLPVLCQAHGLTP
EQVVAIASNGGGRPALESIVAQLSRPDPALAALTNDHLVALACLGGRPALDAVKKGLGGSG
SYALGPYQISAPQLPAYNGQTVGTFYYVNDAGGLESKVFSSGGSGGSTNLSDIIEKETGKQ
LVIQESILMLPEEVEEVIGNKPESDILVHTAYDESTDENVMLLTSDAPEYKPWALVIQDSNGE
NKIKML*

- **DdCBE (H) – Atp6-H2 - G1333-N:**

MALSRAVCGTSRQLAPVLGYLGSRQKHSLPDYPYDVPDYAGYPYDVPDYAGYPYDVPDY
AMDIADLRTLGYSQQQQEKIKPKVRSTVAQHHEALVGHGFTHAHIVALSQHPAALGTVAVK
YQDMIAALPEATHEAIVGVGKQWSGARALEALLTVAGELRGPPLQLDTGQLLKIAKRGGVT
AVEAVHAWRNALTGAPLNLTPQQVVAIASNGGGKQALETVQRLLPVLCQAHGLTPEQVVAI
ASNGGGKQALETVQRLLPVLCQAHGLTPEQVVAIASNNGGKQALETVQRLLPVLCQAHGL
TPEQVVAIASNGGGKQALETVQRLLPVLCQAHGLTPEQVVAIASNGGGKQALETVQRLLPV
LCQAHGLTPEQVVAIASNGGGKQALETVQRLLPVLCQAHGLTPEQVVAIASNNGGKQALET
VQRLLPVLCQAHGLTPEQVVAIASNIGGKQALETVQRLLPVLCQAHGLTPDQVVAIASNGG
GKQALETVQRLLPVLCQAHGLTPEQVVAIASNIGGKQALETVQRLLPVLCQAHGLTPDQVV

AIASNIGGKQALETVQRLLPVLCQAHGLTPDQVVAIASNGGGKQALETVQRLLPVLCQAHGLTPEQVVAIASNIGGKQALETVQRLLPVLCQAHGLTPDQVVAIASNIGGKQALETVQRLLPVLCQAHGLTPDQVVAIASNNGGKQALETVQRLLPVLCQAHGLTPEQVVAIASNGGGRPALESIVAQLSRPDPALAALTNDHLVALACLGGRPALDAVKKGLGGSGSYALGPYQISAPQLPAYNGQTVGTFYYVNDAGGLESKVFSSGGSGGSTNLSDIIEKETGKQLVIQESILMLPEEVEEVIGNKPESDILVHTAYDESTDENVMLLTSDAPEYKPWALVIQDSNGENKIKML*

- **DdCBE (H) – Atp8-H1 - G1333-N:**

MALSRAVCGTSRQLAPVLGYLGSRQKHSLPDYPYDVPDYAGYPYDVPDYAGYPYDVPDYAMDIADLRTLGYSQQQQEKIKPKVRSTVAQHHEALVGHGFTHAHIVALSQHPAALGTVAVKYQDMIAALPEATHEAIVGVGKQWSGARALEALLTVAGELRGPPLQLDTGQLLKIAKRGGVTAVEAVHAWRNALTGAPLNLTPQQVVAIASNNGGKQALETVQRLLPVLCQAHGLTPEQVVAIASNIGGKQALETVQRLLPVLCQAHGLTPDQVVAIASNGGGKQALETVQRLLPVLCQAHGLTPEQVVAIASNNGGKQALETVQRLLPVLCQAHGLTPEQVVAIASNIGGKQALETVQRLLPVLCQAHGLTPDQVVAIASNNGGKQALETVQRLLPVLCQAHGLTPEQVVAIASNIGGKQALETVQRLLPVLCQAHGLTPDQVVAIASNGGGKQALETVQRLLPVLCQAHGLTPEQVVAIASNIGGKQALETVQRLLPVLCQAHGLTPDQVVAIASNIGGKQALETVQRLLPVLCQAHGLTPDQVVAIASNGGGKQALETVQRLLPVLCQAHGLTPEQVVAIASNGGGKQALETVQRLLPVLCQAHGLTPEQVVAIASNNGGKQALETVQRLLPVLCQAHGLTPEQVVAIASNGGGRPALESIVAQLSRPDPALAALTNDHLVALACLGGRPALDAVKKGLGGSGSYALGPYQISAPQLPAYNGQTVGTFYYVNDAGGLESKVFSSGGSGGSTNLSDIIEKETGKQLVIQESILMLPEEVEEVIGNKPESDILVHTAYDESTDENVMLLTSDAPEYKPWALVIQDSNGENKIKML*

- **DdCBE (H) – Atp8-H2 - G1333-N:**

MALSRAVCGTSRQLAPVLGYLGSRQKHSLPDYPYDVPDYAGYPYDVPDYAGYPYDVPDYAMDIADLRTLGYSQQQQEKIKPKVRSTVAQHHEALVGHGFTHAHIVALSQHPAALGTVAVKYQDMIAALPEATHEAIVGVGKQWSGARALEALLTVAGELRGPPLQLDTGQLLKIAKRGGVTAVEAVHAWRNALTGAPLNLTPQQVVAIASNGGGKQALETVQRLLPVLCQAHGLTPEQVVAIASNIGGKQALETVQRLLPVLCQAHGLTPDQVVAIASNGGGKQALETVQRLLPVLCQAHGLTPEQVVAIASNGGGKQALETVQRLLPVLCQAHGLTPEQVVAIASNNGGKQALETVQRLLPVLCQAHGLTPEQVVAIASNIGGKQALETVQRLLPVLCQAHGLTPDQVVAIASNGGGKQALETVQRLLPVLCQAHGLTPEQVVAIASNNGGKQALETVQRLLPVLCQAHGLTPEQVVAIASNIGGKQALETVQRLLPVLCQAHGLTPDQVVAIASNNGGKQALETVQRLLPVLCQAHGLTPEQVVAIASNIGGKQALETVQRLLPVLCQAHGLTPDQVVAIASNGGGKQALETVQRLLPVLCQAHGLTPEQVVAIASNIGGKQALETVQRLLPVLCQAHGLTPDQVVAIASNIGGKQALETVQRLLPVLCQAHGLTPDQVVAIASNGGGKQALETVQRLLPVLCQAHGLTPEQVVAIASNGGGRPALESIVAQLSRPDPALAALTNDHLVALACLGGRPALDAVKKGLGGSGSYALGPYQISAPQLPAYNGQTVGTFYYVNDAGGLESKVFSSGGSGGSTNLSDIIEKETGKQLVIQESILMLPEEVEEVIGNKPESDILVHTAYDESTDENVMLLTSDAPEYKPWALVIQDSNGENKIKML*

- **DdCBE (H) – Nd1-H1 - G1333-C:**

MALSRAVCGTSRQLAPVLGYLGSRQKHSLPDYPYDVPDYAGYPYDVPDYAGYPYDVPDYAMDIADLRTLGYSQQQQEKIKPKVRSTVAQHHEALVGHGFTHAHIVALSQHPAALGTVAVKYQDMIAALPEATHEAIVGVGKQWSGARALEALLTVAGELRGPPLQLDTGQLLKIAKRGGVTAVEAVHAWRNALTGAPLNLTPQQVVAIASNNGGKQALETVQRLLPVLCQAHGLTPEQVVAIASNGGGKQALETVQRLLPVLCQAHGLTPEQVVAIASNNGGKQALETVQRLLPVLCQAHGLTPEQVVAIASNNGGKQALETVQRLLPVLCQAHGLTPEQVVAIASNGGGKQALETVQRLLPVLCQAHGLTPEQVVAIASNIGGKQALETVQRLLPVLCQAHGLTPDQVVAIASNGGGKQALETVQRLLPVLCQAHGLTPEQVVAIASNGGGKQALETVQRLLPVLCQAHGLTPEQVVAIASNNGGKQALETVQRLLPVLCQAHGLTPEQVVAIASNNGGKQALETVQRLLPVLCQAHGLTPEQVVAIASNGGGKQALETVQRLLPVLCQAHGLTPEQVVAIASNIGGKQALETVQRLLPVLCQAHGL

TPDQVVAIASNNGGRPALESIVAQLSRPDPALAALTNDHLVALACLGGRPALDAVKKGLGG
SPTPYPNYANAGHVEGQSALFMRDNGISEGLVFHNNPEGTCGFCVNMTETLLPENAKMTV
VPPEGAIPVKRGATGETKVFTGNSNSPKSPTKGGCSGGSTNLSDIIEKETGKQLVIQESILML
PEEVEEVIGNKPESDILVHTAYDESTDENVMLLTSDAPEYKPWALVIQDSNGENKIKML*

- **DdCBE (H) – Nd1-H2 - G1333-C:**

MALSRAVCGTSRQLAPVLGYLGSRQKHSLPDYPYDVPDYAGYPYDVPDYAGYPYDVPDY
AMDIADLRTLGYSQQQQEKIKPKVRSTVAQHHEALVGHGFTHAHIVALSQHPAALGTVAVK
YQDMIAALPEATHEAIVGVGKQWSGARALEALLTVAGELRGPPLQLDTGQLLKIAKRGGVT
AVEAVHAWRNALTGAPLNLTPQQVVAIASNNGGKQALETVQRLLPVLCQAHGLTPEQVVAI
ASNNGGKQALETVQRLLPVLCQAHGLTPEQVVAIASNNGGKQALETVQRLLPVLCQAHGLT
PEQVVAIASNGGGKQALETVQRLLPVLCQAHGLTPEQVVAIASNNGGKQALETVQRLLPVL
CQAHGLTPEQVVAIASNGGGKQALETVQRLLPVLCQAHGLTPEQVVAIASNNGGKQALETV
QRLLPVLCQAHGLTPEQVVAIASNNGGKQALETVQRLLPVLCQAHGLTPEQVVAIASNGGG
KQALETVQRLLPVLCQAHGLTPEQVVAIASNIGGKQALETVQRLLPVLCQAHGLTPDQVVAI
ASNGGGKQALETVQRLLPVLCQAHGLTPEQVVAIASNGGGKQALETVQRLLPVLCQAHGL
TPEQVVAIASNNGGKQALETVQRLLPVLCQAHGLTPEQVVAIASNNGGKQALETVQRLLPV
LCQAHGLTPEQVVAIASNGGGRPALESIVAQLSRPDPALAALTNDHLVALACLGGRPALDA
VKKGLGGSPTPYPNYANAGHVEGQSALFMRDNGISEGLVFHNNPEGTCGFCVNMTETLLP
ENAKMTVVPPEGAIPVKRGATGETKVFTGNSNSPKSPTKGGCSGGSTNLSDIIEKETGKQL
VIQESILMLPEEVEEVIGNKPESDILVHTAYDESTDENVMLLTSDAPEYKPWALVIQDSNGEN
KIKML*

- **DdCBE (H) – Nd2-H1 - G1333-C:**

MALSRAVCGTSRQLAPVLGYLGSRQKHSLPDYPYDVPDYAGYPYDVPDYAGYPYDVPDY
AMDIADLRTLGYSQQQQEKIKPKVRSTVAQHHEALVGHGFTHAHIVALSQHPAALGTVAVK
YQDMIAALPEATHEAIVGVGKQWSGARALEALLTVAGELRGPPLQLDTGQLLKIAKRGGVT
AVEAVHAWRNALTGAPLNLTPQQVVAIASNIGGKQALETVQRLLPVLCQAHGLTPDQVVAIA
SNNGGKQALETVQRLLPVLCQAHGLTPEQVVAIASNNGGKQALETVQRLLPVLCQAHGLTP
EQVVAIASHDGGKQALETVQRLLPVLCQAHGLTPAQVVAIASNGGGKQALETVQRLLPVLC
QAHGLTPEQVVAIASNNGGKQALETVQRLLPVLCQAHGLTPEQVVAIASNIGGKQALETVQ
RLLPVLCQAHGLTPDQVVAIASNIGGKQALETVQRLLPVLCQAHGLTPDQVVAIASNGGGK
QALETVQRLLPVLCQAHGLTPEQVVAIASNGGGKQALETVQRLLPVLCQAHGLTPEQVVAI
ASHDGGKQALETVQRLLPVLCQAHGLTPAQVVAIASHDGGKQALETVQRLLPVLCQAHGLT
PAQVVAIASNIGGKQALETVQRLLPVLCQAHGLTPDQVVAIASNNGGRPALESIVAQLSRPD
PALAALTNDHLVALACLGGRPALDAVKKGLGGSPTPYPNYANAGHVEGQSALFMRDNGIS
EGLVFHNNPEGTCGFCVNMTETLLPENAKMTVVPPEGAIPVKRGATGETKVFTGNSNSPK
SPTKGGCSGGSTNLSDIIEKETGKQLVIQESILMLPEEVEEVIGNKPESDILVHTAYDESTDE
NVMLLTSDAPEYKPWALVIQDSNGENKIKML*

- **DdCBE (H) – Nd2-H2 - G1333-C:**

MALSRAVCGTSRQLAPVLGYLGSRQKHSLPDYPYDVPDYAGYPYDVPDYAGYPYDVPDY
AMDIADLRTLGYSQQQQEKIKPKVRSTVAQHHEALVGHGFTHAHIVALSQHPAALGTVAVK
YQDMIAALPEATHEAIVGVGKQWSGARALEALLTVAGELRGPPLQLDTGQLLKIAKRGGVT
AVEAVHAWRNALTGAPLNLTPQQVVAIASNIGGKQALETVQRLLPVLCQAHGLTPDQVVAIA
SNNGGKQALETVQRLLPVLCQAHGLTPEQVVAIASNGGGKQALETVQRLLPVLCQAHGLT
PEQVVAIASNIGGKQALETVQRLLPVLCQAHGLTPDQVVAIASNNGGKQALETVQRLLPVLC
QAHGLTPEQVVAIASNNGGKQALETVQRLLPVLCQAHGLTPEQVVAIASHDGGKQALETVQ
RLLPVLCQAHGLTPAQVVAIASNGGGKQALETVQRLLPVLCQAHGLTPEQVVAIASNNGGK
QALETVQRLLPVLCQAHGLTPEQVVAIASNIGGKQALETVQRLLPVLCQAHGLTPDQVVAIA
SNIGGKQALETVQRLLPVLCQAHGLTPDQVVAIASNGGGKQALETVQRLLPVLCQAHGLTP

EQVVAIASNGGGKQALETVQRLLPVLCQAHGLTPEQVVAIASHDGGKQALETVQRLLPVLCQAHGLTPAQVVAIASHDGGRPALESIVAQLSRPDPALAALTNDHLVALACLGGRPALDAVKKGLGGSPTPYPNYANAGHVEGQSALFMRDNGISEGLVFHNNPEGTCGFCVNMTETLLPENAKMTVVPPEGAIPVKRGATGETKVFTGNSNSPKSPTKGGCSGGSTNLSDIIEKETGKQLVIQESILMLPEEVEEVIGNKPESDILVHTAYDESTDENVMLLTSDAPEYKPWALVIQDSNGENKIKML*

- **DdCBE (H) – Nd3-H1 - G1333-C:**

MALSRAVCGTSRQLAPVLGYLGSRQKHSLPDYPYDVPDYAGYPYDVPDYAGYPYDVPDYAMDIADLRTLGYSQQQQEKIKPKVRSTVAQHHEALVGHGFTHAHIVALSQHPAALGTVAVKYQDMIAALPEATHEAIVGVGKQWSGARALEALLTVAGELRGPPLQLDTGQLLKIAKRGGVTAVEAVHAWRNALTGAPLNLTPQQVVAIASNIGGKQALETVQRLLPVLCQAHGLTPDQVVAIASHDGGKQALETVQRLLPVLCQAHGLTPAQVVAIASNIGGKQALETVQRLLPVLCQAHGLTPDQVVAIASNNGGKQALETVQRLLPVLCQAHGLTPEQVVAIASNIGGKQALETVQRLLPVLCQAHGLTPDQVVAIASNGGGKQALETVQRLLPVLCQAHGLTPEQVVAIASNGGGKQALETVQRLLPVLCQAHGLTPEQVVAIASNGGGKQALETVQRLLPVLCQAHGLTPEQVVAIASNIGGKQALETVQRLLPVLCQAHGLTPDQVVAIASNGGGKQALETVQRLLPVLCQAHGLTPEQVVAIASNGGGKQALETVQRLLPVLCQAHGLTPEQVVAIASNGGGKQALETVQRLLPVLCQAHGLTPEQVVAIASNNGGRPALESIVAQLSRPDPALAALTNDHLVALACLGGRPALDAVKKGLGGSPTPYPNYANAGHVEGQSALFMRDNGISEGLVFHNNPEGTCGFCVNMTETLLPENAKMTVVPPEGAIPVKRGATGETKVFTGNSNSPKSPTKGGCSGGSTNLSDIIEKETGKQLVIQESILMLPEEVEEVIGNKPESDILVHTAYDESTDENVMLLTSDAPEYKPWALVIQDSNGENKIKML*

- **DdCBE (H) – Nd3-H2 - G1333-C:**

MALSRAVCGTSRQLAPVLGYLGSRQKHSLPDYPYDVPDYAGYPYDVPDYAGYPYDVPDYAMDIADLRTLGYSQQQQEKIKPKVRSTVAQHHEALVGHGFTHAHIVALSQHPAALGTVAVKYQDMIAALPEATHEAIVGVGKQWSGARALEALLTVAGELRGPPLQLDTGQLLKIAKRGGVTAVEAVHAWRNALTGAPLNLTPQQVVAIASNNGGKQALETVQRLLPVLCQAHGLTPEQVVAIASNIGGKQALETVQRLLPVLCQAHGLTPDQVVAIASNNGGKQALETVQRLLPVLCQAHGLTPEQVVAIASNGGGKQALETVQRLLPVLCQAHGLTPEQVVAIASNIGGKQALETVQRLLPVLCQAHGLTPDQVVAIASHDGGKQALETVQRLLPVLCQAHGLTPAQVVAIASNIGGKQALETVQRLLPVLCQAHGLTPDQVVAIASNNGGKQALETVQRLLPVLCQAHGLTPEQVVAIASNIGGKQALETVQRLLPVLCQAHGLTPDQVVAIASNGGGKQALETVQRLLPVLCQAHGLTPEQVVAIASNGGGKQALETVQRLLPVLCQAHGLTPEQVVAIASNGGGKQALETVQRLLPVLCQAHGLTPEQVVAIASNIGGKQALETVQRLLPVLCQAHGLTPDQVVAIASNGGGKQALETVQRLLPVLCQAHGLTPEQVVAIASNGGGKQALETVQRLLPVLCQAHGLTPEQVVAIASNGGGRPALESIVAQLSRPDPALAALTNDHLVALACLGGRPALDAVKKGLGGSPTPYPNYANAGHVEGQSALFMRDNGISEGLVFHNNPEGTCGFCVNMTETLLPENAKMTVVPPEGAIPVKRGATGETKVFTGNSNSPKSPTKGGCSGGSTNLSDIIEKETGKQLVIQESILMLPEEVEEVIGNKPESDILVHTAYDESTDENVMLLTSDAPEYKPWALVIQDSNGENKIKML*

- **DdCBE (H) – Nd4-H1 - G1333-C:**

MALSRAVCGTSRQLAPVLGYLGSRQKHSLPDYPYDVPDYAGYPYDVPDYAGYPYDVPDYAMDIADLRTLGYSQQQQEKIKPKVRSTVAQHHEALVGHGFTHAHIVALSQHPAALGTVAVKYQDMIAALPEATHEAIVGVGKQWSGARALEALLTVAGELRGPPLQLDTGQLLKIAKRGGVTAVEAVHAWRNALTGAPLNLTPQQVVAIASNGGGKQALETVQRLLPVLCQAHGLTPEQVVAIASNGGGKQALETVQRLLPVLCQAHGLTPEQVVAIASNGGGKQALETVQRLLPVLCQAHGLTPEQVVAIASNGGGKQALETVQRLLPVLCQAHGLTPEQVVAIASNGGGKQALETVQRLLPVLCQAHGLTPEQVVAIASNGGGKQALETVQRLLPVLCQAHGLTPEQVVAIASNIGGKQALETVQRLLPVLCQAHGLTPDQVVAIASNNGGKQALETVQRLLPVLCQAHGLTPEQVVAIASNNGGKQALETVQRLLPVLCQAHGLTPEQVVAIASNNGGKQALETVQRLLPVLCQAHGLTPEQVV

AIASHDGGKQALETVQRLLPVLCQAHGLTPAQVVAIASNGGGKQALETVQRLLPVLCQAHG
LTPEQVVAIASNGGGRPALESIVAQLSRPDPALAALTNDHLVALACLGGRPALDAVKKGLG
GSPTPYPNYANAGHVEGQSALFMRDNGISEGLVFHNNPEGTCGFCVNMTETLLPENAKMT
VVPPEGAIPVKRGATGETKVFTGNSNSPKSPTKGGCSGGSTNLSDIIEKETGKQLVIQESIL
MLPEEVEEVIGNKPESDILVHTAYDESTDENVMLLTSDAPEYKPWALVIQDSNGENKIKML*

- **DdCBE (H) – Nd4-H2 - G1333-C:**

MALSRAVCGTSRQLAPVLGYLGSRQKHSLPDYPYDVPDYAGYPYDVPDYAGYPYDVPDY
AMDIADLRTLGYSQQQQEKIKPKVRSTVAQHHEALVGHGFTHAHIVALSQHPAALGTVAVK
YQDMIAALPEATHEAIVGVGKQWSGARALEALLTVAGELRGPPLQLDTGQLLKIAKRGGVT
AVEAVHAWRNALTGAPLNLTPQQVVAIASHDGGKQALETVQRLLPVLCQAHGLTPAQVVAI
ASNIGGKQALETVQRLLPVLCQAHGLTPDQVVAIASNNGGKQALETVQRLLPVLCQAHGLT
PEQVVAIASNNGGKQALETVQRLLPVLCQAHGLTPEQVVAIASNGGGKQALETVQRLLPVL
CQAHGLTPEQVVAIASNGGGKQALETVQRLLPVLCQAHGLTPEQVVAIASNGGGKQALETV
QRLLPVLCQAHGLTPEQVVAIASNGGGKQALETVQRLLPVLCQAHGLTPEQVVAIASNGGG
KQALETVQRLLPVLCQAHGLTPEQVVAIASNGGGKQALETVQRLLPVLCQAHGLTPEQVVA
IASNGGGKQALETVQRLLPVLCQAHGLTPEQVVAIASNIGGKQALETVQRLLPVLCQAHGLT
PDQVVAIASNNGGKQALETVQRLLPVLCQAHGLTPEQVVAIASNNGGKQALETVQRLLPVL
CQAHGLTPEQVVAIASNNGGKQALETVQRLLPVLCQAHGLTPEQVVAIASHDGGRPALESI
VAQLSRPDPALAALTNDHLVALACLGGRPALDAVKKGLGGSPTPYPNYANAGHVEGQSAL
FMRDNGISEGLVFHNNPEGTCGFCVNMTETLLPENAKMTVVPPEGAIPVKRGATGETKVFT
GNSNSPKSPTKGGCSGGSTNLSDIIEKETGKQLVIQESILMLPEEVEEVIGNKPESDILVHTA
YDESTDENVMLLTSDAPEYKPWALVIQDSNGENKIKML*

- **DdCBE (H) – Nd4l-H1 - G1333-C:**

MALSRAVCGTSRQLAPVLGYLGSRQKHSLPDYPYDVPDYAGYPYDVPDYAGYPYDVPDY
AMDIADLRTLGYSQQQQEKIKPKVRSTVAQHHEALVGHGFTHAHIVALSQHPAALGTVAVK
YQDMIAALPEATHEAIVGVGKQWSGARALEALLTVAGELRGPPLQLDTGQLLKIAKRGGVT
AVEAVHAWRNALTGAPLNLTPQQVVAIASNGGGKQALETVQRLLPVLCQAHGLTPEQVVAI
ASNNGGKQALETVQRLLPVLCQAHGLTPEQVVAIASNGGGKQALETVQRLLPVLCQAHGL
TPEQVVAIASNIGGKQALETVQRLLPVLCQAHGLTPDQVVAIASNNGGKQALETVQRLLPVL
CQAHGLTPEQVVAIASNGGGKQALETVQRLLPVLCQAHGLTPEQVVAIASNIGGKQALETV
QRLLPVLCQAHGLTPDQVVAIASNNGGKQALETVQRLLPVLCQAHGLTPEQVVAIASNNGG
KQALETVQRLLPVLCQAHGLTPEQVVAIASNGGGKQALETVQRLLPVLCQAHGLTPEQVVA
IASNGGGKQALETVQRLLPVLCQAHGLTPEQVVAIASNNGGKQALETVQRLLPVLCQAHGL
TPEQVVAIASNIGGRPALESIVAQLSRPDPALAALTNDHLVALACLGGRPALDAVKKGLGGS
PTPYPNYANAGHVEGQSALFMRDNGISEGLVFHNNPEGTCGFCVNMTETLLPENAKMTVV
PPEGAIPVKRGATGETKVFTGNSNSPKSPTKGGCSGGSTNLSDIIEKETGKQLVIQESILMLP
EEVEEVIGNKPESDILVHTAYDESTDENVMLLTSDAPEYKPWALVIQDSNGENKIKML*

- **DdCBE (H) – Nd4l-H2 - G1333-C:**

MALSRAVCGTSRQLAPVLGYLGSRQKHSLPDYPYDVPDYAGYPYDVPDYAGYPYDVPDY
AMDIADLRTLGYSQQQQEKIKPKVRSTVAQHHEALVGHGFTHAHIVALSQHPAALGTVAVK
YQDMIAALPEATHEAIVGVGKQWSGARALEALLTVAGELRGPPLQLDTGQLLKIAKRGGVT
AVEAVHAWRNALTGAPLNLTPQQVVAIASNIGGKQALETVQRLLPVLCQAHGLTPDQVVAIA
SNNGGKQALETVQRLLPVLCQAHGLTPEQVVAIASHDGGKQALETVQRLLPVLCQAHGLTP
AQVVAIASNIGGKQALETVQRLLPVLCQAHGLTPDQVVAIASNGGGKQALETVQRLLPVLC
QAHGLTPEQVVAIASNGGGKQALETVQRLLPVLCQAHGLTPEQVVAIASNNGGKQALETVQ
RLLPVLCQAHGLTPEQVVAIASNGGGKQALETVQRLLPVLCQAHGLTPEQVVAIASNIGGK
QALETVQRLLPVLCQAHGLTPDQVVAIASNNGGKQALETVQRLLPVLCQAHGLTPEQVVAI
ASNGGGKQALETVQRLLPVLCQAHGLTPEQVVAIASNIGGKQALETVQRLLPVLCQAHGLT

PDQVVAIASNNGGKQALETVQRLLPVLCQAHGLTPEQVVAIASNNGGKQALETVQRLLPVL
CQAHGLTPEQVVAIASNGGGKQALETVQRLLPVLCQAHGLTPEQVVAIASNGGGRPALESI
VAQLSRPDPALAALTNDHLVALACLGGRPALDAVKKGLGGSPTPYPNYANAGHVEGQSAL
FMRDNGISEGLVFHNNPEGTCGFCVNMTETLLPENAKMTVVPPEGAIPVKRGATGETKVFT
GNSNSPKSPTKGGCSGGSTNLSDIIEKETGKQLVIQESILMLPEEVEEVIGNKPESDILVHTA
YDESTDENVMLLTSDAPEYKPWALVIQDSNGENKIKML*

- **DdCBE (H) – Nd5-H1 - G1333-C:**

MALSRAVCGTSRQLAPVLGYLGSRQKHSLPDYPYDVPDYAGYPYDVPDYAGYPYDVPDY
AMDIADLRTLGYSQQQQEKIKPKVRSTVAQHHEALVGHGFTHAHIVALSQHPAALGTVAVK
YQDMIAALPEATHEAIVGVGKQWSGARALEALLTVAGELRGPPLQLDTGQLLKIAKRGGVT
AVEAVHAWRNALTGAPLNLTPQQVVAIASHDGGKQALETVQRLLPVLCQAHGLTPAQVVAI
ASNGGGKQALETVQRLLPVLCQAHGLTPEQVVAIASNIGGKQALETVQRLLPVLCQAHGLT
PDQVVAIASNGGGKQALETVQRLLPVLCQAHGLTPEQVVAIASNGGGKQALETVQRLLPVL
CQAHGLTPEQVVAIASNNGGKQALETVQRLLPVLCQAHGLTPEQVVAIASNIGGKQALETV
QRLLPVLCQAHGLTPDQVVAIASNIGGKQALETVQRLLPVLCQAHGLTPDQVVAIASNGGG
KQALETVQRLLPVLCQAHGLTPEQVVAIASNGGGKQALETVQRLLPVLCQAHGLTPEQVVA
IASNGGGKQALETVQRLLPVLCQAHGLTPEQVVAIASNIGGKQALETVQRLLPVLCQAHGLT
PDQVVAIASNGGGRPALESIVAQLSRPDPALAALTNDHLVALACLGGRPALDAVKKGLGGS
PTPYPNYANAGHVEGQSALFMRDNGISEGLVFHNNPEGTCGFCVNMTETLLPENAKMTVV
PPEGAIPVKRGATGETKVFTGNSNSPKSPTKGGCSGGSTNLSDIIEKETGKQLVIQESILMLP
EEVEEVIGNKPESDILVHTAYDESTDENVMLLTSDAPEYKPWALVIQDSNGENKIKML*

- **DdCBE (H) – Nd5-H2 - G1333-C:**

MALSRAVCGTSRQLAPVLGYLGSRQKHSLPDYPYDVPDYAGYPYDVPDYAGYPYDVPDY
AMDIADLRTLGYSQQQQEKIKPKVRSTVAQHHEALVGHGFTHAHIVALSQHPAALGTVAVK
YQDMIAALPEATHEAIVGVGKQWSGARALEALLTVAGELRGPPLQLDTGQLLKIAKRGGVT
AVEAVHAWRNALTGAPLNLTPQQVVAIASNIGGKQALETVQRLLPVLCQAHGLTPDQVVAIA
SNIGGKQALETVQRLLPVLCQAHGLTPDQVVAIASNNGGKQALETVQRLLPVLCQAHGLTP
EQVVAIASNGGGKQALETVQRLLPVLCQAHGLTPEQVVAIASNGGGKQALETVQRLLPVLC
QAHGLTPEQVVAIASHDGGKQALETVQRLLPVLCQAHGLTPAQVVAIASNGGGKQALETVQ
RLLPVLCQAHGLTPEQVVAIASNIGGKQALETVQRLLPVLCQAHGLTPDQVVAIASNGGGK
QALETVQRLLPVLCQAHGLTPEQVVAIASNGGGKQALETVQRLLPVLCQAHGLTPEQVVAI
ASNNGGKQALETVQRLLPVLCQAHGLTPEQVVAIASNIGGKQALETVQRLLPVLCQAHGLT
PDQVVAIASNIGGKQALETVQRLLPVLCQAHGLTPDQVVAIASNGGGKQALETVQRLLPVLC
QAHGLTPEQVVAIASNGGGKQALETVQRLLPVLCQAHGLTPEQVVAIASNGGGRPALESIV
AQLSRPDPALAALTNDHLVALACLGGRPALDAVKKGLGGSPTPYPNYANAGHVEGQSALF
MRDNGISEGLVFHNNPEGTCGFCVNMTETLLPENAKMTVVPPEGAIPVKRGATGETKVFT
GNSNSPKSPTKGGCSGGSTNLSDIIEKETGKQLVIQESILMLPEEVEEVIGNKPESDILVHTA
YDESTDENVMLLTSDAPEYKPWALVIQDSNGENKIKML*

- **DdCBE (H) – Nd6-H1 - G1333-C:**

MALSRAVCGTSRQLAPVLGYLGSRQKHSLPDYPYDVPDYAGYPYDVPDYAGYPYDVPDY
AMDIADLRTLGYSQQQQEKIKPKVRSTVAQHHEALVGHGFTHAHIVALSQHPAALGTVAVK
YQDMIAALPEATHEAIVGVGKQWSGARALEALLTVAGELRGPPLQLDTGQLLKIAKRGGVT
AVEAVHAWRNALTGAPLNLTPQQVVAIASHDGGKQALETVQRLLPVLCQAHGLTPAQVVAI
ASHDGGKQALETVQRLLPVLCQAHGLTPAQVVAIASNIGGKQALETVQRLLPVLCQAHGLT
PDQVVAIASNNGGKQALETVQRLLPVLCQAHGLTPEQVVAIASNIGGKQALETVQRLLPVLC
QAHGLTPDQVVAIASNNGGKQALETVQRLLPVLCQAHGLTPEQVVAIASNIGGKQALETVQ
RLLPVLCQAHGLTPDQVVAIASHDGGKQALETVQRLLPVLCQAHGLTPAQVVAIASNGGGK
QALETVQRLLPVLCQAHGLTPEQVVAIASNGGGKQALETVQRLLPVLCQAHGLTPEQVVAI

ASNNGGKQALETVQRLLPVLCQAHGLTPEQVVAIASNNGGKQALETVQRLLPVLCQAHGLT
PEQVVAIASNNGGKQALETVQRLLPVLCQAHGLTPEQVVAIASNNGGRPALESIVAQLSRP
DPALAALTNDHLVALACLGGRPALDAVKKGLGGSPTPYPNYANAGHVEGQSALFMRDNGI
SEGLVFHNNPEGTCGFCVNMTETLLPENAKMTVVPPEGAIPVKRGATGETKVFTGNSNSP
KSPTKGGCSGGSTNLSDIIEKETGKQLVIQESILMLPEEVEEVIGNKPESDILVHTAYDESTD
ENVMLLTSDAPEYKPWALVIQDSNGENKIKML*

- **DdCBE (H) – Nd6-H2 - G1333-C:**

MALSRAVCGTSRQLAPVLGYLGSRQKHSLPDYPYDVPDYAGYPYDVPDYAGYPYDVPDY
AMDIADLRTLGYSQQQQEKIKPKVRSTVAQHHEALVGHGFTHAHIVALSQHPAALGTVAVK
YQDMIAALPEATHEAIVGVGKQWSGARALEALLTVAGELRGPPLQLDTGQLLKIAKRGGVT
AVEAVHAWRNALTGAPLNLTPQQVVAIASNIGGKQALETVQRLLPVLCQAHGLTPDQVVAIA
SNGGGKQALETVQRLLPVLCQAHGLTPEQVVAIASHDGGKQALETVQRLLPVLCQAHGLT
PAQVVAIASHDGGKQALETVQRLLPVLCQAHGLTPAQVVAIASNIGGKQALETVQRLLPVLC
QAHGLTPDQVVAIASNNGGKQALETVQRLLPVLCQAHGLTPEQVVAIASNIGGKQALETVQ
RLLPVLCQAHGLTPDQVVAIASNNGGKQALETVQRLLPVLCQAHGLTPEQVVAIASNIGGK
QALETVQRLLPVLCQAHGLTPDQVVAIASHDGGKQALETVQRLLPVLCQAHGLTPAQVVAI
ASNGGGKQALETVQRLLPVLCQAHGLTPEQVVAIASNGGGKQALETVQRLLPVLCQAHGL
TPEQVVAIASNNGGKQALETVQRLLPVLCQAHGLTPEQVVAIASNNGGKQALETVQRLLPV
LCQAHGLTPEQVVAIASNNGGKQALETVQRLLPVLCQAHGLTPEQVVAIASNNGGKQALET
VQRLLPVLCQAHGLTPEQVVAIASNIGGRPALESIVAQLSRPDPALAALTNDHLVALACLGG
RPALDAVKKGLGGSPTPYPNYANAGHVEGQSALFMRDNGISEGLVFHNNPEGTCGFCVN
MTETLLPENAKMTVVPPEGAIPVKRGATGETKVFTGNSNSPKSPTKGGCSGGSTNLSDIIEK
ETGKQLVIQESILMLPEEVEEVIGNKPESDILVHTAYDESTDENVMLLTSDAPEYKPWALVIQ
DSNGENKIKML*

- **DdCBE (H) – Cytb-H1 - G1333-C:**

MALSRAVCGTSRQLAPVLGYLGSRQKHSLPDYPYDVPDYAGYPYDVPDYAGYPYDVPDY
AMDIADLRTLGYSQQQQEKIKPKVRSTVAQHHEALVGHGFTHAHIVALSQHPAALGTVAVK
YQDMIAALPEATHEAIVGVGKQWSGARALEALLTVAGELRGPPLQLDTGQLLKIAKRGGVT
AVEAVHAWRNALTGAPLNLTPQQVVAIASNNGGKQALETVQRLLPVLCQAHGLTPEQVVAI
ASHDGGKQALETVQRLLPVLCQAHGLTPAQVVAIASNNGGKQALETVQRLLPVLCQAHGLT
PEQVVAIASNGGGKQALETVQRLLPVLCQAHGLTPEQVVAIASNNGGKQALETVQRLLPVL
CQAHGLTPEQVVAIASNGGGKQALETVQRLLPVLCQAHGLTPEQVVAIASNIGGKQALETV
QRLLPVLCQAHGLTPDQVVAIASNGGGKQALETVQRLLPVLCQAHGLTPEQVVAIASNIGG
KQALETVQRLLPVLCQAHGLTPDQVVAIASNGGGKQALETVQRLLPVLCQAHGLTPEQVVA
IASNIGGKQALETVQRLLPVLCQAHGLTPDQVVAIASNGGGKQALETVQRLLPVLCQAHGLT
PEQVVAIASHDGGKQALETVQRLLPVLCQAHGLTPAQVVAIASNNGGRPALESIVAQLSRP
DPALAALTNDHLVALACLGGRPALDAVKKGLGGSPTPYPNYANAGHVEGQSALFMRDNGI
SEGLVFHNNPEGTCGFCVNMTETLLPENAKMTVVPPEGAIPVKRGATGETKVFTGNSNSP
KSPTKGGCSGGSTNLSDIIEKETGKQLVIQESILMLPEEVEEVIGNKPESDILVHTAYDESTD
ENVMLLTSDAPEYKPWALVIQDSNGENKIKML*

- **DdCBE (H) – Cytb-H2 - G1333-C:**

MALSRAVCGTSRQLAPVLGYLGSRQKHSLPDYPYDVPDYAGYPYDVPDYAGYPYDVPDY
AMDIADLRTLGYSQQQQEKIKPKVRSTVAQHHEALVGHGFTHAHIVALSQHPAALGTVAVK
YQDMIAALPEATHEAIVGVGKQWSGARALEALLTVAGELRGPPLQLDTGQLLKIAKRGGVT
AVEAVHAWRNALTGAPLNLTPQQVVAIASNGGGKQALETVQRLLPVLCQAHGLTPEQVVAI
ASNGGGKQALETVQRLLPVLCQAHGLTPEQVVAIASNNGGKQALETVQRLLPVLCQAHGL
TPEQVVAIASHDGGKQALETVQRLLPVLCQAHGLTPAQVVAIASNNGGKQALETVQRLLPV
LCQAHGLTPEQVVAIASNGGGKQALETVQRLLPVLCQAHGLTPEQVVAIASNNGGKQALET

VQRLLPVLCQAHGLTPEQVVAIASNGGGKQALETVQRLLPVLCQAHGLTPEQVVAIASNIG
GKQALETVQRLLPVLCQAHGLTPDQVVAIASNGGGKQALETVQRLLPVLCQAHGLTPEQVV
AIASNIGGKQALETVQRLLPVLCQAHGLTPDQVVAIASNGGGKQALETVQRLLPVLCQAHGL
TPEQVVAIASNIGGKQALETVQRLLPVLCQAHGLTPDQVVAIASNGGGRPALESIVAQLSRP
DPALAALTNDHLVALACLGGRPALDAVKKGLGGSPTPYPNYANAGHVEGQSALFMRDNGI
SEGLVFHNNPEGTCGFCVNMTETLLPENAKMTVVPPEGAIPVKRGATGETKVFTGNSNSP
KSPTKGGCSGGSTNLSDIIEKETGKQLVIQESILMLPEEVEEVIGNKPESDILVHTAYDESTD
ENVMLLTSDAPEYKPWALVIQDSNGENKIKML*

- **DdCBE (H) – Col-H1 - G1333-C:**

MALSRAVCGTSRQLAPVLGYLGSRQKHSLPDYPYDVPDYAGYPYDVPDYAGYPYDVPDY
AMDIADLRTLGYSQQQQEKIKPKVRSTVAQHHEALVGHGFTHAHIVALSQHPAALGTVAVK
YQDMIAALPEATHEAIVGVGKQWSGARALEALLTVAGELRGPPLQLDTGQLLKIAKRGGVT
AVEAVHAWRNALTGAPLNLTPQQVVAIASNGGGKQALETVQRLLPVLCQAHGLTPEQVVAI
ASNNGGKQALETVQRLLPVLCQAHGLTPEQVVAIASNGGGKQALETVQRLLPVLCQAHGL
TPEQVVAIASNNGGKQALETVQRLLPVLCQAHGLTPEQVVAIASNIGGKQALETVQRLLPVL
CQAHGLTPDQVVAIASNGGGKQALETVQRLLPVLCQAHGLTPEQVVAIASNGGGKQALET
VQRLLPVLCQAHGLTPEQVVAIASNNGGKQALETVQRLLPVLCQAHGLTPEQVVAIASNNG
GKQALETVQRLLPVLCQAHGLTPEQVVAIASNGGGKQALETVQRLLPVLCQAHGLTPEQVV
AIASNGGGKQALETVQRLLPVLCQAHGLTPEQVVAIASNNGGKQALETVQRLLPVLCQAHG
LTPEQVVAIASNIGGRPALESIVAQLSRPDPALAALTNDHLVALACLGGRPALDAVKKGLGG
SPTPYPNYANAGHVEGQSALFMRDNGISEGLVFHNNPEGTCGFCVNMTETLLPENAKMTV
VPPEGAIPVKRGATGETKVFTGNSNSPKSPTKGGCSGGSTNLSDIIEKETGKQLVIQESILML
PEEVEEVIGNKPESDILVHTAYDESTDENVMLLTSDAPEYKPWALVIQDSNGENKIKML*

- **DdCBE (H) – Col-H2 - G1333-C:**

MALSRAVCGTSRQLAPVLGYLGSRQKHSLPDYPYDVPDYAGYPYDVPDYAGYPYDVPDY
AMDIADLRTLGYSQQQQEKIKPKVRSTVAQHHEALVGHGFTHAHIVALSQHPAALGTVAVK
YQDMIAALPEATHEAIVGVGKQWSGARALEALLTVAGELRGPPLQLDTGQLLKIAKRGGVT
AVEAVHAWRNALTGAPLNLTPQQVVAIASNIGGKQALETVQRLLPVLCQAHGLTPDQVVAIA
SNGGGKQALETVQRLLPVLCQAHGLTPEQVVAIASHDGGKQALETVQRLLPVLCQAHGLT
PAQVVAIASNGGGKQALETVQRLLPVLCQAHGLTPEQVVAIASNGGGKQALETVQRLLPVL
CQAHGLTPEQVVAIASNGGGKQALETVQRLLPVLCQAHGLTPEQVVAIASNNGGKQALETV
QRLLPVLCQAHGLTPEQVVAIASNGGGKQALETVQRLLPVLCQAHGLTPEQVVAIASNNGG
KQALETVQRLLPVLCQAHGLTPEQVVAIASNIGGKQALETVQRLLPVLCQAHGLTPDQVVAI
ASNGGGKQALETVQRLLPVLCQAHGLTPEQVVAIASNGGGKQALETVQRLLPVLCQAHGL
TPEQVVAIASNNGGKQALETVQRLLPVLCQAHGLTPEQVVAIASNNGGKQALETVQRLLPV
LCQAHGLTPEQVVAIASNGGGKQALETVQRLLPVLCQAHGLTPEQVVAIASNGGGRPALES
IVAQLSRPDPALAALTNDHLVALACLGGRPALDAVKKGLGGSPTPYPNYANAGHVEGQSAL
FMRDNGISEGLVFHNNPEGTCGFCVNMTETLLPENAKMTVVPPEGAIPVKRGATGETKVFT
GNSNSPKSPTKGGCSGGSTNLSDIIEKETGKQLVIQESILMLPEEVEEVIGNKPESDILVHTA
YDESTDENVMLLTSDAPEYKPWALVIQDSNGENKIKML*

- **DdCBE (H) – ColI-H1 - G1333-C:**

MALSRAVCGTSRQLAPVLGYLGSRQKHSLPDYPYDVPDYAGYPYDVPDYAGYPYDVPDY
AMDIADLRTLGYSQQQQEKIKPKVRSTVAQHHEALVGHGFTHAHIVALSQHPAALGTVAVK
YQDMIAALPEATHEAIVGVGKQWSGARALEALLTVAGELRGPPLQLDTGQLLKIAKRGGVT
AVEAVHAWRNALTGAPLNLTPQQVVAIASNIGGKQALETVQRLLPVLCQAHGLTPDQVVAIA
SNGGGKQALETVQRLLPVLCQAHGLTPEQVVAIASNIGGKQALETVQRLLPVLCQAHGLTP
DQVVAIASNGGGKQALETVQRLLPVLCQAHGLTPEQVVAIASNGGGKQALETVQRLLPVLC

QAHGLTPEQVVAIASHDGGKQALETVQRLLPVLCQAHGLTPAQVVAIASNNGGKQALETVQ
RLLPVLCQAHGLTPEQVVAIASNGGGKQALETVQRLLPVLCQAHGLTPEQVVAIASNIGGK
QALETVQRLLPVLCQAHGLTPDQVVAIASNNGGKQALETVQRLLPVLCQAHGLTPEQVVAI
ASHDGGKQALETVQRLLPVLCQAHGLTPAQVVAIASNGGGKQALETVQRLLPVLCQAHGL
TPEQVVAIASNGGGRPALESIVAQLSRPDPALAALTNDHLVALACLGGRPALDAVKKGLGG
SPTPYPNYANAGHVEGQSALFMRDNGISEGLVFHNNPEGTCGFCVNMTETLLPENAKMTV
VPPEGAIPVKRGATGETKVFTGNSNSPKSPTKGGCSGGSTNLSDIIEKETGKQLVIQESILML
PEEVEEVIGNKPESDILVHTAYDESTDENVMLLTSDAPEYKPWALVIQDSNGENKIKML*

- **DdCBE (H) – ColI-H2 - G1333-C:**

MALSRAVCGTSRQLAPVLGYLGSRQKHSLPDYPYDVPDYAGYPYDVPDYAGYPYDVPDY
AMDIADLRTLGYSQQQQEKIKPKVRSTVAQHHEALVGHGFTHAHIVALSQHPAALGTVAVK
YQDMIAALPEATHEAIVGVGKQWSGARALEALLTVAGELRGPPLQLDTGQLLKIAKRGGVT
AVEAVHAWRNALTGAPLNLTPQQVVAIASHDGGKQALETVQRLLPVLCQAHGLTPAQVVAI
ASNIGGKQALETVQRLLPVLCQAHGLTPDQVVAIASNNGGKQALETVQRLLPVLCQAHGLT
PEQVVAIASNGGGKQALETVQRLLPVLCQAHGLTPEQVVAIASNIGGKQALETVQRLLPVLC
QAHGLTPDQVVAIASNGGGKQALETVQRLLPVLCQAHGLTPEQVVAIASNIGGKQALETVQ
RLLPVLCQAHGLTPDQVVAIASNGGGKQALETVQRLLPVLCQAHGLTPEQVVAIASNGGGK
QALETVQRLLPVLCQAHGLTPEQVVAIASHDGGKQALETVQRLLPVLCQAHGLTPAQVVAI
ASNNGGKQALETVQRLLPVLCQAHGLTPEQVVAIASNGGGKQALETVQRLLPVLCQAHGL
TPEQVVAIASNIGGKQALETVQRLLPVLCQAHGLTPDQVVAIASNNGGKQALETVQRLLPVL
CQAHGLTPEQVVAIASHDGGRPALESIVAQLSRPDPALAALTNDHLVALACLGGRPALDAV
KKGLGGSPTPYPNYANAGHVEGQSALFMRDNGISEGLVFHNNPEGTCGFCVNMTETLLPE
NAKMTVVPPEGAIPVKRGATGETKVFTGNSNSPKSPTKGGCSGGSTNLSDIIEKETGKQLVI
QESILMLPEEVEEVIGNKPESDILVHTAYDESTDENVMLLTSDAPEYKPWALVIQDSNGENKI
KML*

- **DdCBE (H) – ColII-H1 - G1333-C:**

MALSRAVCGTSRQLAPVLGYLGSRQKHSLPDYPYDVPDYAGYPYDVPDYAGYPYDVPDY
AMDIADLRTLGYSQQQQEKIKPKVRSTVAQHHEALVGHGFTHAHIVALSQHPAALGTVAVK
YQDMIAALPEATHEAIVGVGKQWSGARALEALLTVAGELRGPPLQLDTGQLLKIAKRGGVT
AVEAVHAWRNALTGAPLNLTPQQVVAIASHDGGKQALETVQRLLPVLCQAHGLTPAQVVAI
ASNGGGKQALETVQRLLPVLCQAHGLTPEQVVAIASNIGGKQALETVQRLLPVLCQAHGLT
PDQVVAIASNGGGKQALETVQRLLPVLCQAHGLTPEQVVAIASNIGGKQALETVQRLLPVLC
QAHGLTPDQVVAIASNIGGKQALETVQRLLPVLCQAHGLTPDQVVAIASNNGGKQALETVQ
RLLPVLCQAHGLTPEQVVAIASNNGGKQALETVQRLLPVLCQAHGLTPEQVVAIASHDGGK
QALETVQRLLPVLCQAHGLTPAQVVAIASNGGGKQALETVQRLLPVLCQAHGLTPEQVVAI
ASNIGGKQALETVQRLLPVLCQAHGLTPDQVVAIASNGGGKQALETVQRLLPVLCQAHGLT
PEQVVAIASNNGGRPALESIVAQLSRPDPALAALTNDHLVALACLGGRPALDAVKKGLGGS
PTPYPNYANAGHVEGQSALFMRDNGISEGLVFHNNPEGTCGFCVNMTETLLPENAKMTVV
PPEGAIPVKRGATGETKVFTGNSNSPKSPTKGGCSGGSTNLSDIIEKETGKQLVIQESILMLP
EEVEEVIGNKPESDILVHTAYDESTDENVMLLTSDAPEYKPWALVIQDSNGENKIKML*

- **DdCBE (H) – ColII-H2 - G1333-C:**

MALSRAVCGTSRQLAPVLGYLGSRQKHSLPDYPYDVPDYAGYPYDVPDYAGYPYDVPDY
AMDIADLRTLGYSQQQQEKIKPKVRSTVAQHHEALVGHGFTHAHIVALSQHPAALGTVAVK
YQDMIAALPEATHEAIVGVGKQWSGARALEALLTVAGELRGPPLQLDTGQLLKIAKRGGVT
AVEAVHAWRNALTGAPLNLTPQQVVAIASNGGGKQALETVQRLLPVLCQAHGLTPEQVVAI
ASHDGGKQALETVQRLLPVLCQAHGLTPAQVVAIASNGGGKQALETVQRLLPVLCQAHGL
TPEQVVAIASNIGGKQALETVQRLLPVLCQAHGLTPDQVVAIASNGGGKQALETVQRLLPVL

CQAHGLTPEQVVAIASNIGGKQALETVQRLLPVLCQAHGLTPDQVVAIASNIGGKQALETVQRLLPVLCQAHGLTPDQVVAIASNNGGKQALETVQRLLPVLCQAHGLTPEQVVAIASNNGGKQALETVQRLLPVLCQAHGLTPEQVVAIASHDGGKQALETVQRLLPVLCQAHGLTPAQVVAIASNGGGKQALETVQRLLPVLCQAHGLTPEQVVAIASNIGGKQALETVQRLLPVLCQAHGLTPDQVVAIASNGGGKQALETVQRLLPVLCQAHGLTPEQVVAIASNNGGKQALETVQRLLPVLCQAHGLTPEQVVAIASNIGGKQALETVQRLLPVLCQAHGLTPDQVVAIASNGGGRPALESIVAQLSRPDPALAALTNDHLVALACLGGRPALDAVKKGLGGSPTPYPNYANAGHVEGQSALFMRDNGISEGLVFHNNPEGTCGFCVNMTETLLPENAKMTVVPPEGAIPVKRGATGETKVFTGNSNSPKSPTKGGCSGGSTNLSDIIEKETGKQLVIQESILMLPEEVEEVIGNKPESDILVHTAYDESTDENVMLLTSDAPEYKPWALVIQDSNGENKIKML*

- **DdCBE (H) – Atp6-H1 - G1333-C:**

MALSRAVCGTSRQLAPVLGYLGSRQKHSLPDYPYDVPDYAGYPYDVPDYAGYPYDVPDYAMDIADLRTLGYSQQQQEKIKPKVRSTVAQHHEALVGHGFTHAHIVALSQHPAALGTVAVKYQDMIAALPEATHEAIVGVGKQWSGARALEALLTVAGELRGPPLQLDTGQLLKIAKRGGVTAVEAVHAWRNALTGAPLNLTPQQVVAIASNGGGKQALETVQRLLPVLCQAHGLTPEQVVAIASNNGGKQALETVQRLLPVLCQAHGLTPEQVVAIASNIGGKQALETVQRLLPVLCQAHGLTPDQVVAIASNGGGKQALETVQRLLPVLCQAHGLTPEQVVAIASNIGGKQALETVQRLLPVLCQAHGLTPDQVVAIASNIGGKQALETVQRLLPVLCQAHGLTPDQVVAIASNGGGKQALETVQRLLPVLCQAHGLTPEQVVAIASNIGGKQALETVQRLLPVLCQAHGLTPDQVVAIASNIGGKQALETVQRLLPVLCQAHGLTPDQVVAIASNNGGKQALETVQRLLPVLCQAHGLTPEQVVAIASNGGGKQALETVQRLLPVLCQAHGLTPEQVVAIASNGGGKQALETVQRLLPVLCQAHGLTPEQVVAIASNGGGRPALESIVAQLSRPDPALAALTNDHLVALACLGGRPALDAVKKGLGGSPTPYPNYANAGHVEGQSALFMRDNGISEGLVFHNNPEGTCGFCVNMTETLLPENAKMTVVPPEGAIPVKRGATGETKVFTGNSNSPKSPTKGGCSGGSTNLSDIIEKETGKQLVIQESILMLPEEVEEVIGNKPESDILVHTAYDESTDENVMLLTSDAPEYKPWALVIQDSNGENKIKML*

- **DdCBE (H) – Atp6-H2 - G1333-C:**

MALSRAVCGTSRQLAPVLGYLGSRQKHSLPDYPYDVPDYAGYPYDVPDYAGYPYDVPDYAMDIADLRTLGYSQQQQEKIKPKVRSTVAQHHEALVGHGFTHAHIVALSQHPAALGTVAVKYQDMIAALPEATHEAIVGVGKQWSGARALEALLTVAGELRGPPLQLDTGQLLKIAKRGGVTAVEAVHAWRNALTGAPLNLTPQQVVAIASNGGGKQALETVQRLLPVLCQAHGLTPEQVVAIASNGGGKQALETVQRLLPVLCQAHGLTPEQVVAIASNNGGKQALETVQRLLPVLCQAHGLTPEQVVAIASNGGGKQALETVQRLLPVLCQAHGLTPEQVVAIASNGGGKQALETVQRLLPVLCQAHGLTPEQVVAIASNGGGKQALETVQRLLPVLCQAHGLTPEQVVAIASNNGGKQALETVQRLLPVLCQAHGLTPEQVVAIASNIGGKQALETVQRLLPVLCQAHGLTPDQVVAIASNGGGKQALETVQRLLPVLCQAHGLTPEQVVAIASNIGGKQALETVQRLLPVLCQAHGLTPDQVVAIASNIGGKQALETVQRLLPVLCQAHGLTPDQVVAIASNGGGKQALETVQRLLPVLCQAHGLTPEQVVAIASNIGGKQALETVQRLLPVLCQAHGLTPDQVVAIASNIGGKQALETVQRLLPVLCQAHGLTPDQVVAIASNNGGKQALETVQRLLPVLCQAHGLTPEQVVAIASNGGGRPALESIVAQLSRPDPALAALTNDHLVALACLGGRPALDAVKKGLGGSPTPYPNYANAGHVEGQSALFMRDNGISEGLVFHNNPEGTCGFCVNMTETLLPENAKMTVVPPEGAIPVKRGATGETKVFTGNSNSPKSPTKGGCSGGSTNLSDIIEKETGKQLVIQESILMLPEEVEEVIGNKPESDILVHTAYDESTDENVMLLTSDAPEYKPWALVIQDSNGENKIKML*

- **DdCBE (H) – Atp8-H1 - G1333-C:**

MALSRAVCGTSRQLAPVLGYLGSRQKHSLPDYPYDVPDYAGYPYDVPDYAGYPYDVPDYAMDIADLRTLGYSQQQQEKIKPKVRSTVAQHHEALVGHGFTHAHIVALSQHPAALGTVAVKYQDMIAALPEATHEAIVGVGKQWSGARALEALLTVAGELRGPPLQLDTGQLLKIAKRGGVTAVEAVHAWRNALTGAPLNLTPQQVVAIASNNGGKQALETVQRLLPVLCQAHGLTPEQVVAI

ASNIGGKQALETVQRLLPVLCQAHGLTPDQVVAIASNGGGKQALETVQRLLPVLCQAHGLT
PEQVVAIASNNGGKQALETVQRLLPVLCQAHGLTPEQVVAIASNIGGKQALETVQRLLPVLC
QAHGLTPDQVVAIASNNGGKQALETVQRLLPVLCQAHGLTPEQVVAIASNIGGKQALETVQ
RLLPVLCQAHGLTPDQVVAIASNGGGKQALETVQRLLPVLCQAHGLTPEQVVAIASNIGGK
QALETVQRLLPVLCQAHGLTPDQVVAIASNIGGKQALETVQRLLPVLCQAHGLTPDQVVAIA
SNGGGKQALETVQRLLPVLCQAHGLTPEQVVAIASNGGGKQALETVQRLLPVLCQAHGLT
PEQVVAIASNNGGKQALETVQRLLPVLCQAHGLTPEQVVAIASNGGGRPALESIVAQLSRP
DPALAALTNDHLVALACLGGRPALDAVKKGLGGSPTPYPNYANAGHVEGQSALFMRDNGI
SEGLVFHNNPEGTCGFCVNMTETLLPENAKMTVVPPEGAIPVKRGATGETKVFTGNSNSP
KSPTKGGCSGGSTNLSDIIEKETGKQLVIQESILMLPEEVEEVIGNKPESDILVHTAYDESTD
ENVMLLTSDAPEYKPWALVIQDSNGENKIKML*

- **DdCBE (H) – Atp8-H2 - G1333-C:**

MALSRAVCGTSRQLAPVLGYLGSRQKHSLPDYPYDVPDYAGYPYDVPDYAGYPYDVPDY
AMDIADLRTLGYSQQQQEKIKPKVRSTVAQHHEALVGHGFTHAHIVALSQHPAALGTVAVK
YQDMIAALPEATHEAIVGVGKQWSGARALEALLTVAGELRGPPLQLDTGQLLKIAKRGGVT
AVEAVHAWRNALTGAPLNLTPQQVVAIASNGGGKQALETVQRLLPVLCQAHGLTPEQVVAI
ASNIGGKQALETVQRLLPVLCQAHGLTPDQVVAIASNGGGKQALETVQRLLPVLCQAHGLT
PEQVVAIASNGGGKQALETVQRLLPVLCQAHGLTPEQVVAIASNNGGKQALETVQRLLPVL
CQAHGLTPEQVVAIASNIGGKQALETVQRLLPVLCQAHGLTPDQVVAIASNGGGKQALETV
QRLLPVLCQAHGLTPEQVVAIASNNGGKQALETVQRLLPVLCQAHGLTPEQVVAIASNIGGK
QALETVQRLLPVLCQAHGLTPDQVVAIASNNGGKQALETVQRLLPVLCQAHGLTPEQVVAI
ASNIGGKQALETVQRLLPVLCQAHGLTPDQVVAIASNGGGKQALETVQRLLPVLCQAHGLT
PEQVVAIASNIGGKQALETVQRLLPVLCQAHGLTPDQVVAIASNIGGKQALETVQRLLPVLC
QAHGLTPDQVVAIASNGGGKQALETVQRLLPVLCQAHGLTPEQVVAIASNGGGRPALESIV
AQLSRPDPALAALTNDHLVALACLGGRPALDAVKKGLGGSPTPYPNYANAGHVEGQSALF
MRDNGISEGLVFHNNPEGTCGFCVNMTETLLPENAKMTVVPPEGAIPVKRGATGETKVFT
GNSNSPKSPTKGGCSGGSTNLSDIIEKETGKQLVIQESILMLPEEVEEVIGNKPESDILVHTA
YDESTDENVMLLTSDAPEYKPWALVIQDSNGENKIKML*
