# Supplementary material for: A library of base editors for the precise ablation of all protein-coding genes in the mouse mitochondrial genome
Source: Nat Biomed Eng. 2022 Dec 5;7(5):692–703. doi: 10.1038/s41551-022-00968-1 (PMC10195678; doi:10.1038/s41551-022-00968-1)
Supplement: Supplementary file 2 — Reporting Summary [file 41551_2022_968_MOESM2_ESM.pdf]

nature portfolio

Corresponding author(s): Michal Minczuk

Last updated by author(s): Oct 16, 2022

Reporting Summary

Nature Portfolio wishes to improve the reproducibility of the work that we publish. This form provides structure for consistency and transparency in reporting. For further information on Nature Portfolio policies, see our Editorial Policies and the Editorial Policy Checklist.

Statistics

For all statistical analyses, confirm that the following items are present in the figure legend, table legend, main text, or Methods section.

n/a	Confirmed	
☐	☒	The exact sample size (*n*) for each experimental group/condition, given as a discrete number and unit of measurement
☐	☒	A statement on whether measurements were taken from distinct samples or whether the same sample was measured repeatedly
☐	☒	The statistical test(s) used AND whether they are one- or two-sided<br>*Only common tests should be described solely by name; describe more complex techniques in the Methods section.*
☒	☐	A description of all covariates tested
☒	☐	A description of any assumptions or corrections, such as tests of normality and adjustment for multiple comparisons
☐	☒	A full description of the statistical parameters including central tendency (e.g. means) or other basic estimates (e.g. regression coefficient) AND variation (e.g. standard deviation) or associated estimates of uncertainty (e.g. confidence intervals)
☒	☐	For null hypothesis testing, the test statistic (e.g. *F*, *t*, *r*) with confidence intervals, effect sizes, degrees of freedom and *P* value noted<br>*Give P values as exact values whenever suitable.*
☒	☐	For Bayesian analysis, information on the choice of priors and Markov chain Monte Carlo settings
☒	☐	For hierarchical and complex designs, identification of the appropriate level for tests and full reporting of outcomes
☒	☐	Estimates of effect sizes (e.g. Cohen's *d*, Pearson's *r*), indicating how they were calculated

Our web collection on statistics for biologists contains articles on many of the points above.

Software and code

Policy information about availability of computer code

Data collection	Illumina MiSeq manufacturer's software (version 4.0)
Data analysis	TrimGalore! version 0.6.6 (doi: 10.5281/zenodo.5127899)<br>https://github.com/FelixKrueger/TrimGalore/releases<br>Bowtie version 2 (doi: 10.1038%2Fnmeth.1923)<br>https://github.com/BenLangmead/bowtie2<br>Samtools version 1.12 (doi: 10.1093/gigascience/giab008)<br>https://github.com/samtools/samtools<br>HiSat2 version 2.2.1<br>http://daehwankimlab.github.io/hisat2/<br>VarScan version 2 (doi: 10.1101/gr.129684.111)<br>https://github.com/Jeltje/varscan2<br>Cutadapt version 3.5 (doi: 10.14806/ej)<br>https://github.com/marcelm/cutadapt<br>REDItools 2.0 (doi.org/10.1186/s12859-020-03562-x)<br>https://github.com/tizianoflati/reditools2.0<br>Graphpad Prism 9 for macOS version 9.3.1<br>Microsoft Excel version 15.32

For manuscripts utilizing custom algorithms or software that are central to the research but not yet described in published literature, software must be made available to editors and reviewers. We strongly encourage code deposition in a community repository (e.g. GitHub). See the Nature Portfolio guidelines for submitting code & software for further information.

Data

Policy information about availability of data

All manuscripts must include a data availability statement. This statement should provide the following information, where applicable:

- Accession codes, unique identifiers, or web links for publicly available datasets
- A description of any restrictions on data availability
- For clinical datasets or third party data, please ensure that the statement adheres to our policy

The data supporting the findings of this study are available within the paper and its Supplementary Information. Source data for the figures are provided with this paper. The NGS files generated in this study are available from the GEO database via the accession number GSE202643.

Field-specific reporting

Please select the one below that is the best fit for your research. If you are not sure, read the appropriate sections before making your selection.

☒ Life sciences ☐ Behavioural & social sciences ☐ Ecological, evolutionary & environmental sciences

For a reference copy of the document with all sections, see nature.com/documents/nr-reporting-summary-flat.pdf

Life sciences study design

All studies must disclose on these points even when the disclosure is negative.

Sample size	No statistical methods were used to predetermine sample sizes for the in vitro and in vivo experiments. Sample sizes were chosen on the basis of existing procedures and standards in the field and on the basis of biological replicates (with n values of at least 2). Whenever experimentally possible and reasonable, more replicates were performed.
Data exclusions	No data were excluded from the analyses.
Replication	Experiments with duplicates or triplicates of cells were done with distinct aliquots of cells at intervals of at least one week. A biological replicate in the in vivo experiments corresponds to an individual mouse. All experiments were repeated at least once. All attempts at replication were successful.
Randomization	To each DdCBE pair an ID number was attributed in a numerical order. Cells were treated with DdCBE pairs also in a numerical order.
Blinding	Samples were collected and analysed by NGS by independent researchers, without any description.

Reporting for specific materials, systems and methods

We require information from authors about some types of materials, experimental systems and methods used in many studies. Here, indicate whether each material, system or method listed is relevant to your study. If you are not sure if a list item applies to your research, read the appropriate section before selecting a response.

Materials & experimental systems

n/a	Involved in the study	
☐	☒	Antibodies
☐	☒	Eukaryotic cell lines
☒	☐	Palaeontology and archaeology
☐	☒	Animals and other organisms
☒	☐	Human research participants
☒	☐	Clinical data
☒	☐	Dual use research of concern

Methods

n/a	Involved in the study	
☒	☐	ChIP-seq
☒	☐	Flow cytometry
☒	☐	MRI-based neuroimaging

Antibodies

Antibodies used

Primary antibodies used:
1. mouse anti-NDUFB8, dilution 1:1000 (CN Abcam, ab110242)
2. mouse anti-SDHB, dilution 1:2000 (CN Abcam, ab14714)
3. mouse anti-UQRC2, dilution 1:1000 (CN Abcam, ab14745)
4. mouse anti-COX IV, dilution 1:1000 (CN Abcam, ab14744)
5. mouse anti-ATP5A, dilution 1:1000 (CN Abcam, ab14748)

March 2021

	Secondary antibodies used:<br>6. HRP-linked Goat Anti-Mouse IgG (Promega, W4021) - Western blot
Validation	Validation statements in the manufacturer's website:<br>1. mouse anti-NDUFB8: "Validated in WB, IHC and tested in Mouse, Rat, Cow, Human".<br>2. mouse anti-SDHB: "Validated in WB, IHC, Flow Cyt, ICC/IF and tested in Mouse, Rat, Cow, Human".<br>3. mouse anti-UQRC2: "Validated in WB, IHC, Flow Cyt and tested in Human". Validated in mouse in this study.<br>4. mouse anti-COX IV: "Validated in WB, Flow Cyt and tested in Mouse, Rat, Cow, Human".<br>5. mouse anti-ATP5A: "Validated in WB, IHC-P, ICC/IF, Flow Cyt and tested in Mouse, Rat, Cow, Human, Drosophila melanogaster"

Eukaryotic cell lines

Policy information about cell lines

Cell line source(s)	NIH/3T3 (ATCC; CRL-1658); Flp-In-3T3 (ThermoFisher, cn. R76107); 3T3 rho-0#8 (Kerafast, cn. ESA101)
Authentication	Cell lines were not authenticated for this study.
Mycoplasma contamination	The cell medium was tested for mycoplasma contamination. All tests were negative.
Commonly misidentified lines<br>(See ICLAC register)	No commonly misidentified cell lines were used.

Animals and other organisms

Policy information about studies involving animals; ARRIVE guidelines recommended for reporting animal research

Laboratory animals	Mice (Mus Musculus) in a C57BL/6J background were obtained from Charles River Laboratories. The animals were maintained in a temperature-and humidity-controlled animal-care facility with a 12-h-light/12-h-dark cycle and free access to water and food, and they were sacrificed by cervical dislocation.
Wild animals	The study did not involve wild animals.
Field-collected samples	The study did not involve samples collected from the field.
Ethics oversight	All animal experiments were carried out in accordance with the UK Animals (Scientific Procedures) Act 1986 (PPL: P6C20975A) and the EU Directive 2010/63/EU.

Note that full information on the approval of the study protocol must also be provided in the manuscript.
